# Immune Surveillance and Immune Escape in Cancer: Mechanisms and Immunotherapy

**DOI:** 10.1002/mco2.70321

**Published:** 2025-10-11

**Authors:** Ying Peng, Linsheng Zhan, Jianxiang Shi, Jie Wang, Yueying Li, Xiangdong Sun, Jie Lv, Huiyu Yang, Zan Qiu, Xingzhao Liu, Chenyan Li, Shanshan Gong, Wen Jia, Huiying Wang, Yuqi Zhao, Bin Zhang, Wei Guo, Jiancheng Guo, Jian Shang, Qianqian Zhou, Yanan Yang, Feng Gao

**Affiliations:** ^1^ Henan Institute of Medical and Pharmaceutical Sciences Zhengzhou University Zhengzhou Henan China; ^2^ Academy of Military Medical Sciences Institute of Health Service and Transfusion Medicine Beijing China; ^3^ Henan Key Laboratory for Helicobacter Pylori & Microbiota and GI Cancer Marshall Medical Research Center The Fifth Affiliated Hospital of Zhengzhou University Zhengzhou China; ^4^ The Research and Application Center of Precision Medicine The Second Affiliated Hospital of Zhengzhou University Zhengzhou Henan China; ^5^ Department of Immunology School of Basic Medical Sciences Anhui Medical University Hefei Anhui China

**Keywords:** antigen cross‐presentation, cancer immunosurveillance, cancer vaccine, conventional dendrite cell type I, immune escape, immune hub

## Abstract

Despite the tremendous amount of basic knowledge in cancer immunity gained and many transitional approaches attempted, current cancer immunotherapies are still far from reaching universal effectiveness. Therefore, next‐generation cancer immunotherapies would emerge from deepened mechanistic insights on the full spectrum of cellular and molecular interactions between cancer cells and their immune sentinels. This review embarks on an exhaustive exploration of the cardinal immunological principles that catalyze robust cancer surveillance and their potential escapes and recapitulate the state‐of‐art understanding of both receptors and corresponding immune cell types involved. Both tumor intrinsic and tumor microenvironmental mediators of immune escapes are outlined in the context of current clinic applications. Following emphasizing the exceptional requisites that effective cancer immunity cycle must meet, specific cellular subsets crucial for igniting tumor immunity, notably effector and helper T cells alongside antigen presentation cells are examined, focusing on their close interactions in both antigen‐dependent and ‐independent manners. Such intricate interactions form dynamic immune hubs at the tumor site, holding promising key functionality in rendering effective cancer retreat. Grounded on these recent insights, refined immunotherapeutic strategies, especially those bolstering priming based anticancer effector functions are advocated.

## Graphical abstract

Our review focused on how specific cellular subsets crucial for igniting tumor immunity cycle, notably effector and helper T cells alongside antigen presentation cells, interact closely in both antigen‐dependent and ‐independent manners (within centered ring) adjacent to cancer cells. Such intricate interactions form an integrated and dynamic immune hub at the tumor site, where a delicate balance between immune killing of cancer cells versus cancer immune suppression and escape is reached. To tilt the balance favoring cancer eradication, mechanistic insights inspired us to advocate for three specific refined strategies (on the peripheral of the ring) to bolster antitumor vaccine designs by ① delivering the antigen to draining lymph node, and to maintain engaged DC status within TME and DC's vital mobility back to the tumor draining lymph node (purple arrows), ② targeting the antigen to be engulfed by specialized cDC1 antigen presentation cells, and ③ promoting antigen cross‐presentation by engaging relevant cellular signaling pathways as well as purposefully shuttling endolytic protein cargoes through particular routs among diverse cellular compartments.

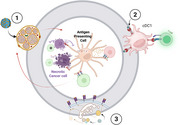

## 1 Introduction

Immunity, as a dynamic phenomenon shared by almost all living beings, is believed to arise independently multiple times during our natural history, by antagonizing against the persisting selection pressure to combat with invading “foreign” colonizers (mostly pathogens including viruses, bacteria, and eukaryotic parasites). The cohabitation of living organisms within our biosphere poses an ultimate evolutionary goal for any effective immunologic system: discriminating against “foreign” beings for eliciting an immediate and decisive attack versus “self,” which should be as “transparent” as possible in the eyes of immunological surveillance, in order to avoid internal conflict and consequential self‐destruction. This mechanism is extremely critical for the survival of multicellular eukaryotes, since a myriad of “self” cells with distinct characteristic and functionalities must cooperate in a living organism. The self‐inflicting wounds on any “self” cell population are likely to be extremely detrimental to the overall survival of the organism, since the division of labor among “self” cells requires the survival of most functional cells. Such revelation can be best appreciated by the prevalence of a wide range of “autoimmune disorders” in human, manifesting in diverse forms with our immune system attacking distinct “self” cells [1].

While the exact pathobiology of most autoimmune disorders remains poorly understood, it is acknowledged that the dysfunction of immune system can arise from a combination of genetic, environmental, and hormonal factors [2]; the precise discrimination of “foreign” versus “self” remains the critical founding principle of immunity. The idea coined “horror autotoxicus” was first formally pushed forward by Paul Ehrlich, who suggested that the immune system has to be inherently tolerant to the body's own tissues and does not attack them [3]. Therefore, for a long time, tumors were considered poorly immunogenic, meaning they were thought to produce few or no “foreign” antigens that the immune system could recognize. This belief was based on the observation that cancer cells are derived from the body's own cells, from which as we now understand via genetic and epigenetic changes [4].

Although early immunology pioneers such as Paul Ehrlich and Frank Mac Farlane Burne put forward the possibility “that small accumulation of tumor cells may develop and because of their possession of new antigenic potentialities provoke an effective immunological reaction with regression of the tumor and no clinical hint of its existence” [5] (best known as Burnet's theory of cancer immunity), skepticism against effective cancer immune surveillance was overwhelming throughout the past century. First, cancer arise despite the apparent immune competence of affected individuals. Second, early immunotherapies ranging from initial heat‐killed bacteria (literally Coley's toxins) to rationally devised cancer vaccines and cytokine treatments often failed to produce consistent and significant clinical outcomes [6, 7]. These overall failures reinforced the presumption that the immune system could not effectively target and eliminate cancer cells.

**FIGURE 1:**
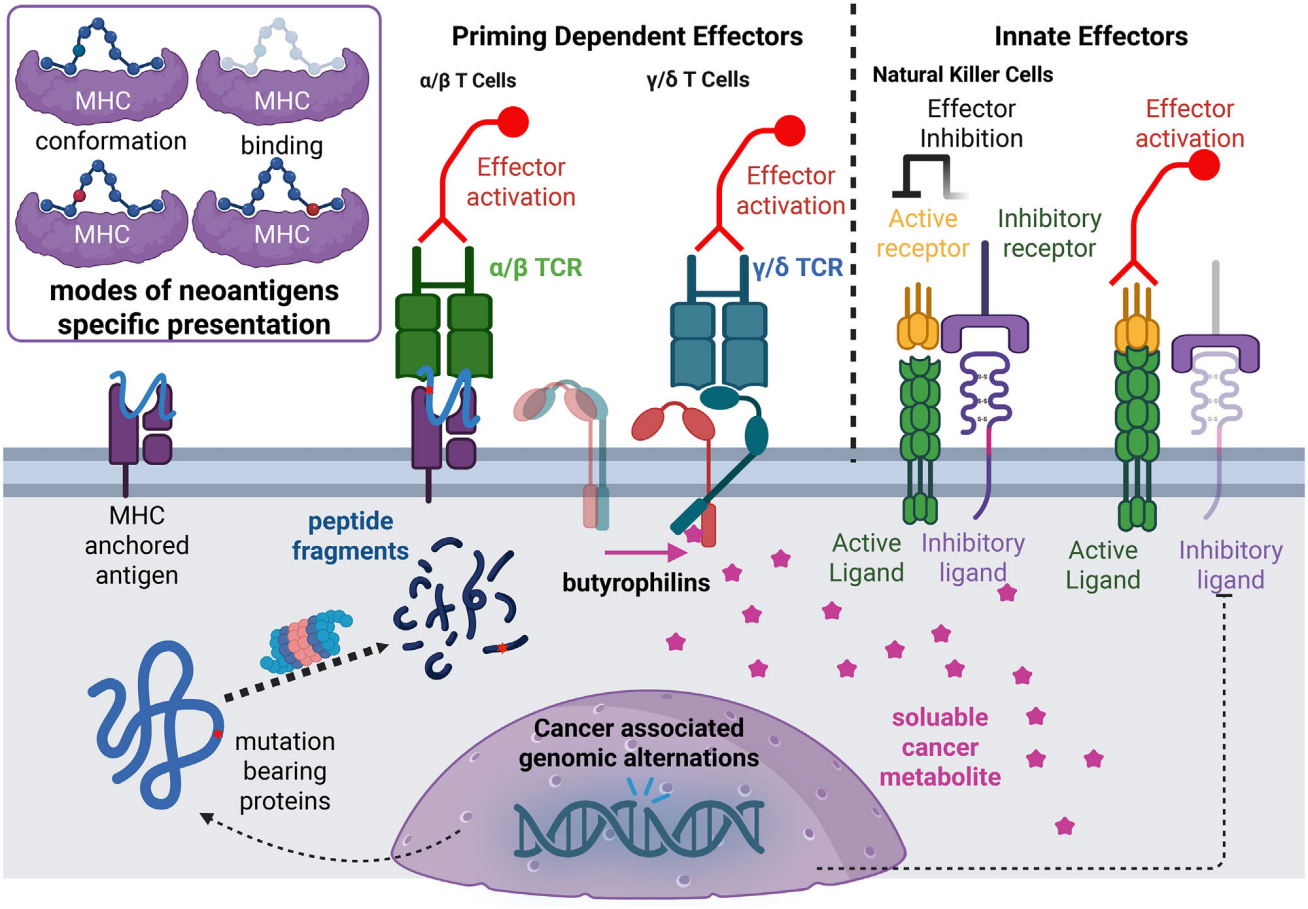
Surveillance receptors for immune detection of transformed cancer cells. There are two modes of immune detection of transformed cancer cells, by distinct immune cell types and through recognizing different cell surface molecules on transformed cells. (A) Priming‐dependent surveillance is mainly taken by T cells, with mostly by αβ T cells use their αβ TCR dimers to recognize neoantigens presented by MHC molecules. Neoantigens are mainly generated through proteolytic degradation of mutated polypeptides in the cancer cells. Once they are presented by MHC molecule, they are distinguished from unmutated peptides through two major ways (figure insert): they differ in complex spatial configurations or the neoantigens gain MHC binding capability by “anchoring” into MHC peptide binding groove. γδ T cells use their γδ TCR dimmers recognizing non‐MHC cell surface molecules. Depicted in the figure is a representative example of Vγ9Vδ2 TCR activated by so called “inside‐out” signaling, by small diphosphate metabolites (pAgs, purple stars) generated in cancer cells The sensing of pAgs involved ligand binding to the intracellular B30.2 and glues the intracellular domains of butyrophilins BTN3A1 and BTN2A1. Their intracellular interaction enables BTN3A1 extracellular ectodomain triggering Vγ9Vδ2 TCR binding in trans and activation of Vγ9Vδ2 T cell. (B) Innate surveillance is mainly executed by innate immune cells such as NK cells, which carry out immune surveillance using both “activator receptors” and “inhibition receptors.” On healthy cell surface, both active ligand and inhibitory ligand are present. Therefore, on the signaling cell end, the inhibitory signaling will typically overrule the outcome with no NK cell activation. However, if transformed cells miss the inhibitory ligand expression due to transformation, NK cell will be activated by the activation signaling alone. Besides NK cells, a small subset of T cells can use innate receptors for cancer surveillance as well.

The steady progress of molecular and cellular immunology over the past decades has established a solid and rich mechanistic foundation on immunological surveillance. We now know there are distinctive immune cells equipped with cytotoxic effector arsenals together with cell surface surveillance receptors [8, 9]. The cell signaling cascades composed with both positive and negative inputs from individual sensors are integrated at the single cell level regarding reaching a somewhat stochastic but binary decision on whether mobilizing available cytotoxic granules for target cell killing or not (Figure 1) [10, 11, 12, 13, 14]. Although the cellular logic on how bistability distinguishing “self” and “foreign” can be reliably calculated through cell signaling network remains poorly understood [15, 16, 17, 18], we now know there are diverse repertoire of different T cell receptors (TCRs) generated by somatic recombination and editing during development [19, 20], potentially enabling at least a minimal clone of cells catching most if not all potential “foreign” antigens encountered [21, 22, 23]. Particularly, since the discoveries of first immune checkpoint receptors in the 1990s, followed by the efficacy of cancer treatments via antibody‐mediated blockade of the immune checkpoint first demonstrated in 1996 [24], antitumor immunity, paradoxically remaining skeptical among physicians until then, only emerge from under the water with the widespread clinical applications of immune checkpoint inhibitor (ICI) since its first clinical approvals in 2011. It is widely accepted now there are three phases of immunoediting during tumor development [25]. During the elimination phase, transformed cells are destroyed under the surveillance of endogenous immune system. During the following equilibrium phase, cancer cells are under stronger and stronger elimination pressure since the specific cancer immune effector cells are expanding and activating. Therefore, they come up with adaptive mechanisms to diminish the local cytotoxicity of the effector immune cells. When sporadic genetic or epigenetic editing occurs, a minority of original tumor cells manage to survive immune destruction then enter a third and final phase of the process. During the escape phase, immunologically sculpted tumors begin to establish an immunosuppressive tumor microenvironment, and gradually gain clinical significance. When immune checkpoint blockade therapy was made available, the threshold for all available T cells to elicit attacks against corresponding antigen bearing cells are decreased. Therefore, the clinic success of long‐term disease‐free survival of patients under ICIs treatments suggested a shifted balance between cancer immune surveillance and tumor immune escape, unequivocally pinpointing the availability and effectiveness of endogenous cancer targeting immune cells rendering cancer elimination.

With the widespread applications of cancer immuno therapies, in particular ICIs and adoptive cell transfer (ACT), we envision a timely review extremely beneficial on understanding how an intertwined battle between cancer immunosurveillance and cancer immune escape dictates the clinic outcomes of individual patients. Therefore, we first summarized the known principles of how cancer immunosurveillance was carried out by different effector T cells via distinctive receptors. Next, on the flip side of the coin, we discussed mediators of T cell exhaustion and other major means of cancer immune escapes. The limitations of both ICIs and ACT were discussed on the context of what is missing in sustaining effector T cell number and activities with corresponding therapeutic strategies. In depth discussion on the recently emerged local immune‐hubs sustained by organic interactions between antigen‐presenting cells (APCs) with tumor antigen harvested and relevant antigen‐specific CD4+ and CD8+ cells reveals a new but exciting scene for cancer immunotherapy. Finally, we envisioned how rationally designed cancer vaccines can be an extremely appealing therapeutic avenue by fine‐tuning not only the quantities of cancer antigens but also the integrity of cancer immune‐hubs to sustain a fruitful cancer immune cycle.

## 2 Effector T Cells and Neoantigen‐Specific TCRs

T cells are the major sentinel immune cells attacking malignant transformed cells with diverse antigen specificity. We will first survey how they utilized their TCRs to distinguish transformed cells among healthy cells.

### 2.1 Tumor Mutational Burden, Neoantigens, and MHC Antigen Presentation

T cells, together with B cells, are power‐horsing adoptive immunity, sharing the amazing properties combining generating diverse receptor repertoire for recognizing potential antigens with the plasticity establishing “clonal expansion‐based memory.” Such combination allows the flexibility to adjust the specific clonal population dynamically upon antigen encounters; therefore, a future repeated infection with the same pathogen can be handled more efficiently. Successful vaccines take full advantage of the dynamic nature of adoptive immunity arm, to prepare the host organism with sufficiently expanded memory and effector T cells as well as specific antibodies, blunting the significant risk of devastating infection by the prospective pathogen.

Cytotoxic effector T cells, most widely known as CD8+ T cells, and major subsets of CD4+ T cells [26, 27, 28, 29, 30, 31, 32, 33], used their TCRs for cancer cell recognition [34]. As originally proposed theoretically by Lewis Thomas “that the immune system recognize newly arising tumors through the expression of tumor‐specific neoantigens on tumor cells and eliminate them, similarly to homograft rejection, maintaining tissue homeostasis in complex multicellular organism” [35], we now know that so called “neoantigens” are indeed recognized by host TCRs [36], as they are “presented” as short peptides bound on MHC molecules exposed on cell surface, in a manner highly consistent with foreign antigens presented by MHC molecules from xenografted tissues [36, 37]. All cells can present antigenic peptides generated from endogenous protein degradation clinched onto one of either two classes of MHC molecules, with antigens universally resting in a groove formed by an extracellular region of MHC antigen‐presenting molecules [38, 39, 40] (Figure 1). All nucleated cells express MHC Class I (MHC‐I) molecules presenting peptides mainly derived from endogenous proteins, while peptides derived from exogenous proteins are mainly presented by professional APCs via their significant expression of MHC‐II molecules. The lack of a significant “self” immune response toward “self” cells are mainly due to efficient clonal deletions against self‐responding T cell clones with strong TCR activation during T cell maturation process in the thymus [41]. Therefore, neoantigen peptides specifically arising from tumor mutational burdens (TMBs) (such as via generating nonsynonymous mutated amino acids) were not previously seen during T cell maturation within host thymus and is likely to be immunogenic given it can be processed and bound efficiently by one of the host's MHC molecules [37] (Figure 1). Similarly, neopeptides can be generated from noncoding genomic sequences, translation aberrations, and posttranslational abnormality in cancer cells [42, 43, 44], gene fusions [45, 46], cancer‐specific alternative splicing [47, 48, 49], RNA editing [50], and endogenous retroviruses expressed in cancer cells [51, 52]. Such potential sources of generating cancer‐associated neoantigens are likely to explode further through recent successes in immuno‐peptidomics technologies [53, 54, 55]. On the other hand, microbial‐derived peptides were also discovered for their cancer immuno‐reactivity, due to tumor‐associated microbes or chronic viral infection associated with cancer incidences [56, 57, 58, 59, 60].

High TMB (Tumor Mutation Burden), necessitating a significant variety of potential neoantigens, is associated with clinical benefit from current immunotherapy [61, 62, 63, 64, 65, 66]. However, the clinical efficacy of immunotherapy is far from being predicted by the quantity of neoantigens available [67]. For example, boosting immune response against a single shared neoantigen has been shown to be highly effective in tumor control [68]. Furthermore, although TMB is in general consistent with the responsiveness of ICIs therapy, there are much more distinctive T cell clones discovered against each neoantigens from immune checkpoint blockade responders among melanoma patients under immunotherapy comparing with nonresponders, suggesting that a rich TCR pool against neoantigens ensures at least some of those distinctive clones will be effective in harnessing tumor growth [69]. Among multiple TCRs against the same neoantigen, while TCR binding strength in general predicts the T cell activation probability in vitro [70], in vivo control of tumor does not necessarily favor the T cell clone with the strongest binding affinity toward the cancer neoantigen [71]. Therefore, a rich neoantigen‐specific, actionable T cell reportorial recognizing multiple potent patient‐specific MHC/neoantigen combination would in principle ensure higher probability of neoantigen‐dependent cancer cell killing, although the nature of mutation heterogeneity among different cancer cells in the same patient will be inflicted with differential immune surveillance pressures, therefore escaping cancer cells with the least immune pressure will likely develop further and progress [72, 73].

### 2.2 Effector T Cells Expansion Relies on Clonal Activation via Heterodimeric TCRs Recognizing Different Cancer Cell Surface Features

TCRs sense antigens as a heterodimer. In humans, in 95% of T cells, the TCR consists of an alpha (α) chain and a beta (β) chain, whereas in 5% of T cells, the TCR consists of gamma and delta (γ/δ) chains (Figure 1). Somatic recombination on corresponding TRA and TRB, TRG, and TRD locus is mainly responsible for creating TCR diversity on T cells [19, 20]. The intersection of these specific variable regions (V and J for the alpha or gamma chain; V, D, and J for the beta or delta chain) corresponds to the complementarity determining region 3 region that is important for peptide/MHC recognition. In contrast, γδ T cells adopt an alternative TCR architecture, consisting of γ and δ chains, granting them the ability to perceive a wider array of antigens in MHC‐independent manner [74, 75]. γδ T cells extend their sensing capabilities to include stress‐induced antigens, phosphoantigens, and others, all while circumventing the need for MHC‐mediated antigen presentation [76, 77, 78]. Vγ9Vδ2 T cells—a major subtype of human circulating γδ T cells—respond to multiple cancer and viral infected cells [79], through so called “inside‐out” activation by small diphosphate metabolites (pAgs) [80, 81]. The sensing of pAgs involved ligand binding to the intracellular B30.2 domain of BTN3A, a cell membrane localizing butyrophilin protein. Subsequently, pAgs glues the intracellular domains of BTN3A1 and BTN2A1, both of which are structurally similar butyrophilins. Their intracellular association enables BTN3A1 to be free from its BTN2A1 partner on the extracellular ectodomains, subsequently triggering Vγ9Vδ2 TCR‐mediating T cell activation [82, 83] (Figure 1).

### 2.3 Innate Cytolytic Activities with Neoantigen‐Independent Cancer Surveillance Mode Might be Independent with Their TCRs

Besides well‐characterized Vγ9Vδ2 TCR, how other γδ T cells are activated by prospective ligand remains poorly understood. Furthermore, neoantigen‐independent cancer surveillance mode has been recently uncovered with innate cytolytic activities against tumor cells [84], while whether their TCRs are responsible for tumor‐specific recognition remains to be explored. However, such tissue‐resident type 1 innate‐like T cells are expanded in the presence of precancerous lesions, with an apparent lack of T cell exhaustion as most conventional cytotoxic T cells do [85, 86]. Processing stimulation‐dependent clonal expansion capability without simultaneously compromising its effector function makes it an attractive target for developing future cancer immune therapeutics [87].

## 3 Effector T Cells are Refrained with Signaling Modules to Counter their Effector Function

Effector T cells, although critical for immune surveillance, are extremely susceptible to cell death with prolonged immune activation. In addition, sustained immune activation will lead to uncontrolled and excessive release of proinflammatory cytokines, causing systematic “cytokine storm” that leads to frequent multisystem organ failure and even death. We will discuss endogenous signaling and metabolic modules refraining T cell effector functionality, followed by current immune therapies relieving partially such “braking” mechanisms.

### 3.1 T Cell Exhaustion and Immune Checkpoint Receptors

During adaptive immune responses, naïve T cells exit quiescence and become activated with antigen encounter to undergo clonal expansion and differentiation, a process collectively initiated by at least three well‐established signaling events: TCR binding to cognate antigen presented on MHC molecules (Signal 1), costimulation largely mediated by CD28 ligation (Signal 2), and cytokines (Signal 3). The canonic model of T cell activation suggests once T cells receive these critical signals simultaneously, they are committed to proliferate and acquire cytotoxic potential immediately. With subsequent and continuous encounter of cells presenting their cognate antigen, those effectors are committed to kill target cells, despite they will gradually lose their capability to proliferate and kill (so called “exhaustion”) [88]. Such gradual functional decline of elicited immune reaction, is thought to be mediated by so called immune checkpoint receptors. Those are a series of molecules that are found on T cells upon antigen encounter and T cell activation to prevent overactivation of the immune system [89]. Over the past few decades, numerous immune checkpoint molecules have been identified, including but not limited to PD‐1, CTLA‐4, lymphocyte‐activation gene 3 (LAG3), T‐cell immunoglobulin, and mucin‐domain containing‐3.

**FIGURE 2:**
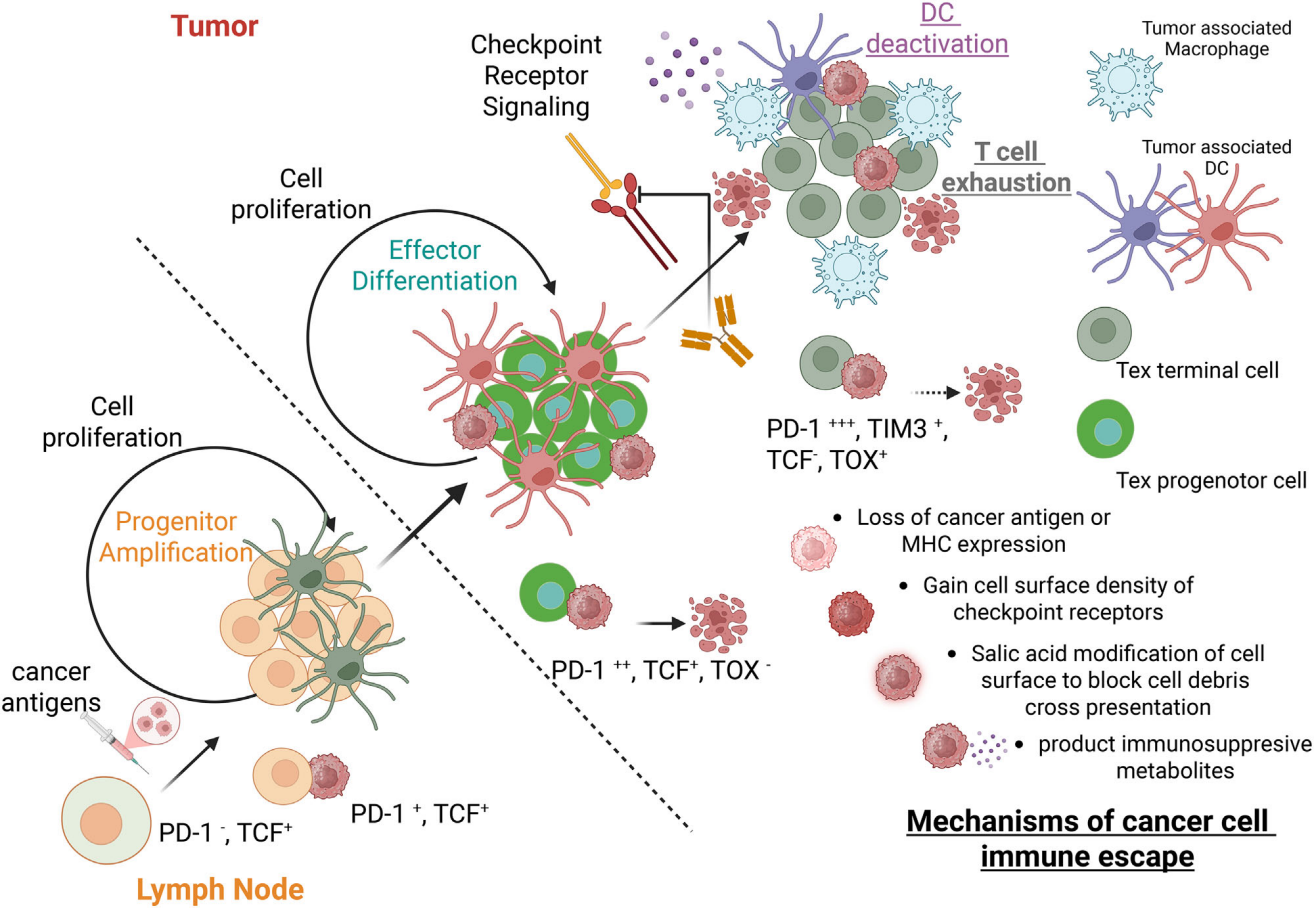
Mechanisms of cancer cell immune escape. Naive tumor antigen‐specific cells are originally stimulated within tumor draining lymph nodes with the the help of lymph node residual DCs presenting tumor antigen. The amplified progenitor T cells (orange colored) acquired PD‐1 expression upon antigen stimulation but lack significant effector program expression or cytolytic function against cancer cells (magenta colored). The progenitor T cells migrate into the tumor bearing tissue and were further stimulated by the abundant tumor antigen there. Their differentiation into Tex progenitor cells (green colored) which can acquire effector molecule expression and cytolytic functions against cancer cells (magenta colored). Tex progenitor cells can maintain self‐renewal capability within the tumor bearing tissue with the help of activated tumor‐associated DCs (magenta‐colored, star‐shaped cells) providing both antigens and appropriate costimulation signals. However, Tex progenitor T cells can be further exhausted (dark gray colored) via checkpoint receptor signaling mediated by both cancer cells (magenta colored) and tumor‐associated macrophages, as well as immunosuppressive metabolites. Therefore, Tex terminal T cells (PD‐1 high, TIM3 expressing, TCF negative, Dox positive) have limited cytolytic activity (dashed arrow) against cancer cells and cannot proliferate further. Tumor‐associated DCs can also be deactivated (purple‐colored, star‐shaped cells) by immunosuppressive metabolites; therefore, essential help to rejuvenate T cells is lost. Checkpoint receptor blocking antibodies can act on Tex progenitor cells preventing their further exhaustion in the presence of costimulation signals within TME. Besides this now well‐understood way of over presenting checkpoint receptors on cancer cells, there are at least three other ways cancer cells can escape immune surveillance: by losing its immunogenic tumor antigen expression or MHC antigen presentation machinery; by cell surface salic acid glycosylation to block cDC1‐mediated tumor antigen uptake and cross‐presentation, and by sequestering essential nutrients or releasing immunosuppressive metabolites. Those escaping cancer cells are labeled with distinctive shades of magenta color.

T cell exhaustion is a differentiation state characterized by progressive and hierarchical loss of effector functions following chronic antigen exposure [90, 91]. First characterized via chronic infection models, there are now overwhelming evidences that T cell exhaustion features are ubiquitously present in both human and mouse Tumor infiltrating T cells, as the consequence of chronic exposures to tumor‐associated antigens (TAAs) within TME [92, 93]. Following the antigen exposure of naive T cells, there are at least three cell subpopulations acquiring different degrees of T cell exhaustion features (Figure 2). T progenitor cells express low level of PD1 and high level of T cell proliferation marker TCF1, without significant expression of T effector program molecules such as CD39 and GZMB. Tex progenitor cells maintain expression of PD1 and T cell proliferation marker TCF1, but acquire the full expression of T effector program molecules. Both T progenitor cells and Tex progenitor cells are antigen experienced, but can maintain self‐renewal in vitro with the condition of antigen stimulation. Tex progenitor cells can subsequently differentiate toward Tex terminal cells within TME, which are characterized by loss of TCF1 (and consequentially substantial cell proliferation capability), and instead express high level of T cell exhaustion markers TOX, PD1, and T cell Ig and mucin domain‐containing protein 3 (TIM3) [94, 95, 96, 97]. Therefore, Tex terminal cells might have limited cytotoxic capability but are nonreplenishing [94, 98, 99].

The switch from TCF1 toward TOX expression largely defines a “definite” exhausted state, both of which are acting as master transcriptional regulators responsible for distinct T cell physiology [100, 101]. The increasing expression of exhaustion‐related checkpoint receptors such as PD1, and TIM3 are directly responsible for fine‐tuning the activation thresholds of T cell, making the same antigen‐specific T cell cell more and more refractory to be actively engaged in target cell killing with identical antigen encounter [88].

After ligand engagement with PD‐L1 expressed on cancer cells or APCs, PD‐1 becomes phosphorylated on its cytoplasmic ITIM and ITSM motifs to deliver inhibitory function. Among these two, the phosphorylation on ITSM but not ITIM might be critical, as tyrosine mutations of ITSM but not ITIM abrogates the inhibitory function of PD‐1 [102]. Phosphorylated ITSM primarily recruits phosphatase SHP2 to dephosphorylate key signaling molecules to downmodulate activation level [103, 104, 105]. In exhausted T cells with high PD‐1 expression, activated PD‐1 molecules form microclusters, inhibiting both antigen and costimulatory signaling, abrogating phosphorylation of TCR and its downstream signaling molecules like ZAP70 [106, 107].

Costimulation signal for T cell activation is mainly mediated by CD28 ligation on T cell surface via binding with CD80/86 expressed on APCs. Compared with CD28, T cell expressing CTLA‐4 binds to CD80/86 with much higher affinity, thus inhibiting costimulation via ligand competition [108]. At the same time, CTLA‐4‐expressing T cells can also reduce cell surface CD80/86 density on adjacent APCs by trans‐endocytosis, resulting in decreased CD28 signaling. Depending on the degree of T cell activation, CTLA‐4 can cluster into the immune synapse similar to PD‐1 [106, 109]. Within such inhibitory signaling microclusters, the YVKM motif of CTLA‐4 can be tyrosine phosphorylated, which in turn prevents the interaction between CTLA‐4 with AP‐2, therefore maintaining CTLA‐4 on the cell surface to boost inhibitory signaling [110], On the other hand, the phosphorylated YVKM motif can directly recruit SHP2 to repress T cell activation similar to the mode of action as PD‐1 [111]. CTLA‐4 also induces retrograde signaling in dendritic cells (DCs) through ligation of CD80/86, resulting IDO (indoleamine 2,3‐dioxygenase) expression and subsequent tryptophan depletion and severe T cell suppression [112].

TIM3 is another major checkpoint receptor highly expressed on exhausted T cells. Similar to PD‐1 and CTLA‐4, it is recruited to the immune synapse upon ligand engagement, disrupts stable immune synapse formation, and associates with receptor phosphatases [113]. There are four ligands reported for TIM3, including Galectin9 (C‐type lectin galectin9), Ceacam1 c (arcinoembryonic antigen cell adhesion molecule 1), HMGB1 (high‐mobility group box 1), and phosphatidylserine (PS). Ceacam1 binds to TIM3 both in cis and trans. The cis interaction with Ceacam1 in T cell is necessary for TIM3 glycosylation and surface expression, while their trans interaction‐mediated inhibition of effector T cell function [114]. Beyond T cells, TIM3 is also involved in DC function such that HMGB1 bound to TIM3 on tumor‐associated DC surface to suppress the recruitment of released nucleic acid from dying tumor cells, thus inhibiting nucleic acid‐induced innate immune response in DC, damping its costimulatory function [115]. Furthermore, TIM3 expressed on DC and macrophages promotes efferocytosis of apoptotic cells via direct PS binding. Therefore, TIM3 antibody inhibited engulfment of apoptotic cells by APCs, thereby reducing antigen cross‐presentation [116, 117].

LAG3 is identified as a ligand of MHC‐II and fibrinogen‐like protein 1 [118, 119]. The cytoplasmic domain of LAG3 contains a KIEELE motif, and multiple EP repeats. The KIEELE sequence is essential for the inhibitory function of LAG3 in T cells. Immediately preceding the KIEELE sequence, an evolutionary conserved basic residue‐rich sequence (BRS) motif found within the juxta‐membrane segment of LAG3. It was found to interact with acidic phospholipids abundant on the inner leaflet of plasma membrane. Such interaction efficiently sequesters the KIEELE signaling elements of LAG3 in T cells during quiescent state [120]. Upon T cell activation and LAG3 ligand engagement. The lysine residue on LAG3 cytoplasmic KIEELE motif is quickly ubiquitinated. While such ubiquitination does not affect LAG3 protein stability nor its cell surface presentation, it effectively relieves the plasma membrane sequestration of the KIEELE signaling elements [120], although its downstream signaling cascades remain elusive.

BTLA (B‐ and T‐lymphocyte attenuator) contains ITIM and ITSM motifs as well as a Grb2 recognition motif in its cytoplasmic domain. Both ITIM and ITSM can be phosphorylated to inhibit T cell function upon ligand ligation [121]. Despite the similarity between BTLA and PD‐1‐mediated phosphatase attenuation of TCR signaling, BTLA prefers to recruit the more potent phosphatase SHP1, therefore inhibits both TCR and CD28 signaling more effectively, while PD‐1 mainly recruits the weaker phosphatase SHP2 [105, 122, 123, 124]. In addition, T cell lineage protein THEMIS is recruited to the cytoplasmic domain of BTLA and blocks its signaling capacity via oxidation of the catalytic cysteine of the tyrosine phosphatase SHP‐1 [124]. Therefore, in the absence of THEMIS, the signaling capacity of BTLA is exacerbated. Under physiological condition, BTLA inhibitory signaling is fine‐tuned according to the THEMIS expression level, making CD8+ T cells more resistant to BTLA‐mediated inhibition than CD4+ T cells [124].

While the list of additional checkpoint receptors is still accumulating, it is likely that different receptors have distinct mechanisms to tune down T cell activation‐dependent effector function. The potential complex interplay between those damping activities with TCR signaling, coactivator signaling, and cytokine sensing is beyond the scope of this review. It is worth noting that checkpoint receptor signaling likely involved in the epigenetic changes to restrain effector T cells in a “locked‐in” exhausted state with genome wide chromatin level changes [125]. Whether such epigenetic transition can be reversed by applying checkpoint inhibitors remains a critical question to be addressed in the field of cancer immunology.

### 3.2 Both Cancer Cells and APCs in Tumor Microenvironment can Directly Engage Checkpoint Receptors Upon T Cell Encounter Leading to its Immune Escape

The activation of checkpoint receptors (to mediate T cell exhaustion) depends on the encounter of their cognate ligands on interacting cells. As we have discussed on the previous section, there are a wide variety of protein and lipid ligands for different checkpoint receptors. Therefore, the potential source of those ligands can be quite diverse. Traditionally, it is thought within the tumor microenvironment, cancer cells are the major cell type applying those brakes on T cells through activating checkpoint receptors on tumor‐infiltrated lymphocytes (TILs). In human cancer, elevated expression of checkpoint receptor ligands such as PD‐L1 is widespread in various tumor biopsy among many cancer types [126]. Therefore, a natural deduction for this phenomenon is when cancer cells are under increasing pressure of immune surveillance, epigenetic reprogramming of the cancer cells to increase their checkpoint ligand expression can effectively evict immune surveillance through damping the cognate TCR‐dependent T cell activation. This notion parallels with the observations that in many human cancer biopsies, loss of MHC‐I or II expression/maturation is frequently observed, which directly making the cancer cells “invisible” to T cells with antigen‐specific TCR surveillance.

Recent detailed examinations seems to challenge this cancer cell self‐centered view in causing TIL exhaustion. In GBM and other intracranial solid tumor models at least, TAMs seems to be a necessary cell type causing TIL exhaustion [127] (Figure 2). When TAMs have decreased antigen presentation capacity due to conditional IRF8 ablation, tumor control is significantly enhanced [128]. This is consistent with an independent study demonstrating the loss of MHC‐I ex vivo on TAMs led to decreased accumulation of Tex terminal cells [129]. Importantly, TAM depletion alone can decrease the frequency of Tex terminal cells in vivo and enhances anti‐PD1 response with different mouse tumor models [129].

Beyond the exhaustion program, the fine balance between Tex progenitor cell maintenance and Tex terminal cell differentiation is also keep by an active progenitor renewal program within TME [130] (Figure 2). It has been demonstrated that antitumor Tex progenitor cell can be sustained by optimal TCR engagement, without which accelerated terminal differentiation is favored [131]. Similarly, MHC‐II‐restricted antigen presentation is required to prevent dysfunction of cytotoxic T cells by blood‐borne myeloids in brain tumors [132]. Although a definite cell type providing such TCR engagement remains unresolved (since both cancer cells and many APCs can be the source of tumor antigens), sustained Tex progenitor renewal is coupled to their proximal positioning to DC niches [131]. Therefore, although different APC have different capacities for cross‐presenting tumor antigens to engage antigen‐specific T cells, some cell types are more likely to promote T cell exhaustion (likely through extensive engagements with checkpoint receptors or generating immunosuppressive metabolites) while other cell types are helping Tex progenitors to maintain renewal capability and effector functions, likely through elevated costimulation and favorable cytokine signaling. Additional cell surface interaction modes between different APC and T cells likely participate in this function divergence beyond well‐established immune receptors [133, 134]. Such difference might be largely cell intrinsic, since in vitro TAMs sustain longer, interactions with CD8 T cells than equivalent DC–CD8 T interactions, while the the degree of cognate TCR activation is much weaker in the TAM–CD8 T cell pair [127].

### 3.3 Metabolic Sabotage: Nutrient Competition and Immunosuppressive Metabolites

Beyond the well‐established signals 1–3 for T cell activation, recent investigations have unveiled that T cell metabolism acts as an additional but critical axis for T cell functionality [135]. Current consensus merging from an increasing number of studies suggests metabolic condition favoring increased mTORC1 signaling, c‐MYC activity, and activation of biosynthesis pathways altogether driving T cell activation with the other three traditional signaling axis. To be specific, glucose and amino acid uptake into T cells promotes mTORC1 activation; while glucose or glutamine deprivation activates AMPK instead. AMPK activation increases glutaminolysis and reduced mTORC1 signaling during glucose deprivation. Similarly, low amino acid uptake impair mTORC1 signaling and increase the number of uncharged tRNAs, which in turn drives fertile protein biosynthesis [135].

Within the TME, nutrient competition arises from the simultaneous uptake of carbohydrates, lipids, and amino acids from a common extracellular pool by many residual cell types. Metabolic reprogramming of the cancer cells especially can effectively deprive glucose and amino acids (most prominently glutamine and methionine) from TIL via increasing cancer cell glucose and amino acid uptake and metabolism. IDO and ARG1‐expressing DCs and TAMs, and IDO‐expressing MDSCs, catabolize tryptophan and arginine through the kynurenine pathway (KP), thus leads to amino acid depletion [136]. On the other hand, cancer cells and some innate immune cells can release various immunosuppressive metabolites to dampening antitumor immunity, among which the potent ones include adenosine, kynurenine, prostaglandin E2 (PGE2), and norepinephrine and epinephrine [137].

Adenosine acts mainly by agonizing four G protein‐coupled extracellular receptors, A1AR, A2AAR, A2BAR, and A3AR, stimulating cAMP‐dependent inflammatory inhibition pathways and suppress immune response. In CD8+ T cells, for example, adenosine‐mediated A2AAR agonism reduces cytotoxicity, inflammatory cytokine production, and TCR signaling [138].

Norepinephrine and epinephrine bind two distinct classes of G‐coupled protein receptors: α‐adrenergic receptors (α1 and α2) and β‐adrenergic receptors (β1, β2, and β3), all of which can be expressed by immune cells. β‐adrenergic receptors are on the top of canonic cAMP–PKA–CREB signaling cascade. Therefore, it is unsurprising that norepinephrine and epinephrine has potent inhibitory effects on antitumor immunity [139].

Kynurenine is the major metabolite produced by cancer cells and some innate immune cells through the KP, which itself causes tryptophan nutrient deprivation. Kynurenine is a potent activator for AhR (Aryl hydrocarbon receptor) [140]. Upon ligand binding, the ER residing AhR/AIP (AhR‐interacting protein) complex sheds AIP, allowing nuclear translocation. In turn, freed AhR forms a heterodimeric transcription factor with the AhR nuclear translocator (ARNT) in nucleus to  alters genome wide gene expression. In particular, the kynurenine–AhR axis strongly inhibits the activity of CD8+ and CD4+ T cells [141]

PGE2's role through PGE receptor EP2 and EP4 signaling has been well established in immunosuppression. In CD8 T cells, EP4 and EP2 are upregulated upon TCR activation. PGE2 in TME can blunt T cell inflammatory cytokine signaling by repressing Il2ra, which in turn downregulates c‐Myc and PGC‐1 activities, impairing T cell expansion and antitumor activity [142]. EP4 and EP2 are also expressed in various myeloid cells. In particular, PGE2 signaling promoting a tolerogenic phenotype in DCs that impairs tumor immunity, which we will focus further in subsequent sections [143, 144].

### 3.4 Immune Checkpoint Blockade Therapy: From CTLA‐4 to Next‐Generation Targets

Antibody‐mediated blockade of the immune checkpoint was quickly translated in clinic oncology since its first experimental demonstration of efficacy in 1996 [24]. Anti‐CTLA‐4 monoclonal antibodies ipilimumab (Bristol‐Myers Squibb) and tremelimumab (Pfizer, MedImmune) were the first ICIs to enter clinical testing, with the approval of ipilimumab in 2011 as first‐line therapy for advanced unresectable melanoma, Meta‐analysis done on long‐term follow‐ups with a large number of ipilimumab treated melanoma patients revealed durable survival in approximately 20% of patients [145]. However, in nonmelanoma cancers, Ipilimumab as a mono‐therapy has so far only shown marginal antitumor effects [146]. Furthermore, among the patients undergoing ipilimumab treatment, “immune‐related adverse events” (irAEs) was recognized and characterized as a spectrum of frequent accompanying side effects. These irAEs were associated with spontaneous inflammation in normal tissues, resembling pathology observed in human CTLA‐4 heterozygotes [147].

In contrast to anti‐CTLA‐4, anti‐PD‐1/PD‐L1 blocking Abs appear to have a much broader spectrum of antitumor activity in the clinical setting. Antitumor activity has been observed with almost all epithelial cancers (lung, head and neck, and bladder cancers, among others) established the PD‐1 pathway as a dominant key target in cancer immunotherapy. Despite these promising clinical results, the majority of cancer patients treated with anti‐PD‐1 or anti‐PD‐L1 monotherapies fall short of achieving objective responses, and most tumor regressions cases are partial rather than complete. Therefore, current clinical practices are oriented around employing PD‐1 pathway blockade combinatory therapies together with anti‐CTLA‐4 or other checkpoint inhibitors, chemotherapy, targeted therapies, focal radiotherapy, cancer vaccines, or ADCs [148, 149].

One exemplary combination therapy comes first from animal models. A strong synergistic immunosuppression effect by LAG3 and PD‐1 came first from double‐knockout mice that succumb to autoimmunity and early death although LAG3 or PD‐1 single knockout animals do not display obvious spontaneous lymphocytic infiltration [150, 151]. In addition, most tumors xenoplanted in PD‐1/LAG3 double knockout mice were completely rejected, while similar transplantation on PD‐1 single knockout mice mostly display delayed tumor growth, reinforcing the notion that LAG3 and PD‐1 are synergistic in regulating T cell activity [151]. Such animal models eventually led to the recent approval of the relatlimab (a LAG3‐blocking antibody)/nivolumab (anti‐PD‐1 antibody) combination for the treatment of unresectable or metastatic melanoma by United States Food and Drug Administration, establishing LAG3 as the third checkpoint inhibitor with demonstrated clinical efficacy. A phase 2/3 randomized trial reported that the anti‐LAG3 therapeutic relatlimab, in combination with nivolumab, achieved significantly larger portion with 12‐month progression‐free survival (PFS) in patients with melanoma, compared with nivolumab monotherapy [152]. Far fewer patients had severe irAEs compared with ipilimumab/nivolumab, while the oncology response profile of opdualag (relatlimab/nivolumab) is comparable to the ipilimumab/nivolumab (anti‐CTLA4/anti‐PD‐1) combination [152]. Thus, given the superior toxicity profile, risk:benefit ratio, and survival profile, opdualag (relatlimab/nivolumab) might become the preferred first‐line therapy in advanced melanoma and many other solid tumor.

Despite the continuous advances of ICI clinic applications, there remains a small portion of cancer patients under current therapeutic combinations can reach full tumor regressions. This limitation urges mechanistic re‐examination of immune checkpoint receptor‐mediated T cell biology. There are now widespread evidences that Tcf1+ CD8+T cells are the primary cells that proliferate in response to ICI therapy, while the TCF1‐DOX+ Tex terminal T cells cannot regain proliferation capability under ICI therapy [94, 99, 153, 154]. Importantly, T cell costimulatory receptor CD28 is a primary target for PD‐1‐mediated inhibition [103] and CD28 activation is essential for CD8+ T cells to respond to PD1 blockade in the context of tumor control [103]. It is well known in “immune‐cold” tumors, low T cell infiltration is associated with far fewer costimulatory molecules in TME, and such “immune‐cold” cases are largely unresponsive to Immune checkpoint blockade therapy [155, 156]. While costimulatory signals can be provided by activated APCs in the draining LN, recent evidences demonstrated that stem‐like CD8+ T cells still need costimulation persisting within the tumor for proliferation and more importantly effector differentiation [133]. Thus, within an “immune‐cold” tumor, ICI is unlikely to ignite effective antitumor immunity by itself. A logical complementary approach is to to activate tumor residing DCs responsible for harvesting tumor antigens from dying cells and presenting those to tumor infiltrating lymphocytes. On the other hand, approaches such as oncolytic viral therapies or local TLR ligands therapies have shown promising efficacy at least in animal models, suggesting boosting general tissue inflammation alone can elevate specific tumor immunity [157]. Future exciting progresses should be expected on translating those complementary approaches on different oncology clinic settings to achieve improving tumor regression and survival profiles while minimizing toxicity and associated irAE risks.

## 4 Effector cells and Effector Cell Transfusion Therapies

Beyond immune checkpoint blockades, ACT is another popular class of cancer immune therapies. We will differentiate different flavors of effector cells useful for ACT and discuss current status and limitations of effector cell transfusion therapies.

### 4.1 Priming‐Dependent Effector Cells versus Innate Effector Cells

While activation of αβ T cells as we discussed above demands at least a tandem input of signals‐antigen recognition, costimulation, as well as cytokine signaling, γδ T cells can be activated by a singular signal from the target cells [62]. This distinction is critical since the first mode of effector activation is usually termed “primed activation” and the coactivator free mode is coined as “innate activation,” which is observed beyond above mentioned γδ T cells (Figure 1). NK cells, for example, are important innate immune effector cells without recombination generated target recognition receptors. They employ both germline‐encoded, activation and inhibition surveillance receptors (Figure 1). Upon target engagement via those receptors, a drastic increase of activation signal or an absence of “self” sensing, inhibitory signal, activates NK cells followed by the similar formation of immune synapse and cell polarization as during T cell activation [158, 159, 160] (Figure 1). They are effective in killing some cancer cells and viral‐infected cells by releasing two types of preformed cytotoxic protein: the granzyme and perforin. The highly polar release of the effector molecules cross the immunology synapses ensures selective killing of target cells both in vitro and in vivo [160]. Beyond NK cells with such well‐established innate cancer surveillance function, we now know a small portion of tissue‐resident T cells (both αβ and γδ) use cell surface innate receptors to screen and eliminate cancer cells [84, 85]. Their activation and expansion are dependent on the release of IL‐15 from the cancer rather than specific known tumor antigens [85]. Therefore, among multiple immune cell types with direct effector function against cancer, ILC1s are found to increase their numbers in tissue with cancer unlike NK cells, their enrichment does not accompany a strong TCR‐dependent bias as typical TILs (typical T cells), suggesting an antigen‐independent local amplification process is involved [85]. This notion is confirmed by their apparent lack of cell circulation as typical T cells do [84]. Given the notion that priming‐dependent effector cells are dynamically regulated in our immune system, most vaccination strategies have focused on providing such effective initial “priming” to boost the quantity and quality of priming‐dependent effector cells in order to better catch cancer cells. On the other hand, effector cells can be expanded and engineered in vitro and transfused back to the host to combat cancer.

### 4.2 Adoptive Cell Therapy were Done via Amplifying Effector Immune Cells in Vitro and Infused Back to the Patients

In line with the identification of those cancer surveillance cell types, adoptive cell Transfer (ACT) was done via amplifying T cell or NK cells in vitro and infused back to the patients [161, 162, 163]. Such therapeutics have reached important milestones in rendering cancer retreat, confirming the specific and independent cancer surveillance function by these effector cells. Although ACT therapy holds great promises, it has shown little progress in promoting clinic efficacy against solid tumors [62, 164, 165, 166, 167, 168, 169]. The complication of collecting patients’ own effector cells and engineering/expanding those in vitro with sufficient quality/quantities comes with the inherent drawback of high manufacture cost and complications [165]. Therefore, more creative ways of boosting both the number and function of those cancer combating effector cells within the individual's physiological state will open new doors of cancer immunotherapy.

### 4.3 The Limitations of ACT Therapies Indicate Other Key Cell Types Beyond the Well‐Established Cytotoxic Effector Cells Providing Critical Cancer Killing Function

Notable examples include the dramatic clinical benefit of chimeric antigen receptor (CAR) gene‐modified T cells redirected toward CD19 in patients with B‐cell malignancies [170] as well as the treatment of various autoantibody‐mediated autoimmune diseases [171]. In the cases of solid tumor, significant cellular heterogeneity is usually the norm, limiting the efficacy of using T cell/NK cells with a uniformly engineered antigen recognition modality to eradicate heterogeneous cancer cells. Additional known factors mitigating the efficacy of ACT include limited in vivo T cell persistence, poor tumor infiltration, and especially rapid loss of their effector function [162, 167]. Exhaustion of the endogenous, as well as in vitro amplified immune cells within the complex, immune‐suppressive tumor microenvironment has been widely documented in various human solid tumors and was thought to be a major roadblock for cancer immunotherapy [29]. Recent evidence suggested effector cell infiltration into mechanic stiff solid tumor will trigger exhaustion via epigenetic reprogramming [172]. Available ICI therapies can partially overcome this blockade in certain instances. However, ICI is coming with significant risk of triggering severe autoimmune responses as discussed previously [173], while its clinical efficacy as a monotherapy or in combination with ACT remains limited. Therefore, there are likely other key cell types beyond the well‐established CD8+ cytotoxic cells providing direct cancer killing function or critical supportive role for their sustained activity.

There have been accumulating studies on the difference of tumor infiltrating lymphocyte status comparing cancer patients responsive to ACT therapy and those unresponsive [93, 174, 175]. A memory‐progenitor stem‐like CD8+ TIL population has been found closely associated with complete cancer regression for ACT transfused melanoma patients and long‐term T cell persistence in the patient following the cell transfusion [130, 175, 176, 177, 178]. Such studies highlight there are additional factors maintaining an “active” cancer immunity hub for highly efficient immune cell therapy, involving the persistence of appropriate TCR‐dependent and ‐independent stimulation to maintaining those stem‐like CD8+ cells in an optimal epigenetic state [179].

## 5 Cancer Immunity Cycle with Cancer‐Derived Antigen Processing as its Core Process

The notion that additional cell types beyond effector cells are critical for cancer immune retreat is best put forward within the framework of cancer immune cycle. This concept is distinct from the oversimplified “recognize and attack” model of cancer immune surveillance by recognizing the natural balance between cancer cell proliferation and escape, cancer immune suppression, and cancer cell necrosis from immune attacks. The latter, positive leg of the cancer immune cycle is to provide the “fuel” for igniting cancer immunity further. Critical engines processing and consuming such fuel are so called APCs.

### 5.1 Specialized Antigen Collection From the Dead Cancer Cells for Efficient Antigen Presentation Initiates Cancer Immunity Cycle

Concurring with this view that much more is involved than the traditional TCR–antigen recognition‐based cancer surveillance and elimination model, we now understand the iterative nature of the cancer cell recognition and killing: specialized antigen collection from the dead cancer cells for efficient antigen presentation, followed by maintaining an integrated functional immune engagements adapting in real time with tumor evolution [180]. Any step of such “cancer‐immunity cycle” can become rate‐limiting, rendering the immune system unable to cease the tumor growth with sufficient speed during the chronic process. For example, ICI therapy can lift the brakes on T effectors cells, increasing their cancer killing activity to a given extant. In some cases, this limited boost can restore the full engagement of such a “cancer‐immunity cycle,” therefore those patients can be benefited from PFS and even complete cancer regression [181]. However, this is not always the case, suggesting future cancer immune therapy should be targeting other critical rate‐limiting steps on the “cancer‐immunity cycle” as we discussed in previous section.

### 5.2 When Antigen From the Dead Cancer Cells are Processed, Cross‐Presentation is Tightly Regulated and are Specially Potent in Promoting Cancer Immunity Cycle Progression

**FIGURE 3:**
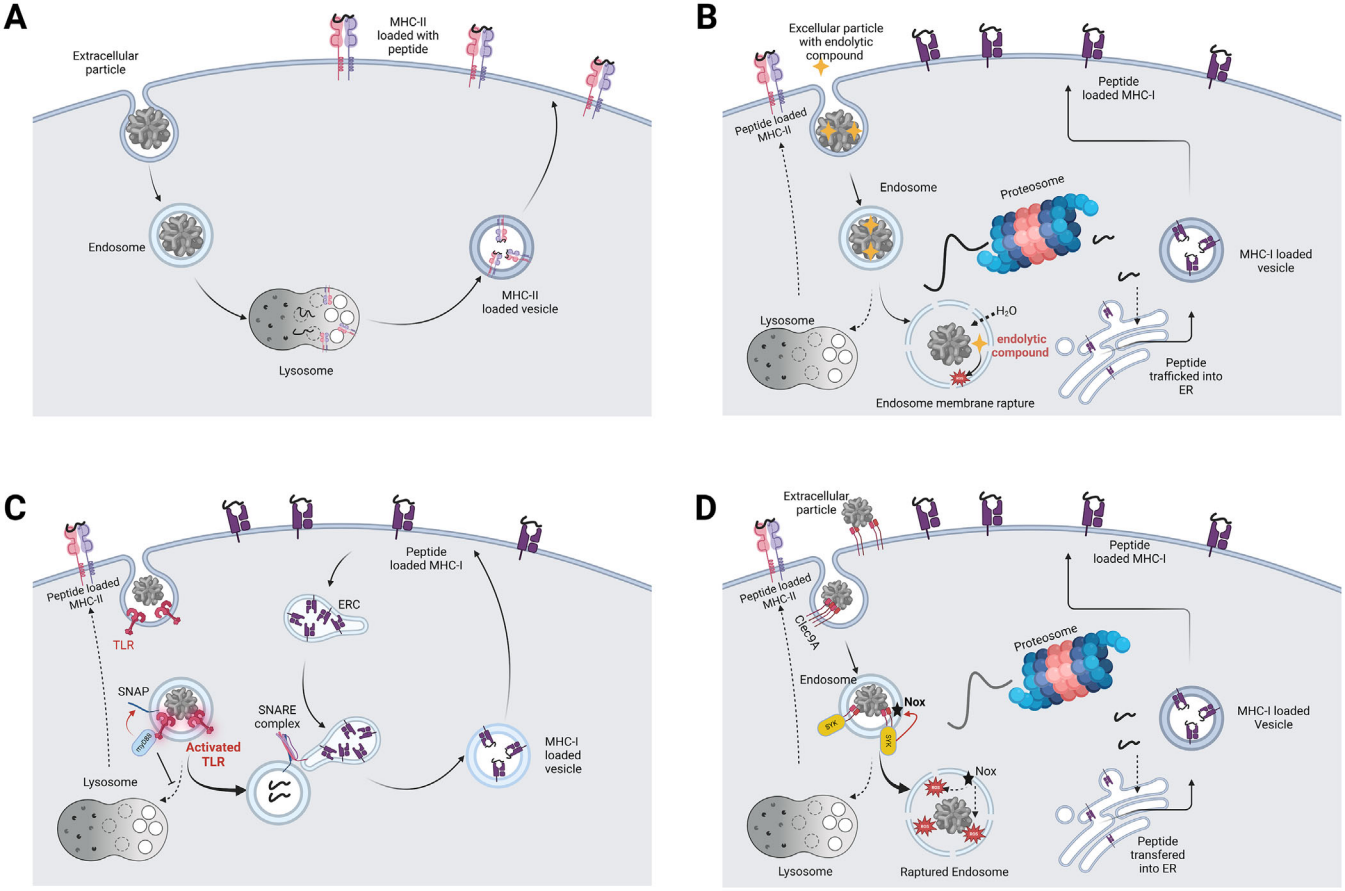
Default antigen presentation mode for exogenous antigens and potential antigen cross‐presentation mechanisms. (A) Exogenous antigens are taken up by antigen‐presenting cells via endocytosis or phagocytosis and enclosed in endosomes or phagosomes. The antigen is broken down into peptides mainly within lysosomes. These peptides are then subsequently loaded onto MHC‐II molecules in lysosome‐derived vesicles and transported to the cell membrane, where they are presented mainly by MHC‐II molecules. (B) Endolytic molecules (yellow stars) can facilitate antigen cross‐presentation. Endolytic molecules can be uptake together with exogenous antigens. Once in endosomes or phagosomes, such compound can facilitate membrane rupture of vesicle membrane, by either increase osmetic pressure or promoting free radical production damaging vesicle membrane. The consequences of both processes include the accessibility of the cargo by cytoplasmic proteosomes. Their processed peptides are transported into the endoplasmic reticulum (ER) via the TAP (transporter associated with antigen processing) transporters. Once in the ER, the peptides are loaded onto MHC‐I molecules. The MHC‐I–peptide complex can be transported to the cell surface via ER–plasma membrane vesicle transit system. Antigens in intact endosomes undergo parallel lysosome‐mediated pathway for MHC‐II‐dependent peptide loading as in (A). (C) Cargo uptake‐related TLR activation can facilitate antigen cross‐presentation. Some exogenous cargo contain pathogen‐associated molecular patterns molecules that can ligate and activate TLR sensors when the cargo undergoes endocytosis. The phagosome with activated TLR/Myd88 complex facilitates membrane fusion with MHC‐I enriched ERC compartment, via phosphorylation‐dependent SNARE complex assembly, thereby short‐cutting the antigen‐derived peptides back to the plasma membrane in an MHC‐I‐bound format. Antigens in endosomes without ERC hijack undergo lysosome‐mediated pathway for MHC‐II‐dependent peptide loading as in (A). (D) cDC1 antigen presentation cells promote antigen cross‐presentation via clec9a‐mediated cell signaling. Endogenous antigens, such as dead or damaged cells, were recognized and ligated by clec9a, prior and during cargo endocytosis. Dimeric clec9A can be activated upon ligand binding, thereby vesicle surface SYK kinase activates and assembles active NADPH oxidase (Nox) onto the cargo loaded phagosome, rupturing phagosome membrane through free radical production. The subsequent gain of cargo accessibility by cytoplasmic proteosomes enables antigen cross‐presentation as in (B).

Historically, there has been a significant ignorance regarding how cancer‐derived antigens are processed and been used to stimulate our immune system. Among all APC cells known, there was very little known whether there were specialized cell types responsible for scavenging the corps of dead cancer cells and processing those into high quality, immune‐stimulating tumor antigens. Dendritic cells (DCs) were suspected to be a major cell type due to its critical function in mediating vaccination responses and various pathogenic resistance [182]. Although it has been discovered more than 50 years ago as a bone marrow‐derived, short‐lived heterogenous group of cells [183], the subtypes of DCs and their respective functionality have just begun to be uncovered [182]. DCs can be developed mostly from myeloid‐committed progenitor (MCPs), but also from lymphoid‐committed progenitors [184, 185]. While different DCs share a common ability to process and present antigen to initiate adoptive immune response, they differ in developmental trajectories, cell surface markers, cytokine productions and more importantly function [185]. Traditionally, using mostly in vitro generated monocytes‐derived moDC models, immunologists discovered that antigens from different sources are processed in different cellular compartments and presented by distinct classes of MHC molecules on DC surface [186, 187]. This classic paradigm distinguishes viral antigens (mostly made by infected viruses in the cytosol) as presented by MHC‐I class receptors versus scavenged antigens (coming into the cell via endocytic pathway) as presented by MHC‐II class receptors [188, 189, 190, 191] (Figure 3A). Such overall distinction makes a lot of sense since if APC presents overwhelming engulfed “self” antigens via MHC‐I class molecules, it will run into significant risk of activating “self‐reactive” CD8+ cytotoxic T cells causing undesired tissue damages, whereas such response will be favorable to clean up viral‐infected unhealthy cells, preventing further viral spread in the organism. However, this classic antigen presentation mode could not account for the immune system's capability to illicit potent CD8+ T cell responses against cancer (since cancer antigens need to be digested by APCs as “meals”) nor explain CD8+ T cell responses elicited by vaccines administrated as foreign proteins (as “meal” for APC as well). It was postulated those are due to rare “cross‐presentation” events that certain endolytic cargo vehicles are diverted with MHC‐I‐enriched endosomal recycling compartment (ERC) vesicles and trafficked toward the cell membrane directly [192], or some cargo vehicles were blocked and their membrane leaked prior to reaching lysosome, such that the ingested proteins are accessible for cytosolic proteosome digestion in DC [193, 194, 195] (Figure 3B). Although both cross‐presentation mechanisms were observed via in vitro cultured DCs, how cross‐presentation is achieved within the context of cancer‐immunity cycle was found to be a different story.

### 5.3 How Does CD4+ T Cells Provide Essential Help?

While most if not all nucleated cells express MHC‐I molecules mainly presenting peptides derived from endogenous proteins, only professional APCs (such as DCs, macrophages, and B cells) express MHC‐II molecules presenting peptides derived from exogenous proteins. Although direct cytotoxic activity for priming‐dependent cancer immunity is predominantly dependent on antigen cross‐presentation by MHC‐I molecules, MHC‐II‐restricted tumor antigens and tumor‐specific CD4+ T cells have long been recognized in many cancer patients and cancer types. While earlier works using mouse cancer models have clearly established that effective cancer eradication needs direct participation of CD4+ T cells by providing critical cytokines for CD8+ T cells [196, 197, 198, 199] and even forging direct cytotoxic functions themselves via IFN‐γ and GzmB [200, 201, 202], such observations were confirmed by numerous investigations on human TILs, where patients’ favorable clinical response is tightly linked to the enrichment of CD4+ TILs with Th1 features [33, 203]. Given the multiple aspects of reported functionality of CD4+ T cells in tumor eradication [202], it is still under intense debate whether both CD4+ and CD8+ T cells would need to recognize the same tumor‐derived antigen as a pair for their optimal cooperation. There have now accumulating evidences with animal models that this might not necessary, such that CD4+ T cells can be effective helpers with antigen specificity distinct from their cytotoxic CD8+ partners. Reported with model transgenic TCR mouse strains as the sources of ACT, CD8+ T cells against OVA peptide can coordinate effective cancer eradication together with infused CD4+ T cells against GP61, when the cancer cells carry both model antigens [204]. Such separation of antigen specificity even raises the possibility that self‐reactive CD4+ cells might be efficiently mobilized for cancer eradication. Since self‐reactive CD4+ T cells are much less likely to be deleted in the thymus by central tolerance mechanism compared with their CD8+ counterparts, they are frequently found in the periphery with good tolerance [205]. Their functionality in cancer eradication is reasonably speculated, given they were well known to be critical participants helping self‐reactive CD8+ cells causing tissue damage in the cases of autoimmune attacks [118].

There are two additional lines of overwhelming evidence suggesting an intricate involvement of self‐reactive CD4+ cells in cancer surveillance and clearance. First of all, most of the early cancer vaccine studies focused on using TAAs as immunogenic agents [206, 207]. Since TAAs are self‐antigens, they predominantly trigger CD4+T cell responses as described in the early days of cancer immunotherapy [54]. Although retrospectively, those early cancer vaccines lacked significant clinical benefits for patients, not surprisingly due to their poor stimulation of antigen‐specific CD8+ cells [208], those with sufficient CD8+ T cell boosts (while still much less comparing with typical infection disease‐related antigen triggered CD8+ T cell responses) demonstrated much improved clinical outcomes [209]. This dichotomy suggests that with the sufficient cancer relevant, self‐antigen‐specific CD4+ T cells, cancer retreat can be achieved even without an overwhelming number of corresponding cytotoxic CD8+ T cells, which was further validated by MHC‐II exclusive cancer vaccine with mouse models [210]. The other critical line of evidences come with the frequent, well‐documented observation of appearance of autoantibodies against TAAs with cancer progression, such that TAAs autoantibodies have been even well recognized as important cancer diagnostic biomarkers [211]. Since those antibody producing autoimmune B cells are known to be strictly dependent on the availability of autoantigens, together with Th2 type CD4+ Tfh cells, the rise of tumor‐specific autoantibody tier during cancer development reflects a spontaneous activation process of TAAs‐specific self‐reactive CD4+ T cells, potentially by the release of TAAs from the necrotic cancer cells [212]. Normally B cell maturation is achieved within a well‐defined three‐dimensional structure for the optimized cell–cell interaction, usually within secondary lymphoid organs (SLOs). As for the production of TAAs autoantibodies, lymphoid organ‐like structures are frequently found near tumor site within the diseased tissue (named tertiary lymphoid organs, TLOs). The presence of TLOs is a strong indicator of de novo lymphoid neo‐organogenesis, which correlated with better prognosis in many solid tumors [213]. Despite whether TLO act as a functional immune hub orchestrating immune warfare against nearby cancerous lesion remains to be rigorously addressed, TLOs are strong indicators of significant CD4+ T cell activation by the locally released cancer‐associated antigens [214, 215].

There are multiple routs that a strong cancer elicited CD4+ effector activation process might occur. First of all, live cancer cells can present their endogenous antigen with MHC‐II molecules to activate tumor‐infiltrating CD4+ T cells. Although MHC‐II expression is usually restricted to APC, they can be expressed by various cancer cells [216]. MHC‐II expression across cancer types is highly variable and context dependent [217]. Given the observation that TLOs are frequently observed some distance away from the tumor site in the same tissue, it is more likely that an antigen‐relay mechanism is employed such that CD4+ T cells are more frequently activated by “professional” APCs instead [218]. Those can happen near the tumor since there are plenty of tumor‐derived antigen available if APC cells engulfed dead cancer cells. Additionally, this can happen within SLOs after the APC cells consumed with the “meal” circulate back in the lymphatic system. How is the peripheral tolerance for the presented self‐antigens relapse under given circumstances remains largely unknown [219].

### 5.4 DCs are Specialized APCs with Many Diverse Functional Subdivisions

DCs serve as pivotal regulators of immune responses, coordinating adaptive immunity through the activation of T cells against cancers and infectious agents. These cells are categorized into two principal groups: conventional DCs (cDCs) and plasmacytoid DCs (pDCs), with cDCs further partitioned into type 1 (cDC1) and type 2 (cDC2) subsets [220, 221]. Single‐cell profiling has identified cDC2 as a heterogeneous population containing distinct DC2 and DC3 clusters [222]. DC ontogeny initiates in bone marrow from hematopoietic stem cells, with earlier models proposing a shared progenitor for cDCs and pDCs [223]. However, emerging evidences indicate lymphoid lineage derivation for most pDCs [224, 225], though human pre‐pDC precursors remain uncharacterized [225]. cDC1 specification is governed by transcription factors IRF8, ID2, and BATF3 [226, 227], exhibiting characteristic surface profiles [228]. cDC2 differentiation involves IRF4, ZEB2, and NOTCH2/KLF4 signaling [228, 229, 230]. pDC development depends on multiple factors including Flt3 ligand (Flt3L), transcription factor Spi‐B [231], and E2‐2 [232, 233], maintaining conserved phenotypes across species [234].

Functional specialization among DC subsets can best manifest through involvements in differential types of T cell‐mediated immunity. cDC1 demonstrate superior antigen cross‐presentation capabilities, critical for cytotoxic CD8+ T cell activation against tumors and intracellular pathogens [235, 236]. Moreover, recent studies reveal their capacity to prime CD4+ T cells with tumor antigens, expanding their immunoregulatory repertoire [237]. cDC2 predominantly drive CD4+ helper T cell differentiation [238]. As a subset of cDC2, DC3 subpopulations were able to induce CD4+ helper T cell responses but were absent in cancer‐free lymph nodes [239] suggesting they are less likely to induce immune responses in SLOs. Contrasting with cDCs, mature pDCs specialize in rapid type I interferon production and play crucial roles in antiviral immunity [240], but pDCs are conventionally recognized as immunosuppressive in tumor. Tumor‐derived chemokines drive the recruitment of abundant immature pDCs into tumor sites [241], while TME cytokines including VEGF, TGF‐β, and IL‐10 effectively block pDC maturation and functional activation [242, 243], resulting in their compromised IFN‐α production. Furthermore, pDCs facilitate the recruitment or differentiation of regulatory T cells (Tregs) within tumors, creating an immunosuppressive niche that supports malignant progression [244]. This functional dichotomy underscores DC heterogeneity in immune regulation, with cDC1 emerging as particularly promising therapeutic targets due to their unparalleled antigen‐presenting capacity in antitumor immunity.

### 5.5 cDC1 is the DC Subpopulation Equipped with Special Receptor and Machinery, Thus Uniquely Capable for Dead Cell Antigen Cross‐Presentation

In both human and mammals, there are three major DC types with well‐characterized cell surface receptors [182]. Among these, cDC1 subtype is the unique population expressing the receptor Clec9A (DNGR‐1), a type II CLR family (the C‐type lectin receptor) transmembrane homodimer [245, 246]. After its discovery, Clec9A was found to be the major receptor detecting dead cells [247], through its direct binding to multimeric F‐actin filament that is exposed on necrotic cell corpses [248, 249, 250]. Even though CLEC9A is dispensable for the uptake of necrotic cell material by cDC1, genetic ablation or antibody‐mediated blockade of CLEC9A diminish cross‐presentation of engulfed antigens by cDC1 and the corresponding immunogenicity of necrotic cells in vivo [247]. Since cDC1‐specific Clec9A uniquely links necrosis recognition to T cell immunity, much subsequent investigations further firmly established that cDC1 is indeed the key APC cell type responsible for mediating both cancer antigen retrieval from tumor and subsequent presentation steps to engage T cells during the full “cancer‐immunity cycle” [251]. For example, with elegant mouse genetic manipulations and cell transfer experiments, Ken Murphy's group has provided the definite evidences that xenografted tumor cannot be rejected without functional cDC1 despite the presence of all other functional APC cell types, a defect that can be rescued in full by injecting specifically in vitro differentiated cDC1 back into the tumor bearing tissue [252]. A localized rescue of antigens processing from one tumor site can also kick‐start the immune rejection against remote tumor sites with little or no cDC1 present, suggesting the steady supply of tumor antigen from one site could be sufficient to maintain functional antigen‐specific T cells deployed throughout the lymph system [252]. There has also been numerous clinical reports of “spontaneous” tumor retractions on untreated sites after patients undergo localized radiotherapy, consistent with the notion that the limited availability of righteously presented tumor antigens is likely a common bottleneck restricting the propagation of cancer‐immunity cycle [253, 254].

Besides the specific scavenger receptor, cDC1 likely processes other unique features to enable its optimal transit role in realizing “garbage in, treasure out.” It has been proposed as the specialized “cross‐presentation cell,” since cancer elimination is largely dependent on cDC1‐mediated functional maintenance of cytotoxic CD8+ T cell [255, 256]. How cDC1 cross‐presents its engulfed corpus remain poorly understood; WDFY4 (WD repeat‐ and FYVE domain‐containing protein 4) was identified as cross‐presentation‐specific factor in cDC1 and cDC2‐mediated viral and tumor immunity, such that it is not required for MHC‐II presentation of antigens but essential for ingested antigens presented by MHC‐I [257]. Wdfy4 KO mice failed to reject xenografted tumor similarly to cDC1‐deficient mice, revealing a critical role for cDC1‐mediated cross‐presentation during cancer immunity, via mobilizing CD8+ T cell in an MHC‐I‐dependent manner [258].

## 6 A Multicell Type Cancer Immunity Hub Associated with Immune Surveillance Outcome

cDC1 cells, although critical, are likely not acting alone in fueling cancer immune cycle. Recent evidences suggested a multicell type cancer immunity hub resides near the cancer site and is strongly associated with successful and persistent cancer immune cycle.

### 6.1 Immunostimulatory cDC1 Acts as a Key Organizer Forming a Tightly Interacting Clusters to Execute Cancer Immunity Cycle

Consistent with a clear functional significance of antigen‐specific CD8+ effector cells as the major “ammunition head” and cDC1 cells’ unique capability converting scavenged cancer cell debris to MHC‐I‐bound tumor antigens, there is little doubt cDC1 cells is necessary for maintaining CD8+ T cell‐dependent cancer killing. Recently, a detailed pathological analysis of both mouse cancer models and human cancer biopsies have identified that a distinct population of tumor residing cDC1 employs chemokine CXCL9/10 to recruit local CD8+ TILs. In an antigen‐dependent manner, such that immunostimulatory cDC1 acts as a hub to form tightly interacting clusters with TCF1+ stem‐like CD8+ T cells [259]. The combined actions of antigen‐dependent TCR signaling, production of T cell attractive chemokine, and T cell stimulating cytokine IL12 by cDC1 enable antigen‐dependent stem‐like T cell expansion, as well as continuous intratumoral cytotoxic CD8+ T cell differentiation [260]. However, for tumor‐associated CD4+ T cell activity, it was less clear whether cDC1 is simultaneously critical. There are many other APCs with abundant MHC‐II expression, even though cDC1, as the unique corps scavenger, would be an ideal antigen processing factory providing MHC‐II‐bound tumor‐derived neoantigens as well as self‐antigens [257].

To directly test the contribution of cDC1‐centered, antigen presentation onto MHC‐II molecules, conditional genetic deletion of either MHC‐II or CD40 in cDC1 were found with severely impaired tumor rejection, pinpointing cDC1 is the major APC type coordinating simultaneous activity for both cancer targeting CD8+ and CD4+ T cell functionalities [237]. Since removing CD40 in cDC1 cells demonstrates identical tumor rejection defects as MHC‐II‐deficient cDC1 [237], this evidence supported a long suspected hypothesis that through activating CD4+ T cells with MHC‐II presented antigen, DCs are “licensed” to maintain potent antigen presentation function when their CD40 is ligated by CD40L, which is abundant on the surface of activated CD4+ T cells [255]. In human, contact with activated CD4+T cells or with CD40L alone enables specifically human cDC1 to maintain a “helped/licensed” transcriptional and functional state ex vivo [261, 262]. Such “helped” signature correlates closely with recently identified alternative DC states from thousands of cancer patient biopsies, with favorable prognostic among diverse tumor types [261, 263, 264]. Such state is absent from circulating DCs, nor from those residing in the SLOs. Therefore, this unique state of DC from clinically favorable TME is highly likely to be exactly activated by tumor antigen‐specific CD4 T cells [261]. Detailed mouse genetic analysis found multiple downstream mechanisms that such “helped” cDC1 cells are much better equipped to promote cross‐presentation‐dependent CD8+ T cell activation [265]. First of all, the “helped” cDC1 cells might be longer‐lived due to their increased expression of Bcl‐xL, an antiapoptotic protein. The prolonged lifespan of “helped” cDC1 might be necessary for them migrating back to the tumor draining lymph node to “prime” naive T cells there [265]. At the same time, “helped” cDC1 makes much higher level of both CD70, CD80/86, and 4‐1BBL, which are ligands for CD27, CD28, and 4‐1BB, respectively, all of which are well‐known TNF receptor superfamily T cell costimulatory receptors [265], making these DC much better agonists for T cell activation. At the same time, antigen‐activated CD4+ T cells can secrete IFNβ, which in turn boost the cross‐presentation capability by cDC1 [262]. Subsequently, the ligation of “helped” cDC1 cells with antigen‐specific CD8+ T cells will likely render T cells with better potency.

It is also well known that DCs will specifically boost its expression of costimulatory ligands under the specific circumstances of innate immunity receptors activation [266, 267]. Therefore, within the SLOs, only “inflamed” DCs with presented antigen are equipped to kick off adaptive immunity response. In the mostly “sterile” TME, cDC1 will likely spare the requirement for sensing PAMP/DAMP signals there. In their place, the CD40–CD40L signaling axis and cytokine signaling between CD4+ T cell/cDC1 can fulfill similar costimulatory function at least partially [261, 262].

Traditionally, effective vaccine requires coadministration of potent adjuvants (to trigger the innate immunity sensor‐dependent activation in APC cells). Given the unique capability of cDC1 to be boosted into the “helped” state, cDC1 cells targeting vaccine might be spared with the requirement for adjuvants coadministration [268, 269]. With model antigens, prior adjuvant‐free cDC1 targeting vaccine attempt has demonstrated to be effective triggering cancer clearance with a mouse xenograft model [268]. Such vaccination scheme might mediate antigen‐dependent CD8+, CD4+ T cell activation, or both [269]. Therefore, further in‐depth dissection of whether adjuvant‐free vaccination efficacy depends on particular antigen presentation mechanism or simultaneous MHC‐I and MHC‐II‐dependent pathway will be highly informative [270, 271].

### 6.2 Cancer can Escape Immunity Surveillance by Blunting the Activity of cDC1 and Disintegrating Local Immune Cell Clusters

**FIGURE 4:**
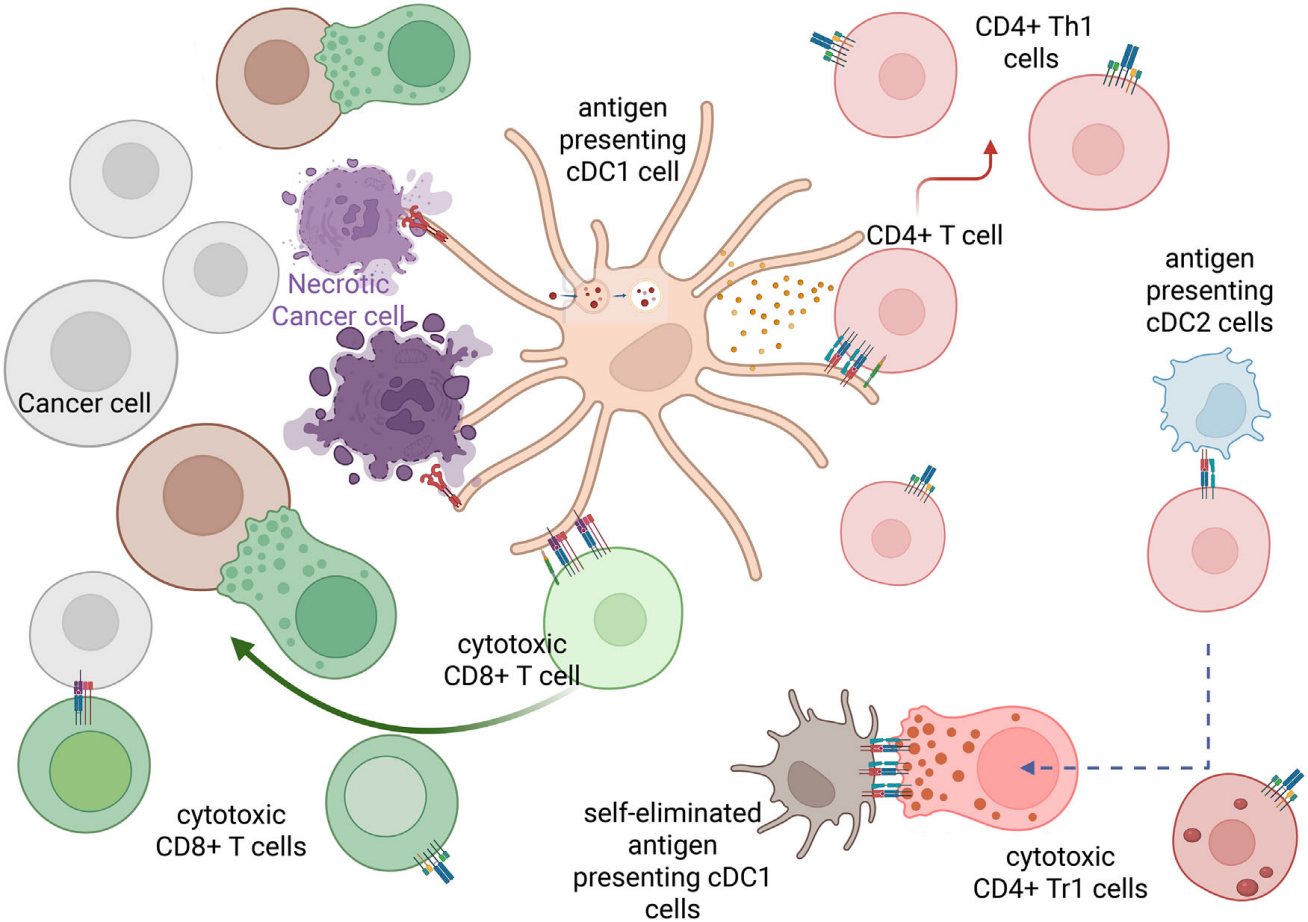
The anatomy of cancer immunity hub in the tumor microenvironment and cellular interactions with tumor antigen uptake and presentations, antigen‐dependent cellular interactions and cytokines‐dependent cellular signaling. The critical role of cDC1 in orchestrating effective, processive cancer‐immunity cycles involve at least two major effector arms of antigen‐sensing T cells. The combined actions of antigen‐dependent TCR signaling, production of T cell attractive chemokine and T cell stimulating cytokine IL‐12 by cDC1, enable antigen‐dependent stem‐like T cell expansion, as well as continuous intratumoral cytotoxic CD8+ T cell differentiation. At the same time, through activating CD4+ T cells with MHC‐II‐presented antigen, DCs are licensed to maintain potent antigen presentation function when their CD40 is ligated by CD40L, which is abundant on the surface of activated CD4+ T cells. Such three cell‐type triad has been identified in both mouse models and human cancer biopsies as a potent cancer immune hub. The supply of cancer‐specific antigen is through the unique capability of cDC1 cells to engulf and cross‐present necrotic dead cells at the tumor site. The effector cells generated from such immune hubs can effectively mobilize to kill cancer cell in an antigen‐dependent manner. Tumor‐associated antigens can be presented through cDC2 cells as well, which might be related to the activation of a subset of cytotoxic CD4+ cells (CD4+ Tr1 cells), degrading the cancer immunity hub by killing antigen presenting cDC1 cells.

From the progresses made in the past a few years, we begin to appreciate the critical role of cDC1 in orchestrating effective, processive cancer‐immunity cycles involving at least two major effector arms of antigen‐sensing T cells [237] (Figure 4). Given the limited life span of cDC1, it is thus empirical to speculate that without a steady supply of replenishing cDC1 with optimal functionality within the tumor microenvironment, the tumor‐immunity cycle may be significantly reduced/impaired [272]. Indeed, there have been numerous studies suggesting in mouse models, immunosuppressive metabolites tumor secreted such as PGE2 and IL‐6 can blunt immune surveillance by either putting local, differentiated cDC1 cells into a dysfunctional state [144], or blocking cDC1 cells development from MCPs systematically [273] (Figure 4). Moreover, in solid tumor with significant size, it has been demonstrated the severe deprivation of NO within the interior of hypoxic tumor significantly prevent tumor antigen bearing DC migrating back to tumor‐associated lymph nodes, attenuating the progression of cancer immune cycle [274]. This poorly investigated phenomenon parallels with well‐characterized T cell exhaustion as discussed in the previous section. A DC‐centered immune exhaustion phenomenon, though newly revealed, might provide another critical intervention opportunity to kick start effective cancer‐immunity cycle.

## 7 AcDC1‐Specific Targeted Cancer Vaccine Strategy to Maximize Antitumor Immune Efficacy

With the gradual elucidations on the molecular details of complex cellular interactions between live/necrotic cancer cells, cDC1, antigen‐specific CD4+, and CD8+ T cells in terms of their individual or collective functional status, it is becoming clear that a dynamic cellular immune hub has to be orchestrated near the source of cancer antigens (the tumor itself with continuous cellular turn over) for efficient immune restrains to be applied upon the tumor growth [255]. Such realization comes shortly after we understand just having the cytotoxic machinery up and running is not really enough to carry out a sustained damage to solid tumor, as numerous CAR‐T and TCR‐T therapeutic trials have not reached satisfactory clinical efficacy [275]. In the past few months, indeed, so called immune‐triad or immune hubs have been found beyond mouse cancer models. Its presence in numerous human cancer biopsy studies has clear prognosis value indicating better overall outcomes or better responses to ICI therapies [259, 260]. While the traditional CAR‐T or cancer vaccine strategies mainly focused on boosting the absolute number of cancer antigen‐specific cytotoxic cells, the absolute number of cytotoxic cells does not necessarily translate toward overcoming the rate limiting step in cancer‐immunity cycle. Therefore, it is about the right time to devise next‐generation cancer therapeutics based on facilitating fruitful constructions of cancer antigen‐centered immune cell hubs near all tumor sites.

Vaccination, as a critical immune modulatory strategy in our medical history, aims to boost the number of specific cytotoxic T effector cells or to generate long lasting antibody producing B cells, in order to prepare for the incidence of prospective pathogen invasion. Given the immunological principles we have learned about cancer immunity during the past decades, it is increasingly clear that we would be able to employ artificially designed cancer vaccines to boost both the quantity and quality of key cell types in critical shortage, fighting off cancer cells in our own body. The rest of our article will focus on discussing how to design next‐generation cancer vaccine that can significantly shift the paradigm of current cancer therapy.

### 7.1 Cancer Vaccine Should be Devised to Maximize Neoantigen‐Specific CD8+ T Cell Responses

Tradition vaccines against infectious diseases can achieve its efficacy usually by boosting the production of corresponding neutralizing antibodies. Therefore, the traditional vaccines mainly focus on stimulating MHC‐II‐dependent CD4+ T cell activity, which in turn, through antigen‐dependent CD4+ TfH cell and B cell interaction in the SLO, triggers specific B cell evolution via antibody affinity maturation and B cell differentiation. According to the canonic antigen presentation dogma, if the vaccine can be effectively taken by the DCs, its degradation products will be mainly presented by MHC‐II molecules (Figure 3A). Given adjuvant molecules taken by the same DC, the activation of innate immunity receptors will ensure the DC with foreign antigen presented can be simultaneously transformed into a mature and “stimulative” phenotype, since if its costimulatory ligands and MHC molecules expression boosted, DCs will facilitate antigen‐specific T cells within the lymph nodes reaching full activation. While this practice has worked nicely with infectious disease prevention, there is little evidence that autoantibodies generated in association with tumor development are directly involved in cancer immunity [276]. While it has been well recognized that CD4+ T cells are critically needed for elicit effective immune attacks at the tumor site, it is less clear whether the lack of cancer reactive CD4+ cells in our body could be a critical rate‐limiting bottleneck in human cancer‐immunity cycle. Recent mouse model studies also suggested that CD4+ T cells involved in “helping” the cytotoxic CD8+ cells can be of different antigen specificity [204]. Therefore, even “self‐reactive” CD4+ T cells can fulfill such critical role. Since a majority of MHC‐II‐restricted epitopes around the tumor site should be from self‐antigens processed from cell debris, the availability of a wide variety of “self‐reactive” CD4+ T cells would be capable to engage with antigen presenting cDC1. Retrospectively, over the past few decades, the overall impact of TAAs‐based vaccines on disease‐free survival, overall survival, and cancer recurrence has been minimal [277], although potent CD4+ center immune responses elicited by those vaccination regimes have been widely reported. Self‐TAA antigen‐specific TCRs are reported to have weaker structural avidity comparing with those against neoantigens [37]. Therefore, their antigen‐dependent engagement at the tumor site might be suboptimal, making the corresponding T cells more susceptible to exhaustion. Nevertheless, a tumor targeting CD4+ exclusive, neoantigen vaccination scheme have demonstrated significant efficacy with mouse cancer transplantation models, via enhancing both effector function and clonal breadth of endogenous tumor‐specific CD8+T cells [278].

CD4+ T cells can demonstrate a wide range of functional phenotypes. Tolerogenic FOXP3+ Treg cells were well known to dampen potential CD8+ T cell effector function by providing inhibitory cytokines, especially in the tumor microenvironment [279]. It remains poorly understood how antigen stimulation can be fine‐tuned to generate distinct CD4+ T cell populations. Several recently discovered tumor antigens named “inhibigens” can suppress antitumor efficacy of cancer vaccines, likely through preferentially amplifying corresponding antigen‐specific Treg cells [280]. On the other hand, there are clear indications that a portion of CD4+ T cells can acquire cytotoxic effector phenotype, which were thought originally as potential cancer killing CD4+ T cells [200]. A recent report provided contradicting functional evidence that such cytotoxic CD4+ T cells can instead eliminate antigen presenting cDC1 cells near the tumor site, demonstrating a clear suppressive function against cancer immunotherapy [281] (Figure 4). It is intriguing how such cytotoxic Tr1 CD4 T cells are primed by antigens provided by the vaccine. A clear dependence of cytotoxic Tr1 CD4 T cell‐specific priming on the dosage of antigen applied and the availability of distinct APCs was found. It was shown cytotoxic Tr1 CD4+ T cells were preferentially primed by cDC2 cells and macrophages, and if the same antigen is presented by cDC1 cells instead, “helper” CD4+ T cells were preferentially induced [281]. Nevertheless, it is still in the early days to pinpoint the general correspondence between specific antigen presentation cells, presentation mode and preferentially triggered T cell responses. However, given the evidences available, we argue a cDC1‐centered antigen delivery and processing strategy might best fulfill both arms of antitumor T cell immunity simultaneously.

Given the understanding that the majority of cancer killing activity is achieved by the canonical CD8+ effector T cells instead of cytotoxic CD4+ T cells, recent neoantigen focused cancer vaccine studies have shifted their focus to elicit strong specific CD8 T cell responses. The availability of mRNA vaccine technology enables flexible combination of multiple personalized neoantigens into a single mRNA format [282]. Such personalized vaccine is formulated with three successive steps: first of all, the patient's cancer biopsy is subjected to genome sequencing, identifying available potential neoantigens; second, based on the patient's own HLA haplotype, the respective binding of each neoantigen to the individual's HLA molecules are predicted and corresponding immunogenicity of each neoantigen is ranked. Since it is still unrealistic to reliably predict the binding and immunogenicity of peptide–MHC‐II complexes, so far most neoantigen prediction pipelines ranks these peptides based on the likelihood a potent CD8+ T cell response can be elicited [283]. Finally, the top ranked neoantigen sequences are combined and coded into a single mRNA sequence for vaccine production. In combination with ICI therapy, personalized mRNA cancer vaccines have for the first time demonstrated superior clinical outcomes in a randomized clinical trial [284—286]. These initial successes with melanoma and PDAC therefore clearly demonstrated boosting more cancer targeting neoantigen‐specific (most likely) CD8+ T cells can further tilt the balance of cancer‐immunity cycle beyond ICI therapy [287]. Although personalized cancer vaccines hold great promises, it comes with two inherent shortcomings. First of all, the requirement for individualized design and manufacturing inevitably comes with significant production time and costs, which is hard to be widely used for the general public. Second, most personalized neoantigens are from so called “passenger mutations”; therefore, the selection pressure imposed by the such immune surveillance can easily drive the propagation and evolution of cancer cells free of such passenger mutations, hence tumor evasion against therapy [288, 289, 290, 291]. Therefore, shared, driver mutation‐derived “public” neoantigens have gained considerable traction in cancer therapy [292, 293]. There have been numerous reports on the availability of such public neoantigen‐specific T cells discovered either at tumor biopsies, or from patient circulation and even from healthy individuals [68, 294, 295, 296, 297, 298]. Such accumulating evidences are arguing against the speculation that a vacuum of immunity against public neoantigens specifically allows for the high perceived occurrence of corresponding “hot‐spot” mutations on cancer driver genes among all cancer patients. In addition to the clear efficacy of ACT therapy equipped with corresponding public neoantigen‐specific TCR [294, 299, 300, 301], a number of peptide or mRNA based vaccination schemes have demonstrated promising immune potency in the scenarios of either clearing minimal residual diseases or halting advanced diseases [302, 303, 304]. A summary of ongoing clinic trails using various formats of cancer vaccines is included with this review for interested readers to dive in further (Table 1).

**TABLE 1:** Summary of ongoing clinical trials employing diverse cancer vaccine formats.

NCT no.	Clinical trial phase	Tumor type	Target	Vaccine type	Combination therapy	Study status
NCT05078866	1,2	LS	MLH1, MSH2/EPCAM, MSH6, or PMS2	Adenoviral vaccine		Active, not recruiting
NCT04990479	1	Melanoma; NSCLC	Personalized neoantigens	Adenoviral vaccine	Anti‐PD‐1	Terminated
NCT05456165	2	CRC	Personalized neoantigens	Adenoviral vaccine	Anti‐PD‐1/CTLA‐4	Withdrawn
NCT06631079	1,2	All solid tumors	Personalized neoantigens	Bacteria vaccine	Anti‐PD‐1/PD‐L1	Recruiting
NCT05631886	1	All solid tumors, lymphoma	TP53 epitope + EphA‐2	CAR–DC vaccine	Anti‐PD1/CTLA4, chemotherapy	Recruiting
NCT06522919	2	pMMR mCRC	Not available	DC vaccine		Recruiting
NCT06342908	1	DHG	H3 G34	DC vaccine		Recruiting
NCT06751940	NA	CRC	Personalized neoantigens	DC vaccine	Conventional second‐line therapy	Recruiting
NCT06751953	NA	CRC	Personalized neoantigens	DC vaccine	Conventional third‐line therapy	Recruiting
NCT01885702	1,2	CRC	Personalized neoantigens	DC vaccine		Active, not recruiting
NCT02956551	1	NSCLC	Personalized neoantigens	DC vaccine		Unknown
NCT06682117	NA	All solid tumors	Personalized neoantigens	DC vaccine		Recruiting
NCT04147078	1	GC, HCC, NSCLC, CRC	Personalized neoantigens	DC vaccine		Recruiting
NCT00923910	1,2	AML, ALL, CML, MDS, NHL	WT1	DC vaccine		Completed
NCT04078269	1	NSCLC	Personalized neoantigens	DC vaccine		Active, not recruiting
NCT04105582	1	BC	Personalized neoantigens	DC vaccine		Completed
NCT04912765	2	CRC, HCC	Personalized neoantigens	DC vaccine	Anti‐PD‐1	Recruiting
NCT04968366	1	Glioblastoma	Multiple neoantigen peptide	DC vaccine	Temozolomide	Recruiting
NCT05270720	1	OC	Personalized neoantigens	DC vaccine		Unknown
NCT05767684	1	Pancreatic cancer, HCC, BTC, CRC	Personalized neoantigens	DC vaccine	Anti‐PD‐1/VEGF	Recruiting
NCT03914768	1	DIPG or glioblastoma	Genetically modified tumor cells	DC vaccine	Anti‐VEGF	Unknown
NCT05631899	1	All solid tumors	KRAS	DC vaccine	EphA‐2 CAR, anti‐PD‐1/CTLA4	Recruiting
NCT06435351	Early1	BC	Personalized neoantigens	DC vaccine		Recruiting
NCT06329908	Early1	Lung cancer	Unknown neoantigen	DC vaccine	ICIs	Recruiting
NCT03871205	1	NSCLC, SCLC	Personalized neoantigens	DC vaccine		Unknown
NCT03674073	1	HCC	Personalized neoantigens	DC vaccine	Microwave ablation	Unknown
NCT06675201	2	ESCC	Personalized neoantigens	DC vaccine	ICIs	Recruiting
NCT05317325	1	ESCC	oxidized autologous tumor lysate	DC vaccine		Unknown
NCT05749627	NA	All solid tumors	Personalized neoantigens	DC vaccine		Recruiting
NCT06253234	1	Glioma	Personalized neoantigens	DC Vaccine		Recruiting
NCT06751849	2	Advanced NSCLC	Personalized neoantigens	DC Vaccine	Anti‐PD1, radiotherapy	Recruiting
NCT06752057	NA	Advanced NSCLC	Personalized neoantigens	DC vaccine	Anti‐PD1, radiotherapy	Recruiting
NCT03870113	1	CIN	HPV16/​18 E6/​E7	DC Vaccine		Unknown
NCT05235607	1	Carcinoma, melanoma, BLCA, CRC	Personalized neoantigens	DC vaccine and DC–CIK		Unknown
NCT05020119	1	All solid tumors	Personalized neoantigens	DC–CIK cells		Unknown
NCT03205930	1,2	NSCLC	Personalized neoantigens and shared TAAs	DC–CIK cells		Unknown
NCT04015700	1	Glioblastoma	Personalized neoantigens	DNA vaccine	IL12	Active, not recruiting
NCT04397003	2	SCLC	Personalized neoantigens	DNA vaccine	Anti‐PD‐L1	Recruiting
NCT05354323	1	NSCLC, melanoma, RCC, HNSCC	Personalized neoantigens	DNA vaccine	Anti‐PD‐1/PD‐L1	Recruiting
NCT05743595	1	Glioblastoma	Personalized neoantigens	DNA vaccine	Anti‐PD‐1	Recruiting
NCT03532217	1	Prostate	Shared neoantigen	DNA vaccine	Anti‐PD‐1/CTLA‐4	Completed
NCT03199040	1	BC	Personalized neoantigens	DNA vaccine	Anti‐PD‐L1	Terminated
NCT03598816	2	RCC	Not available	DNA vaccine	Anti‐PD‐1/CTLA‐4	Withdrawn
NCT04251117	1,2	HCC	Personalized neoantigens	DNA vaccine	IL‐12, anti‐PD‐1	ACTIVE_NOT_Recruiting
NCT04001413	2	OSCC	HPV‐16 E6/E7	DNA vaccine	Anti‐PD‐L1	Withdrawn
NCT03914872	NA	All solid tumors	Not available	DNA vaccine		NO_LONGER_AVAILABLE
NCT03988283	1	Brain tumor	Personalized neoantigens	DNA vaccine		Recruiting
NCT03122106	1	Pancreatic cancer	Personalized neoantigens and MSLN epitope	DNA vaccine		Terminated
NCT06577532	Early1	Pancreatic cancer	KRAS	mRNA vaccine	Anti‐PD‐1	Recruiting
NCT06156267	Early1	Pancreatic cancer	Personalized neoantigens	mRNA vaccine	Anti‐PD‐L1	Not yet recruiting
NCT06685653	Early1	NSCLC	Personalized neoantigens	mRNA vaccine	Anti‐PD‐L1	Not yet recruiting
NCT06913218	1	PDAC	Personalized neoantigens	mRNA vaccine	mFOLFIRINOX	Not yet recruiting
NCT04267237	2	NSCLC	Personalized neoantigens	mRNA vaccine	Anti‐PD‐L1	Withdrawn
NCT06497010	Early1	All solid tumors	Personalized neoantigens	mRNA vaccine	Anti‐PD‐1	Recruiting
NCT05158621	NA	CRC	Personalized neoantigens	mRNA vaccine	FOLFOX or CAPEOX, anti‐VEGF	Completed
NCT03794128	Observational	NSCLC; CRC; GEA; UC; PDAC	Personalized neoantigens; shared neoantigen	mRNA vaccine		Completed
NCT05886439	1	NSCLC, SCLC	Personalized neoantigens	mRNA vaccine	Anti‐PD‐1/PD‐L1	Recruiting
NCT06496373	Early1	Pancreatic cancer	Personalized neoantigens	mRNA vaccine	Anti‐PD‐1	Recruiting
NCT04998474	2	NSCLC	Personalized neoantigens	mRNA vaccine	Anti‐PD‐1	Unknown
NCT06141369	NA	ACC; MTC; TNC; PNT	Personalized neoantigens	mRNA vaccine		Recruiting
NCT03897881	2	Melanoma	Personalized neoantigens	mRNA vaccine	Anti‐PD‐1	Recruiting
NCT03908671	NA	ESCC	Personalized neoantigens	mRNA vaccine		Recruiting
NCT04161755	1	Pancreatic cancer	Personalized neoantigens	mRNA vaccine	Anti‐PD‐L1, mFOLFIRINOX	Active, not recruiting
NCT05192460	1	GC, ESCC, HCC	Personalized neoantigens	mRNA vaccine	Anti‐PD‐1/PD‐L1	Recruiting
NCT05198752	1	All solid tumors	Personalized neoantigens	mRNA vaccine		Recruiting
NCT05359354	1	All solid tumors	Personalized neoantigens	mRNA vaccine	Anti‐PD‐1	Recruiting
NCT05579275	1	All solid tumors	Shared neoantigen	mRNA vaccine	Anti‐PD‐1	Recruiting
NCT05916248	1	All solid tumors	Personalized neoantigens	mRNA vaccine	Anti‐PD‐1	Recruiting
NCT05916261	1	Pancreatic	Personalized neoantigens	mRNA vaccine	Anti‐PD‐1	Recruiting
NCT05940181	1	All solid tumors	Personalized neoantigens	mRNA vaccine	Anti‐PD‐1	Recruiting
NCT05981066	1	HCC	Shared neoantigens	mRNA vaccine		Recruiting
NCT06019702	1	Digestive tract cancer	Personalized neoantigens	mRNA vaccine		Recruiting
NCT06026774	1	Digestive tract cancer	Personalized neoantigens	mRNA vaccine	Standard adjuvant therapy	Recruiting
NCT06026800	1	Digestive tract cancer	Personalized neoantigens	mRNA vaccine	First‐line treatment	Recruiting
NCT06735508	Early1	NSCLC	Personalized neoantigens	mRNA vaccine	Anti‐PD‐L1	Not yet recruiting
NCT05949775	NA	All solid tumors	Personalized neoantigens	mRNA vaccine	Anti‐PD‐1	Not yet recruiting
NCT05761717	NA	HCC	Personalized neoantigens	mRNA vaccine	Anti‐PD‐1	Not yet recruiting
NCT06195384	1	All solid tumors	Personalized neoantigens	mRNA vaccine	Anti‐PD1/PDL1/CTLA4	Recruiting
NCT05227378	NA	Gastric cancer	Personalized neoantigens	mRNA vaccine	Anti‐PD1/PDL1	Not yet recruiting
NCT03468244	NA	ESCC; GEA; PADC; CRCA	Personalized neoantigens	mRNA vaccine		Unknown
NCT06980155	Early1	AML	Personalized neoantigens	mRNA vaccine	Anti‐PD1	Recruiting
NCT06326736	Early1	Pancreatic cancer	Personalized neoantigens	mRNA vaccine	Anti‐PD1, gemcitabine + abraxane	Recruiting
NCT03639714	1,2	NSCLC, MSS CRC, GEA, UC	Personalized neoantigens	mRNA vaccine	Anti‐PD1/CTLA4	Completed
NCT03953235	1,2	MSS CRC, NSCLC, PDAC, selected solid tumors	Personalized neoantigens	mRNA vaccine	Anti‐PD1/CTLA4	Completed
NCT05141721	2,3	CRC	Personalized neoantigens	mRNA vaccine	Anti‐PDL1/CTLA4/VEGF	Active, not recruiting
NCT04145232	NA	NSCLC	Personalized neoantigens	Not Available	Anti‐PD1/PDL1/CTLA4	Unknown
NCT06344156	1	Pancreatic cancer	Not available	Not available	Anti‐PD1 and chemotherapy	Recruiting
NCT03631043	Early1	SPCM	Personalized neoantigens	Peptide vaccine	Lenalidomide	ACTIVE_NOT_Recruiting
NCT06751966	NA	CRC	Personalized neoantigens	Peptide vaccine	Conventional third‐line therapy	Recruiting
NCT06751914	NA	CRC	Personalized neoantigens	Peptide vaccine	Conventional second‐line therapy	Recruiting
NCT02600949	1	Metastatic CRCA; metastatic PDAC; CRC	Personalized neoantigens	Peptide vaccine	Imiquiomod, anti‐PD‐1/CD40	Recruiting
NCT04397926	1	NSCLC	Personalized neoantigens	Peptide vaccine		Unknown
NCT03662815	1	All solid tumors	Personalized neoantigens	Peptide vaccine	GM‐CSF	Unknown
NCT03956056	1	Pancreatic cancer	Personalized neoantigens and MSLN epitope	Peptide vaccine		Terminated
NCT06195293	1	All solid tumors	Personalized neoantigens	Peptide vaccine	Anti‐PD1/PDL1/CTLA4	Recruiting
NCT06341907	2,3	OC	Personalized neoantigens	Peptide vaccine		Recruiting
NCT04487093	1	NSCLC	Personalized neoantigens	Peptide vaccine	EGFR‐TKI, antiangioge	Unknown
NCT06406816	Early1	ICC	Personalized neoantigens	Peptide vaccine	Capecitabine	Recruiting
NCT04799431	1	Pancreatic cancer, CRC	Personalized neoantigens	Peptide vaccine	Anti‐PD‐1	Withdrawn
NCT03597282	1	Melanoma	Personalized neoantigens	Peptide vaccine	APX005M, anti‐PD1/CTLA4	Terminated
NCT03422094	1	Glioblastoma	Personalized neoantigens	Peptide vaccine	Anti‐PD1/CTLA4	Terminated
NCT04024878	1	OC	Personalized neoantigens	Peptide vaccine	Anti‐PD1	ACTIVE_NOT_Recruiting
NCT06631092	1,2	TNBC	Personalized neoantigens	Peptide vaccine	Anti‐PD1	Recruiting
NCT04266730	1	SCLC, NSCLC, HNSCC	Personalized neoantigens	Peptide vaccine	Anti‐PD1	Not yet recruiting
NCT03166254	1	NSCLC, SCLC	Personalized neoantigens	Peptide vaccine	Anti‐PD1/PDL1	Withdrawn
NCT01970358	1	Melanoma	Personalized neoantigens	Peptide vaccine		Completed
NCT02287428	1	Glioblastoma	Personalized neoantigens	Peptide vaccine	Anti‐PD1, temozolomide	Recruiting
NCT02897765	1	Melanoma, NSCLC, UC	Personalized neoantigens	Peptide vaccine	Anti‐PD1, adjuvant therapy	Completed
NCT02950766	1	RCC	Personalized neoantigens	Peptide vaccine	Anti‐CTLA4	Active, not recruiting
NCT03219450	1	CLL	Personalized neoantigens	Peptide vaccine	Anti‐PD1, Cyclophosphamide	Recruiting
NCT03359239	1	UC	Personalized neoantigens	Peptide vaccine	Anti‐PDL1	Completed
NCT03361852	1	FL	Personalized neoantigens	Peptide vaccine	Anti‐PD1/CD20	Active, not recruiting
NCT03380871	1	NSCLC	Personalized neoantigens	Peptide vaccine	Anti‐PD1, chemotherapy	Completed
NCT03558945	1	Pancreatic cancer	Personalized neoantigens	Peptide vaccine		Recruiting
NCT03606967	2	BC	Personalized neoantigens	Peptide vaccine		Recruiting
NCT03645148	1	Pancreatic cancer	Personalized neoantigens	Peptide vaccine	GM‐CSF	Completed
NCT03929029	1	Pancreatic cancer	Personalized neoantigens	Peptide vaccine	Anti‐PD1/CTLA4, montanide	Recruiting
NCT04117087	1	Pancreatic cancer, MSS CRC	KRAS peptide	Peptide vaccine	Anti‐PD1/CTLA4, montanide	Recruiting
NCT04248569	1	HCC	DNAJB1–PRKACA fusion peptide	Peptide vaccine	Anti‐PD1/CTLA4, montanide	Recruiting
NCT04364230	1,2	Melanoma	BRAF585‐614–V600E	Peptide vaccine	Anti‐CD40	Active, not recruiting
NCT04509167	1	All solid tumors	Personalized neoantigens	Peptide vaccine		Completed
NCT04749641	1	DIPG	H3.3–K27M	Peptide vaccine		Recruiting
NCT04810910	1	Pancreatic cancer	Personalized neoantigens	Peptide vaccine	GM‐CSF	Recruiting
NCT04864379	1	All solid tumors	Personalized neoantigens	Peptide vaccine	GM‐CSF, anti‐PD1	Recruiting
NCT04930783	1	Melanoma	Personalized neoantigens	Peptide vaccine	Anti‐PD1, CDX301	Recruiting
NCT04943848	1	Glioma	HSP	Peptide vaccine	Anti‐PD1/CTLA4	Recruiting
NCT05013216	1	Pancreatic	KRAS	Peptide vaccine	Poly‐ICLC	Recruiting
NCT05098210	1	Melanoma	Personalized neoantigens	Peptide vaccine	Anti‐PD1, poly ICLC	Recruiting
NCT05111353	1	Pancreatic	Personalized neoantigens	Peptide vaccine	Poly‐ICLC	Recruiting
NCT05238558	1	All solid tumors	FMPV‐1	Peptide vaccine	GM‐CSF	Completed
NCT05269381	1	All solid tumors	Personalized neoantigens	Peptide vaccine	Anti‐PD1, cyclophosphamide	Recruiting
NCT05307835	1	ESCC	Personalized neoantigens	Peptide vaccine	GM‐CSF	Recruiting
NCT05475106	1	All solid tumors	Personalized neoantigens	Peptide vaccine	Sargramostim	Recruiting
NCT05641545	1	RCC	Personalized neoantigens	Peptide vaccine	GM‐CSF and imiquimod	Recruiting
NCT05741242	1,2	All solid tumors	Personalized neoantigens	Peptide vaccine		Enrolling by invitation
NCT06095934	NA	NSCLC	Not available	Peptide vaccine	Anti‐PD1	Recruiting
NCT04072900	1	Melanoma	Personalized neoantigens	Peptide vaccine	Anti‐PD1	Unknown
NCT06614140	1,2	All solid tumors	Personalized neoantigens	Peptide vaccine	Poly‐ICLC and ICIs	Active, not recruiting
NCT03068832	1	Pediatric brain tumor	Personalized neoantigens	Peptide vaccine	Poly‐ICLC	Withdrawn
NCT02510950	1	Glioblastoma, astrocytoma	Personalized neoantigens	Peptide vaccine	Poly‐ICLC, temozolomide	Terminated
NCT06963697	NA	Cancer	Personalized neoantigens	Peptide vaccine		Available
NCT04943718	1	Malignant glioma; recurrent glioma	Personalized neoantigens	Peptide vaccine	Anti‐PD1	Unknown
NCT06314087	2	Advanced tumors	Personalized neoantigens	Peptide vaccine		Recruiting
NCT06751901	2	Advanced NSCLC	Personalized neoantigens	Peptide vaccine	Conventional treatment including radiotherapy	Recruiting
NCT06752044	NA	Advanced NSCLC	Personalized neoantigens	Peptide vaccine	Anti‐PD1, radiotherapy	Recruiting
NCT06529822	1	Muscle‐invasive bladder carcinoma	Personalized neoantigens	Peptide vaccine	Poly‐ICLC	Recruiting
NCT04087252	1	Cancer	Personalized neoantigens	Not available		Unknown
NCT02992977	1	Advanced cancer	Personalized neoantigens	Peptide vaccine + HSP70		Terminated
NCT03673020	1	All solid tumors	Personalized neoantigens	Peptide vaccine + HSP70		Completed
NCT05153304	1,2	Cancer of gastrointestinal tract	Personalized neoantigens	Not available	Anti‐PD1	Withdrawn
NCT03568058	1	All solid tumors	Personalized neoantigens	Not available	Anti‐PD1	Active, not recruiting
NCT03807102	1,2	Lung cancer	Personalized neoantigens	Not available		Not yet recruiting
NCT05685004	2,3	Glioblastoma	Autologous cancer cell vaccines	Not available		Suspended
NCT05356312	NA	Glioma	Personalized neoantigens	Not available		Available
NCT06353646	NA	Pancreatic cancer	Personalized neoantigens	Not available	Anti‐PD1	Recruiting
NCT05444530	1	MPN	CALR, JAK2V617F	Virus vaccine	Anti‐CTLA4	Recruiting
NCT03552718	1	All solid tumors	Personalized neoantigens	Yeast vaccine		Active, not recruiting

Abbreviations: ACC, adrenal cortical carcinoma; ALL, acute lymphoblastic leukemia; AML, acute myeloid leukemia; APC, advanced pancreatic cancer; BC, breast cancer; BLCA, bladder cancer  ; BTC, biliary tract cancer; CIN, cervical intraepithelial neoplasia; CLL, chronic lymphocytic leukemia; CML, chronic myeloid leukemia; CRC, colorectal cancer  ; CRCA, colorectal adenocarcinoma; DHG, diffuse hemispheric glioma; DIPG, diffuse intrinsic pontine glioma; ESCC, esophageal squamous cell carcinoma; FC, follicular lymphoma; GBM, glioblastoma multiforme; GC, gastric cancer; GEA, gastroesophageal adenocarcinoma; GEJ, gastroesophageal junction; HCC, hepatocellular carcinoma; HNSCC, head and neck squamous cell carcinoma; ICC, intrahepatic cholangiocarcinoma; LC, lung cancer; LS, lynch syndrome; MDS, myelodysplastic syndromes; MMR, mismatch repair; MPN, myeloproliferative neoplasm; MSI, microsatellite Instability; MSS CRC, microsatellite stable colorectal cancer; MTC, medullary thyroid cancer; NGS, next‐generation sequencing; NHL, non‐Hodgkin lymphoma; NSCLC, non‐small cell lung cancer; OC, ovarian cancer; OSCC, oropharyngeal squamous cell carcinoma; PDAC, pancreatic ductal adenocarcinoma; pMMR, mismatch‐repair‐proficient; PNT, pancreatic neuroendocrine tumor; RCC, renal cell carcinoma; SCLC, small cell lung cancer; SPCM, smoldering plasma cell myeloma; TNBC, triple‐negative breast cancer; TNC, thymic neuroendocrine carcinoma; UC, urothelial carcinoma.

*Data sources*: ClinicalTrials.gov.

### 7.2 Stimulating Neoantigen‐Specific CD8+ T Cell Responses Requires Effective Antigen Cross‐Presentation

The challenge to elicit potent CD8+ T cell responses is unique for cancer vaccines design, since the pivotal contribution of amplified antigen‐specific CD8+ T cells was less critical for traditional vaccines. If supplied as in a protein‐based format, neoantigen vaccine will most likely been processed and presented in an MHC‐II‐dependent manner, since most DCs do not spontaneously “cross‐present” exogenous antigens. Therefore, effective cancer vaccine requires cross‐presentation of antigens by design. There have been numerous attempts to devise novel adjuvants favoring the cross‐presentation capability by acting as “proton‐sinks” once antigens engulfed in endosome, triggering subsequent swelling and rupture of the endosomal membranes before the endosomes reach lysosome [305, 306, 307, 308, 309] (Figure 3B). However, most “proton‐sinks” compounds have unavoidable cytotoxicity, such that its safety has not been rigorously evaluated with animal or preclinic studies [310]. Novel nanomaterial‐based adjuvants, on the other hand, might provide better safety profiles, with the additional benefits of boosting DCs maturation and migration capability [311].

While traditional adjuvant is highly desirable for vaccines to reach satisfactory efficacy, the relationship between adjuvant and antigen cross‐presentation is less clear [312]. Although it was speculated that protein‐based antigen processing and presentation and DC activation are separated processes, there are detailed cell biology studies suggesting the fate of endocytic cargo can be tightly coupled with whether the cargo itself can elicit potent innate immunity signaling. For example, Blander lab has demonstrated that ligating TLR4 agonist LPS with the phagosome cargo will favor the particular phagosomes carrying activated TLR/Myd88 complex to fuse with MHC‐I enriched ERC compartment, thereby short‐cutting the antigen‐derived peptides back to the plasma membrane in an MHC‐I‐bound format [192] (Figure 3C). While this mechanism was learned using mo‐DC with in vitro cell culture system, it not yet clear whether conjugating any TLR agonist with protein cargo will facilitate efficient antigen cross‐presentation in all DC subtypes.

Besides Clec9a‐equipped cDC1, it is hard to pinpoint exactly whether and how much antigen cross‐presentation is carried out by other APC cell types in vivo [313, 314]. Given the recent identification of cross‐presentation‐specific factor WDFY4 in mice (without which a number of viral‐related antigens cannot be processed to trigger cytotoxic T cell response), it is likely that a majority of antigen cross‐presentation in vivo is cDC1 cells dependent since WDFY4 is mainly expressed in cDC1 cells, and to a less degree in cDC2 cells [257, 258]. How WDFY4 exactly mediates cross‐presentation is poorly understood. However, it has been proposed that clec9a, after mediating cargo phagocytosis, is directly involved in preparing its captured prey for cross‐presentation [256]. Dimeric clec9A can be activated upon ligand binding, thereby vesicle‐associated SYK assembles active NADPH oxidase onto the cargo loaded phagosome, causing phagosome membrane rupture and enclosed antigen cross‐presentation [245] (Figure 3D). On the other hand, lectin family member Siglec‐G associated with the phagosome can suppress cross‐presentation by recruiting SHP‐1 phosphatase, antagonizing SYK/Nox‐dependent membrane leakage [315]. Since Siglec‐G binds to sialic acids terminated glycans, many cancer cells specifically utilized glycosylation modification with sialic acid on their cell surface, potentially to evade efficient antigen cross‐presentation once their debris engulfed by cDC1 [316, 317]. Although antigen cross‐presentation coupled with scavenging receptor engagement on cDC1 cells surface is appealing [268, 318, 319, 320, 321], this model likely over‐simplifies how cross‐presentation is regulated delicately. For example, whether the role of innate immunity receptor ligation/activation is conserved in promoting cargo cross‐presentation in cDC1 cell is not clear. In addition, known cross‐presentation regulator WDYF4 remains a critical piece to be fit in the sophisticated puzzle.

### 7.3 Effective Cancer Vaccine Should Target cDC1 Cells, Providing Antigens to be Presented While Fully Engaging DC Activity

**FIGURE 5:**
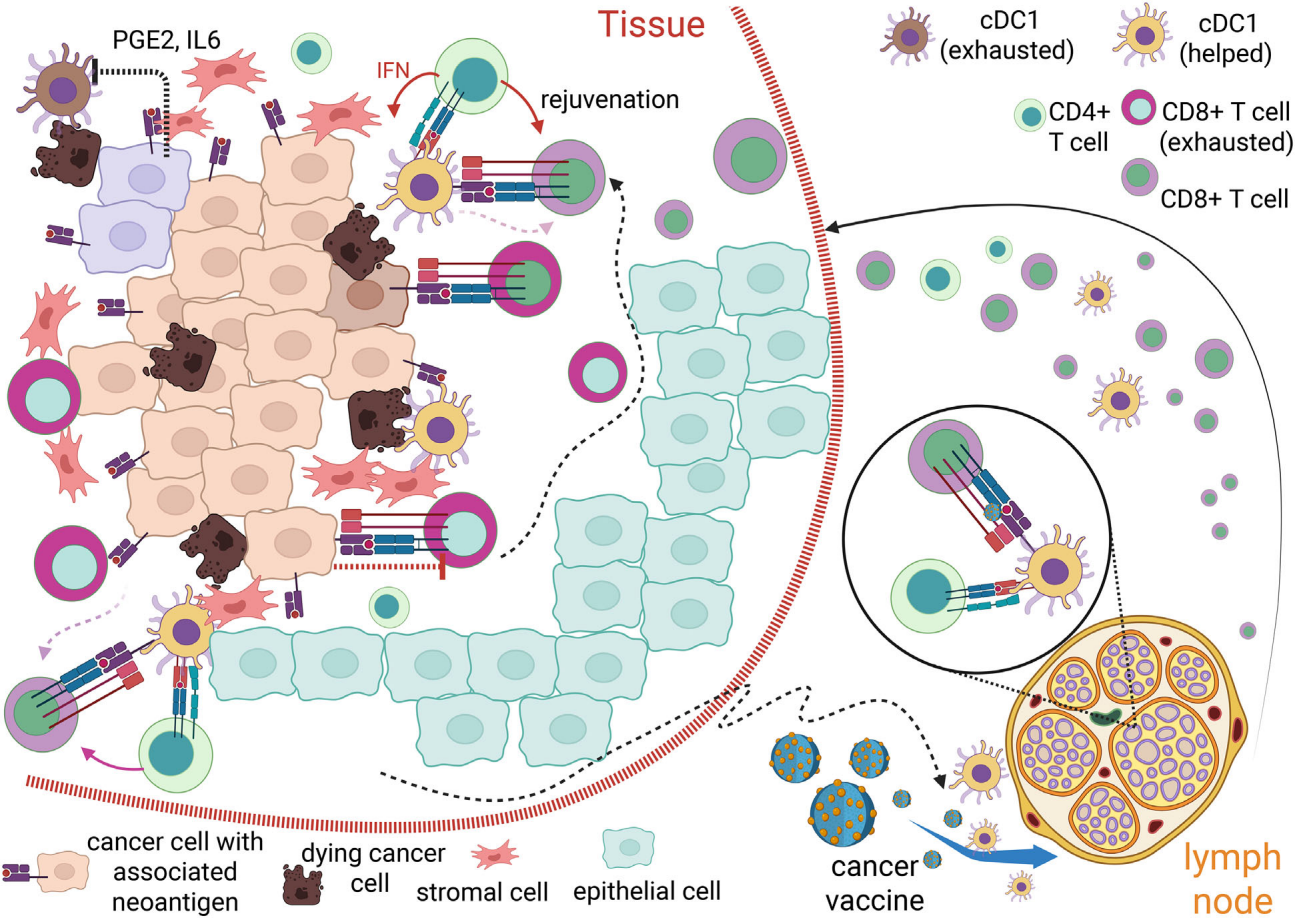
Cancer vaccines boost the perpetual cancer immunity cycle. Efficient cancer vaccine can be targeted to cDC1 antigen‐presenting cells in the lymph node, via activating both two major effector arms of antigen‐sensing T cells. Those expanded antigen‐specific T cells migrate into the tumor bearing tissue, forming three cell‐type triad immune hub near the tumor site. In the tumor microenvironment, cDC1s are responsible of scavenging dead cancer cell corpse and subsequent antigen processing, while soluble cytokines factors made by CD4+ helper cells such as IFN‐γ enhance cDC1 antigen presentation and activation. Soluble cytokines also rejuvenate previously exhausted CD8+ Tex progenitor cells. In addition to causing T cell exhaustion during prolonged antigen exposure, cancer cells can antagonize cancer immune surveillance by secreting PGE2 and IL‐6, blocking cDC1 function and differentiation. Therefore, cancer vaccines can work best in combo with other therapeutic modules including cancer radio/chemotherapies, T cell immune checkpoint inhibitors, and novel therapeutics relieving DCs from function suppression.

Given the key role of cDC1 in antigen cross‐presentation, as well as at the hub for cancer immunity near the tumor site [322], it is important to understand how innate immunity receptors and scavenging receptors activation collectively mediate cell signaling coupled cross‐presentation regulation in cDC1. Whether any of these signaling events is directly coupled with cDC1 cell maturation and gain of migratory potential as well as T cell activation potential remain a critical puzzle to resolve. Currently, nanoparticle carriers [322], self‐assembling platforms [323], and albumin‐binding vehicles [324] stand out as predominant strategies to facilitate the delivery of vaccine constituents to lymph nodes, ensuring that antigens make efficacious contact with DCs and enhance their capacity to capture and process these antigens. Moreover, these delivery systems can concurrently incorporate agents that promote DC maturation and augment T‐cell stimulation, thereby priming a more potent immunological response. For example, including STING agonists in the nano particle formatted vaccine has been found to promote cDC1 activity via DC autonomous IFN secretion [325, 326]. Since DCs themselves are short‐lived in vivo, DC vaccines are unlikely to have long lasting direct efficacy. However, there could be multiple ways beyond cancer vaccines to boost both the number and activities of cDC1 both in the circulation and in the tissues under surveillance [327]. Administering Flt3 ligand (FLT3L) and granulocyte‐macrophage colony‐stimulating factor (GM‐CSF) systemically or locally within the tumor microenvironment serves as a powerful strategy to bolster this specific DC populations [328]. Under inflammatory condition, DCs are innately wired to traffic towards the chemokines released by the inflamed site [329]. Although most solid tumor are immune‐cold, therapeutics such as oncolytic viruses can efficiently trigger local inflammatory responses, facilitating long lasting functional DC infiltration and maturation [217, 330]. Furthermore, recent investigation has attempted to reprogram cancer cells into DCs like APC directly by delivering a reprogramming factors cocktail [331]. Such programming effectively reshaped the tumor microenvironment and triggered potent‐specific T cell immune responses and cancer regression in mouse models [331]. The harvest and presentation of cancer‐associated antigen via those cDC1 can efficiently amplify the available adaptive immune responses. NK cells recruit cDC1 into the tumor via chemokines CCL5 and XCL1, promoting cancer immune control [143], whereas PGE2 produced by tumor leads to immune evasion by impairing NK cell viability and decreasing both NK chemokine production and cDC1 chemokine receptor expression [144] (Figure 5). To antagonize immune suppressive tumor microenvironment, tumor‐associated in situ hydrogel vaccines (FP/NCUM‐Gel) have been devised to form a local cDC1 favoring niche enhancing cancer vaccines effectiveness [332]. Given the previously learned lessons from both animal models and clinical observations, the lack of cDC1, or “helped/licensed” cDC1 as the local acting immune cell hubs are now regarded as a frequent pitfall why patients are refractory to cancer immune therapy [333]. In contrast to infection induced Adaptive immunity response where the T cell effector differentiation can be triggered quickly in the LN with the simultaneous availability of activation signals, recent mechanistic studies demonstrated a different scenario for antitumor immunity. T cell effector differentiation within TME is strictly a two‐step process dependent on local availability of both the antigen and costimulation signal [129]. Although there are still ongoing debates on the critical DC type fulfilling the critical roles of delivering those activation signals within TME [129, 260], the DC progenitors ectopically expressing two immunostimulatory cytokines can suppressed tumor growth via expanding and activating cDC1 population, without the need for antigen loading or host myeloablative conditioning [334]. All of those above strategies can be complemented with efficient cDC1‐centered cancer vaccines, providing highly effective innate and adaptive immunity arsenals orchestrating long lasting battles at the cancer site.

## 8 Conclusion and Prospects

Cancer immunotherapy is causing a more and more active seismic shift in our fights against malignant cells that went rogue, bringing closely collaborative clinicians, basic scientists, and biomedical engineers together. Therefore, with the rapid advances on the understanding of how our own immune system (with distinct critical innate immune cells, antigen‐specific adoptive immune cells and bystander cells in the evolving tumor microenvironment) can orchestrate a persistent, effective battle with growing cancer cells will definitively bring upon more and more innovative and effective cancer therapeutics for each individual patient.

We also envision the effective cancer immunotherapy should be a combination of personalized strategies coping with various cancer evasion mechanisms. Cancer cells can actively suppress the T effector functions and accelerate T cell exhaustion by engaging with simultaneous checkpoint receptors and TCR signaling, either directly or through suppressive APCs. This evasion mechanisms can be at least partially reversed by various ICI therapeutic antibodies. In addition, cancer cells can sequester essential nutrients, generate potent immunosuppressive metabolites, or even “sugar coat” their own cell surface which not only target effector T cells but also critical DC types within local TME. The collapse of active local immune hubs can cool off effective cancer immune surveillance and turn the tumor into an immune “cold” state refractory to standard ICI therapies. A major challenge for cancer immunotherapy is to devise safe, effective and persistent interventions to reverse an “immune cold” tumor back into one engaged with active cancer‐immunity cycles. Among many current alternative strategies such as ACT, oncolytic viral therapies, we envision next‐generation cancer vaccines holds special promises to fulfill this critical gap.

Importantly, although this battle is fought on the basis of harvesting the necrotic cancer cells as a major source of relevant antigens to build up antitumor immunity, the lack of effective neoantigen elicited, functional immune effector cells usually can be a major rate limiting step in maintaining efficient cancer‐immunity cycle. As vaccines traditionally have been battle‐proven against many devastating infectious diseases by effectively boosting the number of antigen‐specific effector cells in circulation, cancer vaccines, especially with early but promising indications of efficacy by individualized mRNA‐based cancer vaccines, have brought us high hopes that vaccination can efficiently protect us against cancer by providing a steady supply of tumor antigen‐specific immune effector cells. We argue that smarter next‐generation cancer vaccines will optimize not only personalized antigen combinations, but also precision delivery in vivo to the most ideal APCs. Innovative approaches should also be taking account regarding boosting of activities of DCs not only after uptaking such vaccines from circulation but also maintaining their optimal functionality even within a suppressive TME. Such precision medicine practice will enable a purposeful display of the right quantities of different antigens on specific MHC molecules at functional APCs equipped with fully engaged costimulatory signals at both SLOs and within the cancer immunity hub. Such exciting advances will not only bring realistic hope saving more lives from cancer, but also advance greatly our understanding of immunity holistically.

## Author Contributions

YP coordinated the overall structure, wrote most of the paper, designed all the figures: JS, ZQ, JG summerized the current cancer vaccines, managed the citatations, and compiled the table; WJ refined the figure graphics; LZ, XS, JV, HY, XL, CL, SG, HW, YZ, BZ, WG wrote part of section 2‐6. YP, JW, YL, JS, QZ, YY, FG contributed to the revisions. All authors have read and approved the final manuscript.

## Ethics Statement

The authors have nothing to report.

## Conflicts of Interest

The authors declare no conflicts of interest.

## Data Availability

The data that support the findings of this study are available from the corresponding author upon reasonable request.

## References

[1] C. A. Janeway Jr , P. Travers , M. Walport , et al., Immunobiology. 5th ed. (Garland Science, 2001).

[2] D. S. Pisetsky , “Pathogenesis of Autoimmune Disease,” Nature Reviews Nephrology 19, no. 8 (2023): 509‐524. 37165096 10.1038/s41581-023-00720-1 PMC10171171

[3] P. Ehrlich , Studies in Immunity (J. Wiley & sons, 1910).

[4] H. Takeshima , T. Ushijima , “Accumulation of Genetic and Epigenetic Alterations in Normal Cells and Cancer Risk,” NPJ Precision Oncology 3 (2019): 7. 30854468 10.1038/s41698-019-0079-0 PMC6403339

[5] F. M. Burnet , “The Concept of Immunological Surveillance,” Progress in Experimental Tumor Research 13 (1970): 1‐27. 4921480 10.1159/000386035

[6] T. Fan , M. Zhang , J. Yang , Z. Zhu , W. Cao , C. Dong , “Therapeutic Cancer Vaccines: Advancements, Challenges, and Prospects,” Signal Transduction and Targeted Therapy 8, no. 1 (2023): 450. 38086815 10.1038/s41392-023-01674-3 PMC10716479

[7] P. Berraondo , M. F. Sanmamed , M. C. Ochoa , et al., “Cytokines in Clinical Cancer Immunotherapy,” British Journal of Cancer 120, no. 1 (2019): 6‐15. 30413827 10.1038/s41416-018-0328-y PMC6325155

[8] A. D. Waldman , J. M. Fritz , M. J. Lenardo , “A Guide to Cancer Immunotherapy: From T Cell Basic Science to Clinical Practice,” Nature Reviews Immunology 20, no. 11 (2020): 651‐668. 10.1038/s41577-020-0306-5 PMC7238960 32433532

[9] D. Ribatti , “The Concept of Immune Surveillance Against Tumors. The First Theories,” Oncotarget 8, no. 4 (2017): 7175‐7180. 27764780 10.18632/oncotarget.12739 PMC5351698

[10] L. G. Meza Guzman , N. Keating , S. E. Nicholson , “Natural Killer Cells: Tumor Surveillance and Signaling,” Cancers 12, no. 4 (2020): 952. 32290478 10.3390/cancers12040952 PMC7226588

[11] M. E. Cruz‐Muñoz , L. Valenzuela‐Vázquez , J. Sánchez‐Herrera , J. Santa‐Olalla Tapia , “From the “Missing Self” Hypothesis to Adaptive NK Cells: Insights of NK Cell‐mediated Effector Functions in Immune Surveillance,” Journal of Leukocyte Biology 105, no. 5 (2019): 955‐971. 30848847 10.1002/JLB.MR0618-224RR

[12] E. O. Long , H. S. Kim , D. Liu , M. E. Peterson , S. Rajagopalan , “Controlling Natural Killer Cell Responses: Integration of Signals for Activation and Inhibition,” Annual Review of Immunology 31 (2013): 227‐58. 10.1146/annurev-immunol-020711-075005 PMC3868343 23516982

[13] J. R. Hwang , Y. Byeon , D. Kim , S. G. Park , “Recent Insights of T Cell Receptor‐mediated Signaling Pathways for T Cell Activation and Development,” Experimental & Molecular Medicine 52, no. 5 (2020): 750‐761. 32439954 10.1038/s12276-020-0435-8 PMC7272404

[14] K. Shah , A. Al‐Haidari , J. Sun , J. U. Kazi , “T Cell Receptor (TCR) Signaling in Health and Disease,” Signal Transduction and Targeted Therapy 6, no. 1 (2021): 412. 34897277 10.1038/s41392-021-00823-w PMC8666445

[15] J. M. Grolman , P. Weinand , D. J. Mooney , “Extracellular Matrix Plasticity as a Driver of Cell Spreading,” Proceedings of the National Academy of Sciences of the United States of America 117, no. 42 (2020): 25999‐26007. 33020289 10.1073/pnas.2008801117 PMC7584996

[16] R. S. Ganti , W. L. Lo , D. B. McAffee , J. T. Groves , A. Weiss , A. K. Chakraborty , “How the T Cell Signaling Network Processes Information to Discriminate Between Self and Agonist Ligands,” Proceedings of the National Academy of Sciences of the United States of America 117, no. 42 (2020): 26020‐26030. 33020303 10.1073/pnas.2008303117 PMC7585026

[17] D. B. McAffee , M. K. O'Dair , J. J. Lin , et al., “Discrete LAT Condensates Encode Antigen Information From Single pMHC:TCR Binding Events,” Nature Communications 13, no. 1 (2022): 7446. 10.1038/s41467-022-35093-9 PMC9718779 36460640

[18] J. Pettmann , A. Huhn , E. Abu Shah , et al., “The Discriminatory Power of the T Cell Receptor,” Elife 10 (2021): e67092. 34030769 10.7554/eLife.67092 PMC8219380

[19] M. S. Krangel , “Mechanics of T Cell Receptor Gene Rearrangement,” Current Opinion in Immunology 21, no. 2 (2009): 133‐9. 19362456 10.1016/j.coi.2009.03.009 PMC2676214

[20] Y. Shi , A. Strasser , D. R. Green , E. Latz , A. Mantovani , G. Melino , “Legacy of the Discovery of the T‐cell Receptor: 40 Years of Shaping Basic Immunology and Translational Work to Develop Novel Therapies,” Cellular & Molecular Immunology 21, no. 7 (2024): 790‐797. 38822079 10.1038/s41423-024-01168-4 PMC11214623

[21] N. Porciello , O. Franzese , L. D'Ambrosio , B. Palermo , P. Nisticò , “T‐cell Repertoire Diversity: Friend or Foe for Protective Antitumor Response?,” Journal of Experimental & Clinical Cancer Research : CR 41, no. 1 (2022): 356. 36550555 10.1186/s13046-022-02566-0 PMC9773533

[22] T. Mora , A. M. Walczak , “How Many Different Clonotypes Do Immune Repertoires Contain?,” Current Opinion in Systems Biology 18 (2019): 104‐110.

[23] Q. Qi , Y. Liu , Y. Cheng , et al., “Diversity and Clonal Selection in the human T‐cell Repertoire,” Proceedings of the National Academy of Sciences of the United States of America 111, no. 36 (2014): 13139‐44. 25157137 10.1073/pnas.1409155111 PMC4246948

[24] D. R. Leach , M. F. Krummel , J. P. Allison , “Enhancement of Antitumor Immunity by CTLA‐4 Blockade,” Science (New York, NY) 271, no. 5256 (1996): 1734‐6. 10.1126/science.271.5256.1734 8596936

[25] D. Mittal , M. M. Gubin , R. D. Schreiber , M. J. Smyth , “New Insights Into Cancer Immunoediting and Its Three Component Phases–elimination, Equilibrium and Escape,” Current Opinion in Immunology 27 (2014): 16‐25. 24531241 10.1016/j.coi.2014.01.004 PMC4388310

[26] S. A. Quezada , T. R. Simpson , K. S. Peggs , et al., “Tumor‐reactive CD4(+) T Cells Develop Cytotoxic Activity and Eradicate Large Established Melanoma After Transfer Into Lymphopenic Hosts,” The Journal of Experimental Medicine 207, no. 3 (2010): 637‐50. 20156971 10.1084/jem.20091918 PMC2839156

[27] Y. Xie , A. Akpinarli , C. Maris , et al., “Naive Tumor‐specific CD4(+) T Cells Differentiated in Vivo Eradicate Established Melanoma,” The Journal of Experimental Medicine 207, no. 3 (2010): 651‐67. 20156973 10.1084/jem.20091921 PMC2839147

[28] A. Takeuchi , S. Badr Mel , K. Miyauchi , et al., “CRTAM Determines the CD4+ Cytotoxic T Lymphocyte Lineage,” The Journal of Experimental Medicine 213, no. 1 (2016): 123‐38. 26694968 10.1084/jem.20150519 PMC4710199

[29] J. Matsuzaki , T. Tsuji , I. F. Luescher , et al., “Direct Tumor Recognition by a human CD4(+) T‐cell Subset Potently Mediates Tumor Growth Inhibition and Orchestrates Anti‐tumor Immune Responses,” Scientific Reports 5 (2015): 14896. 26447332 10.1038/srep14896 PMC4597193

[30] M. H. Spitzer , Y. Carmi , N. E. Reticker‐Flynn , “Systemic Immunity Is Required for Effective Cancer Immunotherapy,” Cell 168, no. 3 (2017): 487‐502. e15. 28111070 10.1016/j.cell.2016.12.022 PMC5312823

[31] M. Liu , F. Kuo , K. J. Capistrano , et al., “TGF‐β Suppresses Type 2 Immunity to Cancer,” Nature 587, no. 7832 (2020): 115‐120. 33087928 10.1038/s41586-020-2836-1 PMC8347705

[32] S. Li , M. Liu , M. H. Do , et al., “Cancer Immunotherapy via Targeted TGF‐β Signalling Blockade in T(H) Cells,” Nature 587, no. 7832 (2020): 121‐125. 33087933 10.1038/s41586-020-2850-3 PMC8353603

[33] L. Zhang , X. Yu , L. Zheng , et al., “Lineage Tracking Reveals Dynamic Relationships of T Cells in Colorectal Cancer,” Nature 564, no. 7735 (2018): 268‐272. 30479382 10.1038/s41586-018-0694-x

[34] S. Valpione , P. A. Mundra , E. Galvani , et al., “The T Cell Receptor Repertoire of Tumor Infiltrating T Cells Is Predictive and Prognostic for Cancer Survival,” Nature Communications 12, no. 1 (2021): 4098. 10.1038/s41467-021-24343-x PMC8253860 34215730

[35] H. S. Lawrence , Cellular and Humoral Aspects of the Hypersensitive States: A Symposium Held at the New York Academy of Medicine (P.B. Hoeber, 1959): 696.

[36] M. J. W. Sim , P. D. Sun , “T Cell Recognition of Tumor Neoantigens and Insights into T Cell Immunotherapy,” Frontiers in Immunology 13 (2022): 833017. 35222422 10.3389/fimmu.2022.833017 PMC8867076

[37] J. Schmidt , J. Chiffelle , M. A. S. Perez , et al., “Neoantigen‐specific CD8 T Cells With High Structural Avidity Preferentially Reside in and Eliminate Tumors,” Nature Communications 14, no. 1 (2023): 3188. 10.1038/s41467-023-38946-z PMC10244384 37280206

[38] P. J. Bjorkman , M. A. Saper , B. Samraoui , W. S. Bennett , J. L. Strominger , D. C. Wiley , “Structure of the human Class I Histocompatibility Antigen, HLA‐A2,” Nature 329, no. 6139 (1987): 506‐512. 3309677 10.1038/329506a0

[39] P. J. Bjorkman , M. A. Saper , B. Samraoui , W. S. Bennett , J. L. Strominger , D. C. Wiley , “The Foreign Antigen Binding Site and T Cell Recognition Regions of Class I Histocompatibility Antigens,” Nature 329, no. 6139 (1987): 512‐8. 2443855 10.1038/329512a0

[40] K. C. Garcia , M. Degano , R. L. Stanfield , et al., “An Alphabeta T Cell Receptor Structure at 2.5 A and Its Orientation in the TCR‐MHC Complex,” Science (New York, NY) 274, no. 5285 (1996): 209‐219. 10.1126/science.274.5285.209 8824178

[41] L. Klein , B. Kyewski , P. M. Allen , K. A. Hogquist , “Positive and Negative Selection of the T Cell Repertoire: What Thymocytes See (and don't see),” Nature Reviews Immunology 14, no. 6 (2014): 377‐391. 10.1038/nri3667 PMC4757912 24830344

[42] A. H. Capietto , R. Hoshyar , L. Delamarre , “Sources of Cancer Neoantigens Beyond Single‐Nucleotide Variants,” International Journal of Molecular Sciences 23, no. 17 (2022): 10131. 36077528 10.3390/ijms231710131 PMC9455963

[43] A. Peri , N. Salomon , Y. Wolf , S. Kreiter , M. Diken , Y. Samuels , “The Landscape of T Cell Antigens for Cancer Immunotherapy,” Nature Cancer 4, no. 7 (2023): 937‐954. 37415076 10.1038/s43018-023-00588-x

[44] A. Kacen , A. Javitt , M. P. Kramer , et al., “Post‐translational Modifications Reshape the Antigenic Landscape of the MHC I Immunopeptidome in Tumors,” Nature Biotechnology 41, no. 2 (2023): 239‐251. 10.1038/s41587-022-01464-2 PMC11197725 36203013

[45] W. Yang , K. W. Lee , R. M. Srivastava , et al., “Immunogenic Neoantigens Derived From Gene Fusions Stimulate T Cell Responses,” Nature Medicine 25, no. 5 (2019): 767‐775. 10.1038/s41591-019-0434-2 PMC6558662 31011208

[46] H. Kumar , R. Luo , J. Wen , C. Yang , X. Zhou , P. Kim , “FusionNeoAntigen: A Resource of Fusion Gene‐specific Neoantigens,” Nucleic Acids Res. 52, no. D1 (2024): D1276‐D1288. 37870454 10.1093/nar/gkad922 PMC10767944

[47] G. Li , S. Mahajan , S. Ma , et al., “Splicing Neoantigen Discovery With SNAF Reveals Shared Targets for Cancer Immunotherapy,” Science Translational Medicine 16, no. 730 (2024): eade2886. 38232136 10.1126/scitranslmed.ade2886 PMC11517820

[48] D. W. Kwok , N. O. Stevers , T. Nejo , et al., “Tumor‐wide RNA Splicing Aberrations Generate Immunogenic Public Neoantigens,” BioRxiv (2023). 10.1038/s41586-024-08552-0 PMC11903331 39972144

[49] M. Ji , Q. Yu , X. Z. Yang , et al., “Long‐range Alternative Splicing Contributes to Neoantigen Specificity in Glioblastoma,” Brief Bioinform 25, no. 6 (2024): bbae503. 39401143 10.1093/bib/bbae503 PMC11472750

[50] M. Zhang , J. Fritsche , J. Roszik , et al., “RNA Editing Derived Epitopes Function as Cancer Antigens to Elicit Immune Responses,” Nature Communications 9, no. 1 (2018): 3919. 10.1038/s41467-018-06405-9 PMC6156571 30254248

[51] P. Bonaventura , V. Alcazer , V. Mutez , et al., “Identification of Shared Tumor Epitopes From Endogenous Retroviruses Inducing High‐avidity Cytotoxic T Cells for Cancer Immunotherapy,” Science Advances 8, no. 4 (2022): eabj3671. 35080970 10.1126/sciadv.abj3671 PMC8791462

[52] A. Ivancevic , D. M. Simpson , O. M. Joyner , et al., “Endogenous Retroviruses Mediate Transcriptional Rewiring in Response to Oncogenic Signaling in Colorectal Cancer,” Science Advances 10, no. 29 (2024): eado1218. 39018396 10.1126/sciadv.ado1218 PMC466953

[53] R. Levy , T. Alter Regev , W. Paes , et al., “Large‐Scale Immunopeptidome Analysis Reveals Recurrent Posttranslational Splicing of Cancer‐ and Immune‐Associated Genes,” Molecular & Cellular Proteomics: MCP 22, no. 4 (2023): 100519. 36828127 10.1016/j.mcpro.2023.100519 PMC10119686

[54] C. Chong , G. Coukos , M. Bassani‐Sternberg , “Identification of Tumor Antigens With Immunopeptidomics,” Nature Biotechnology 40, no. 2 (2022): 175‐188. 10.1038/s41587-021-01038-8 34635837

[55] E. Kina , J. P. Laverdure , C. Durette , et al., “Breast Cancer Immunopeptidomes Contain Numerous Shared Tumor Antigens,” The Journal of Clinical Investigation 134, no. 1 (2024): e166740. 37906288 10.1172/JCI166740 PMC10760959

[56] S. Kalaora , A. Nagler , D. Nejman , et al., “Identification of Bacteria‐derived HLA‐bound Peptides in Melanoma,” Nature 592, no. 7852 (2021): 138‐143. 33731925 10.1038/s41586-021-03368-8 PMC9717498

[57] S. Stevanović , A. Pasetto , S. R. Helman , et al., “Landscape of Immunogenic Tumor Antigens in Successful Immunotherapy of Virally Induced Epithelial Cancer,” Science (New York, NY) 356, no. 6334 (2017): 200‐205. 10.1126/science.aak9510 PMC6295311 28408606

[58] S. L. Doran , S. Stevanović , S. Adhikary , et al., “T‐Cell Receptor Gene Therapy for Human Papillomavirus‐Associated Epithelial Cancers: A First‐in‐Human, Phase I/II Study,” Journal of Clinical Oncology : Official Journal of the American Society of Clinical Oncology 37, no. 30 (2019): 2759‐2768. 31408414 10.1200/JCO.18.02424 PMC6800280

[59] F. Huguet , S. Chevret , T. Leguay , et al., “Intensified Therapy of Acute Lymphoblastic Leukemia in Adults: Report of the Randomized GRAALL‐2005 Clinical Trial,” Journal of Clinical Oncology : Official Journal of the American Society of Clinical Oncology 36, no. 24 (2018): 2514‐2523. 29863974 10.1200/JCO.2017.76.8192

[60] A. Pal , R. Kundu , “Human Papillomavirus E6 and E7: The Cervical Cancer Hallmarks and Targets for Therapy,” Frontiers in Microbiology 10 (2019): 3116. 32038557 10.3389/fmicb.2019.03116 PMC6985034

[61] Y. Wolf , Y. Samuels , “Intratumor Heterogeneity and Antitumor Immunity Shape One another Bidirectionally,” Clinical Cancer Research: An Official Journal of the American Association for Cancer Research 28, no. 14 (2022): 2994‐3001. 35380639 10.1158/1078-0432.CCR-21-1355 PMC9306293

[62] C. Aggarwal , R. Ben‐Shachar , Y. Gao , et al., “Assessment of Tumor Mutational Burden and Outcomes in Patients with Diverse Advanced Cancers Treated with Immunotherapy,” JAMA Network Open 6, no. 5 (2023): e2311181. 37129893 10.1001/jamanetworkopen.2023.11181 PMC10155064

[63] A. M. Goodman , S. Kato , L. Bazhenova , et al., “Tumor Mutational Burden as an Independent Predictor of Response to Immunotherapy in Diverse Cancers,” Molecular Cancer Therapeutics 16, no. 11 (2017): 2598‐2608. 28835386 10.1158/1535-7163.MCT-17-0386 PMC5670009

[64] R. M. Samstein , C. H. Lee , A. N. Shoushtari , et al., “Tumor Mutational Load Predicts Survival After Immunotherapy Across Multiple Cancer Types,” Nature Genetics 51, no. 2 (2019): 202‐206. 30643254 10.1038/s41588-018-0312-8 PMC6365097

[65] A. Marabelle , M. Fakih , J. Lopez , et al., “Association of Tumour Mutational Burden With Outcomes in Patients With Advanced Solid Tumours Treated With pembrolizumab: Prospective Biomarker Analysis of the Multicohort, Open‐label, Phase 2 KEYNOTE‐158 Study,” The Lancet Oncology 21, no. 10 (2020): 1353‐1365. 32919526 10.1016/S1470-2045(20)30445-9

[66] A. M. Kirk , J. C. Crawford , C. H. Chou , et al., “DNAJB1‐PRKACA Fusion Neoantigens Elicit Rare Endogenous T Cell Responses That Potentiate Cell Therapy for Fibrolamellar Carcinoma,” Cell Reports Medicine 5, no. 3 (2024): 101469. 38508137 10.1016/j.xcrm.2024.101469 PMC10983114

[67] Y. Wolf , Y. Sameuls , “Neoantigens in Cancer Immunotherapy: Quantity vs. quality,” Molecular Oncology 17, no. 8 (2023): 1457‐1459. 37370255 10.1002/1878-0261.13483 PMC10399717

[68] S. S. Chandran , J. Ma , M. G. Klatt , et al., “Immunogenicity and Therapeutic Targeting of a Public Neoantigen Derived From Mutated PIK3CA,” Nature Medicine 28, no. 5 (2022): 946‐957. 10.1038/s41591-022-01786-3 PMC9117146 35484264

[69] C. Puig‐Saus , B. Sennino , S. Peng , et al., “Neoantigen‐targeted CD8(+) T Cell Responses With PD‐1 Blockade Therapy,” Nature 615, no. 7953 (2023): 697‐704. 36890230 10.1038/s41586-023-05787-1 PMC10441586

[70] J. J. Y. Lin , S. T. Low‐Nam , K. N. Alfieri , D. B. McAffee , N. C. Fay , J. T. Groves , “Mapping the Stochastic Sequence of Individual Ligand‐receptor Binding Events to Cellular Activation: T Cells Act on the Rare Events,” Science Signaling 12, no. 564 (2019): eaat8715. 30647147 10.1126/scisignal.aat8715 PMC6598675

[71] M. Shakiba , P. Zumbo , G. Espinosa‐Carrasco , et al., “TCR Signal Strength Defines Distinct Mechanisms of T Cell Dysfunction and Cancer Evasion,” The Journal of Experimental Medicine 219, no. 2 (2022): e20201966. 34935874 10.1084/jem.20201966 PMC8704919

[72] C. Ragone , B. Cavalluzzo , A. Mauriello , M. Tagliamonte , L. Buonaguro , “Lack of Shared Neoantigens in Prevalent Mutations in Cancer,” Journal of Translational Medicine 22, no. 1 (2024): 344. 38600547 10.1186/s12967-024-05110-0 PMC11005154

[73] I. Milo , M. Bedora‐Faure , Z. Garcia , et al., “The Immune System Profoundly Restricts Intratumor Genetic Heterogeneity,” Science Immunology 3, no. 29 (2018): eaat1435. 30470696 10.1126/sciimmunol.aat1435

[74] Y. H. Chien , C. Meyer , M. Bonneville , “γδ T Cells: First Line of Defense and Beyond,” Annual Review of Immunology 32 (2014): 121‐55. 10.1146/annurev-immunol-032713-120216 24387714

[75] J. C. Ribot , N. Lopes , B. Silva‐Santos , “γδ T Cells in Tissue Physiology and Surveillance,” Nature Reviews Immunology 21, no. 4 (2021): 221‐232. 10.1038/s41577-020-00452-4 33057185

[76] Y. Hu , Q. Hu , Y. Li , et al., “γδ T Cells: Origin and Fate, Subsets, Diseases and Immunotherapy,” Signal Transduction and Targeted Therapy 8, no. 1 (2023): 434. 37989744 10.1038/s41392-023-01653-8 PMC10663641

[77] J. Guo , R. R. Chowdhury , V. Mallajosyula , et al., “γδ T Cell Antigen Receptor Polyspecificity Enables T Cell Responses to a Broad Range of Immune Challenges,” Proceedings of the National Academy of Sciences of the United States of America 121, no. 4 (2024): e2315592121. 38227652 10.1073/pnas.2315592121 PMC10823224

[78] M. Deseke , I. Prinz , “Ligand Recognition by the Γδ TCR and Discrimination Between Homeostasis and Stress Conditions,” Cellular & Molecular Immunology 17, no. 9 (2020): 914‐924. 32709926 10.1038/s41423-020-0503-y PMC7608190

[79] S. Kalyan , D. Kabelitz , “Defining the Nature of human Γδ T Cells: A Biographical Sketch of the Highly Empathetic,” Cellular and Molecular Immunology 10, no. 1 (2013): 21. 23085947 10.1038/cmi.2012.44 PMC4003173

[80] M. Eberl , M. Hintz , A. Reichenberg , A. K. Kollas , J. Wiesner , H. Jomaa , “Microbial Isoprenoid Biosynthesis and human Gammadelta T Cell Activation,” FEBS Letters 544, no. 1‐3 (2003): 4‐10. 12782281 10.1016/s0014-5793(03)00483-6

[81] H. J. Gober , M. Kistowska , L. Angman , P. Jenö , L. Mori , G. De Libero , “Human T Cell Receptor Gammadelta Cells Recognize Endogenous Mevalonate Metabolites in Tumor Cells,” The Journal of Experimental Medicine 197, no. 2 (2003): 163‐8. 12538656 10.1084/jem.20021500 PMC2193814

[82] L. Yuan , X. Ma , Y. Yang , et al., “Phosphoantigens Glue Butyrophilin 3A1 and 2A1 to Activate Vγ9Vδ2 T Cells,” Nature 621, no. 7980 (2023): 840‐848. 37674084 10.1038/s41586-023-06525-3 PMC10533412

[83] F. Galvez‐Cancino , M. Navarrete , G. Beattie , et al., “Regulatory T Cell Depletion Promotes Myeloid Cell Activation and Glioblastoma Response to Anti‐PD1 and Tumor‐targeting Antibodies,” Immunity 58, no. 5 (2025): 1236‐1253. 40280128 10.1016/j.immuni.2025.03.021

[84] S. Dadi , S. Chhangawala , B. M. Whitlock , et al., “Cancer Immunosurveillance by Tissue‐Resident Innate Lymphoid Cells and Innate‐Like T Cells,” Cell 164, no. 3 (2016): 365‐77. 26806130 10.1016/j.cell.2016.01.002 PMC4733424

[85] C. Chou , X. Zhang , C. Krishna , et al., “Programme of Self‐reactive Innate‐Like T Cell‐mediated Cancer Immunity,” Nature 605, no. 7908 (2022): 139‐145. 35444279 10.1038/s41586-022-04632-1 PMC9250102

[86] E. R. Kansler , S. Dadi , C. Krishna , et al., “Cytotoxic Innate Lymphoid Cells Sense Cancer Cell‐expressed Interleukin‐15 to Suppress human and Murine Malignancies,” Nature Immunology 23, no. 6 (2022): 904‐915. 35618834 10.1038/s41590-022-01213-2 PMC9202504

[87] J. Zhang , A. M. Li , E. R. Kansler , Li MO , “Cancer Immunity by Tissue‐resident Type 1 Innate Lymphoid Cells and Killer Innate‐Like T Cells,” Immunological Reviews 323, no. 1 (2024): 150‐163. 38506480 10.1111/imr.13319 PMC11102320

[88] A. Baessler , D. A. A. Vignali , “T Cell Exhaustion,” Annual Review of Immunology 42, no. 1 (2024): 179‐206. 10.1146/annurev-immunol-090222-110914 38166256

[89] X. He , C. Xu , “Immune Checkpoint Signaling and Cancer Immunotherapy,” Cell Research 30, no. 8 (2020): 660‐669. 32467592 10.1038/s41422-020-0343-4 PMC7395714

[90] E. J. Wherry , S. J. Ha , S. M. Kaech , et al., “Molecular Signature of CD8+ T Cell Exhaustion During Chronic Viral Infection,” Immunity 27, no. 4 (2007): 670‐84. 17950003 10.1016/j.immuni.2007.09.006

[91] D. T. Utzschneider , S. S. Gabriel , D. Chisanga , et al., “Early Precursor T Cells Establish and Propagate T Cell Exhaustion in Chronic Infection,” Nature Immunology 21, no. 10 (2020): 1256‐1266. 32839610 10.1038/s41590-020-0760-z

[92] P. Hammarström , X. Jiang , A. R. Hurshman , E. T. Powers , J. W. Kelly , “Sequence‐dependent Denaturation Energetics: A Major Determinant in Amyloid Disease Diversity,” Proceedings of the National Academy of Sciences of the United States of America 99, no. Suppl 4 (2002): 16427‐32. 12351683 10.1073/pnas.202495199 PMC139904

[93] A. Chow , K. Perica , C. A. Klebanoff , J. D. Wolchok , “Clinical Implications of T Cell Exhaustion for Cancer Immunotherapy,” Nature Reviews Clinical Oncology 19, no. 12 (2022): 775‐790. 10.1038/s41571-022-00689-z PMC10984554 36216928

[94] M. Sade‐Feldman , K. Yizhak , S. L. Bjorgaard , et al., “Defining T Cell States Associated With Response to Checkpoint Immunotherapy in Melanoma,” Cell 175, no. 4 (2018): 998‐1013. e20. 30388456 10.1016/j.cell.2018.10.038 PMC6641984

[95] J. Nah , R. H. Seong , “Krüppel‐Like Factor 4 Regulates the Cytolytic Effector Function of Exhausted CD8 T Cells,” Science Advances 8, no. 47 (2022): eadc9346. 36427304 10.1126/sciadv.adc9346 PMC9699681

[96] C. S. Eberhardt , H. T. Kissick , M. R. Patel , et al., “Functional HPV‐specific PD‐1(+) Stem‐Like CD8 T Cells in Head and Neck Cancer,” Nature 597, no. 7875 (2021): 279‐284. 34471285 10.1038/s41586-021-03862-z PMC10201342

[97] D. S. Thommen , “The First Shall (Be) Last: Understanding Durable T Cell Responses in Immunotherapy,” Immunity 50, no. 1 (2019): 6‐8. 30650381 10.1016/j.immuni.2018.12.029

[98] A. Kallies , D. Zehn , D. T. Utzschneider , “Precursor Exhausted T Cells: Key to Successful Immunotherapy?,” Nature Reviews Immunology 20, no. 2 (2020): 128‐136. 10.1038/s41577-019-0223-7 31591533

[99] B. C. Miller , D. R. Sen , R. Al Abosy , “Subsets of Exhausted CD8(+) T Cells Differentially Mediate Tumor Control and Respond to Checkpoint Blockade,” Nature Immunology 20, no. 3 (2019): 326‐336. 30778252 10.1038/s41590-019-0312-6 PMC6673650

[100] R. Zander , W. Cui , “Exhausted CD8(+) T Cells Face a Developmental Fork in the Road,” Trends in Immunology 44, no. 4 (2023): 276‐286. 36907685 10.1016/j.it.2023.02.006 PMC10569258

[101] T. Sekine , A. Perez‐Potti , S. Nguyen , et al., “TOX Is Expressed by Exhausted and Polyfunctional human Effector Memory CD8(+) T Cells,” Science Immunology 5, no. 49 (2020): eaba7918. 32620560 10.1126/sciimmunol.aba7918

[102] P. Chatterjee , N. Patsoukis , G. J. Freeman , V. A. Boussiotis , “Distinct Roles of PD‐1 Itsm and ITIM in Regulating Interactions with SHP‐2, ZAP‐70 and Lck, and PD‐1‐Mediated Inhibitory Function,” Blood 122, no. 21 (2013): 191‐191.

[103] E. Hui , J. Cheung , J. Zhu , et al., “T Cell Costimulatory Receptor CD28 Is a Primary Target for PD‐1‐mediated Inhibition,” Science (New York, NY) 355, no. 6332 (2017): 1428‐1433. 10.1126/science.aaf1292 PMC6286077 28280247

[104] G. Rota , C. Niogret , A. T. Dang , et al., “Shp‐2 Is Dispensable for Establishing T Cell Exhaustion and for PD‐1 Signaling in Vivo,” Cell Reports 23, no. 1 (2018): 39‐49. 29617671 10.1016/j.celrep.2018.03.026

[105] J. Celis‐Gutierrez , P. Blattmann , Y. Zhai , et al., “Quantitative Interactomics in Primary T Cells Provides a Rationale for Concomitant PD‐1 and BTLA Coinhibitor Blockade in Cancer Immunotherapy,” Cell Reports 27, no. 11 (2019): 3315‐3330. e7. 31189114 10.1016/j.celrep.2019.05.041 PMC6581740

[106] T. Yokosuka , M. Takamatsu , W. Kobayashi‐Imanishi , A. Hashimoto‐Tane , M. Azuma , T. Saito , “Programmed Cell Death 1 Forms Negative Costimulatory Microclusters That Directly Inhibit T Cell Receptor Signaling by Recruiting Phosphatase SHP2,” The Journal of Experimental Medicine 209, no. 6 (2012): 1201‐17. 22641383 10.1084/jem.20112741 PMC3371732

[107] K. A. Sheppard , L. J. Fitz , J. M. Lee , et al., “PD‐1 Inhibits T‐cell Receptor Induced Phosphorylation of the ZAP70/CD3zeta Signalosome and Downstream Signaling to PKCtheta,” FEBS Letters 574, no. 1‐3 (2004): 37‐41. 15358536 10.1016/j.febslet.2004.07.083

[108] P. A. van der Merwe , D. L. Bodian , S. Daenke , P. Linsley , S. J. Davis , “CD80 (B7‐1) binds both CD28 and CTLA‐4 With a Low Affinity and Very Fast Kinetics,” The Journal of Experimental Medicine 185, no. 3 (1997): 393‐403. 9053440 10.1084/jem.185.3.393 PMC2196039

[109] Y. Zhu , S. Yao , L. Chen , “Cell Surface Signaling Molecules in the Control of Immune Responses: A Tide Model,” Immunity 34, no. 4 (2011): 466‐78. 21511182 10.1016/j.immuni.2011.04.008 PMC3176719

[110] T. Shiratori , S. Miyatake , H. Ohno , et al., “Tyrosine Phosphorylation Controls Internalization of CTLA‐4 by Regulating Its Interaction With Clathrin‐associated Adaptor Complex AP‐2,” Immunity 6, no. 5 (1997): 583‐9. 9175836 10.1016/s1074-7613(00)80346-5

[111] L. E. Marengère , P. Waterhouse , G. S. Duncan , H. W. Mittrücker , G. S. Feng , T. W. Mak , “Regulation of T Cell Receptor Signaling by Tyrosine Phosphatase SYP Association With CTLA‐4,” Science (New York, NY) 272, no. 5265 (1996): 1170‐3. 10.1126/science.272.5265.1170 8638161

[112] A. L. Mellor , P. Chandler , B. Baban , et al., “Specific Subsets of Murine Dendritic Cells Acquire Potent T Cell Regulatory Functions Following CTLA4‐mediated Induction of Indoleamine 2,3 Dioxygenase,” International Immunology 16, no. 10 (2004): 1391‐401. 15351783 10.1093/intimm/dxh140

[113] K. L. Clayton , M. S. Haaland , M. B. Douglas‐Vail , “T Cell Ig and Mucin Domain‐containing Protein 3 Is Recruited to the Immune Synapse, Disrupts Stable Synapse Formation, and Associates With Receptor Phosphatases,” Journal of Immunology 192, no. 2 (2014): 782‐91. 10.4049/jimmunol.1302663 PMC4214929 24337741

[114] Y. H. Huang , C. Zhu , Y. Kondo , et al., “CEACAM1 regulates TIM‐3‐mediated Tolerance and Exhaustion,” Nature 517, no. 7534 (2015): 386‐90. 25363763 10.1038/nature13848 PMC4297519

[115] S. Chen , J. Chen , Y. Kong , et al., “Knockdown of TIM3 Hampers Dendritic Cell Maturation and Induces Immune Suppression by Modulating T‐Cell Responses,” International Journal of Molecular Sciences 26, no. 9 (2025): 4332. 40362568 10.3390/ijms26094332 PMC12072576

[116] M. Nakayama , H. Akiba , K. Takeda , et al., “Tim‐3 Mediates Phagocytosis of Apoptotic Cells and Cross‐presentation,” Blood 113, no. 16 (2009): 3821‐30. 19224762 10.1182/blood-2008-10-185884

[117] K. Wong , P. A. Valdez , C. Tan , S. Yeh , J. A. Hongo , W. Ouyang , “Phosphatidylserine Receptor Tim‐4 Is Essential for the Maintenance of the Homeostatic state of Resident Peritoneal Macrophages,” Proceedings of the National Academy of Sciences of the United States of America 107, no. 19 (2010): 8712‐7. 20421466 10.1073/pnas.0910929107 PMC2889355

[118] K. Chemin , C. Gerstner , V. Malmström , “Effector Functions of CD4+ T Cells at the Site of Local Autoimmune Inflammation‐Lessons from Rheumatoid Arthritis,” Frontiers in Immunology 10 (2019): 353. 30915067 10.3389/fimmu.2019.00353 PMC6422991

[119] J. Wang , M. F. Sanmamed , I. Datar , et al., “Fibrinogen‐Like Protein 1 Is a Major Immune Inhibitory Ligand of LAG‐3,” Cell 176, no. 1‐2 (2019): 334‐347. e12. 30580966 10.1016/j.cell.2018.11.010 PMC6365968

[120] Y. Jiang , A. Dai , Y. Huang , et al., “Ligand‐induced Ubiquitination Unleashes LAG3 Immune Checkpoint Function by Hindering Membrane Sequestration of Signaling Motifs,” Cell 188, no. 9 (2025): 2354‐2371. e18. 40101708 10.1016/j.cell.2025.02.014

[121] J. M. Chemnitz , A. R. Lanfranco , I. Braunstein , J. L. Riley , “B and T Lymphocyte Attenuator‐mediated Signal Transduction Provides a Potent Inhibitory Signal to Primary human CD4 T Cells That Can be Initiated by Multiple Phosphotyrosine Motifs,” Journal of Immunology 176, no. 11 (2006): 6603‐14. 10.4049/jimmunol.176.11.6603 16709818

[122] X. Xu , B. Hou , A. Fulzele , et al., “PD‐1 and BTLA Regulate T Cell Signaling Differentially and Only Partially Through SHP1 and SHP2,” Journal of Cell Biology 219, no. 6 (2020): e201905085. 32437509 10.1083/jcb.201905085 PMC7265324

[123] X. Xu , T. Masubuchi , Q. Cai , Y. Zhao , E. Hui , “Molecular Features Underlying Differential SHP1/SHP2 Binding of Immune Checkpoint Receptors,” Elife 10 (2021): e74276. 34734802 10.7554/eLife.74276 PMC8631942

[124] S. Mélique , A. Vadel , N. Rouquié , et al., “THEMIS Promotes T Cell Development and Maintenance by Rising the Signaling Threshold of the Inhibitory Receptor BTLA,” Proceedings of the National Academy of Sciences of the United States of America 121, no. 20 (2024): e2318773121. 38713628 10.1073/pnas.2318773121 PMC11098085

[125] A. Kharel , J. Shen , R. Brown , et al., “Loss of PBAF Promotes Expansion and Effector Differentiation of CD8(+) T Cells During Chronic Viral Infection and Cancer,” Cell Reports 42, no. 6 (2023): 112649. 37330910 10.1016/j.celrep.2023.112649 PMC10592487

[126] J. H. Cha , L. C. Chan , C. W. Li , J. L. Hsu , M. C. Hung , “Mechanisms Controlling PD‐L1 Expression in Cancer,” Molecular Cell 76, no. 3 (2019): 359‐370. 31668929 10.1016/j.molcel.2019.09.030 PMC6981282

[127] K. Kersten , K. H. Hu , A. J. Combes , et al., “Spatiotemporal co‐dependency Between Macrophages and Exhausted CD8(+) T Cells in Cancer,” Cancer Cell 40, no. 6 (2022): 624‐638. e9. 35623342 10.1016/j.ccell.2022.05.004 PMC9197962

[128] B. G. Nixon , F. Kuo , L. Ji , et al., “Tumor‐associated Macrophages Expressing the Transcription Factor IRF8 Promote T Cell Exhaustion in Cancer,” Immunity 55, no. 11 (2022): 2044‐2058. 36288724 10.1016/j.immuni.2022.10.002 PMC9649891

[129] N. Prokhnevska , M. A. Cardenas , R. M. Valanparambil , et al., “CD8(+) T Cell Activation in Cancer Comprises an Initial Activation Phase in Lymph Nodes Followed by Effector Differentiation Within the Tumor,” Immunity 56, no. 1 (2023): 107‐124. e5. 36580918 10.1016/j.immuni.2022.12.002 PMC10266440

[130] D. Zehn , R. Thimme , E. Lugli , G. P. de Almeida , A. Oxenius , “‘Stem‐Like’ precursors Are the Fount to Sustain Persistent CD8(+) T Cell Responses,” Nature Immunology 23, no. 6 (2022): 836‐847. 35624209 10.1038/s41590-022-01219-w

[131] X. Lan , T. Mi , S. Alli , et al., “Antitumor Progenitor Exhausted CD8(+) T Cells Are Sustained by TCR Engagement,” Nature Immunology 25, no. 6 (2024): 1046‐1058. 38816618 10.1038/s41590-024-01843-8

[132] K. Mittal , J. Ebos , B. Rini , “Angiogenesis and the Tumor Microenvironment: Vascular Endothelial Growth Factor and Beyond,” Seminars in Oncology 41, no. 2 (2014): 235‐51. 24787295 10.1053/j.seminoncol.2014.02.007

[133] U. Das , D. U. Kapoor , S. Singh , B. G. Prajapati , “Unveiling the Potential of chitosan‐coated Lipid Nanoparticles in Drug Delivery for Management of Critical Illness: A Review,” Z Naturforsch C J Biosci 79, no. 5‐6 (2024): 107‐124. 38721838 10.1515/znc-2023-0181

[134] J. Waibl Polania , A. Hoyt‐Miggelbrink , W. H. Tomaszewski , et al., “Antigen Presentation by Tumor‐associated Macrophages Drives T Cells From a Progenitor Exhaustion state to Terminal Exhaustion,” Immunity 58, no. 1 (2025): 232‐246. e6. 39724910 10.1016/j.immuni.2024.11.026

[135] J. L. Raynor , H. Chi , “Nutrients: Signal 4 in T Cell Immunity,” The Journal of Experimental Medicine 221, no. 3 (2024): e20221839. 38411744 10.1084/jem.20221839 PMC10899091

[136] S. K. Seo , B. Kwon , “Immune Regulation Through Tryptophan Metabolism,” Experimental & Molecular Medicine 55, no. 7 (2023): 1371‐1379. 37394584 10.1038/s12276-023-01028-7 PMC10394086

[137] Y. Zheng , R. Xu , X. Chen , et al., “Metabolic Gatekeepers: Harnessing Tumor‐derived Metabolites to Optimize T Cell‐based Immunotherapy Efficacy in the Tumor Microenvironment,” Cell Death & Disease 15, no. 10 (2024): 775. 39461979 10.1038/s41419-024-07122-6 PMC11513100

[138] D. Vijayan , A. Young , M. W. L. Teng , M. J. Smyth , “Targeting Immunosuppressive Adenosine in Cancer,” Nature Reviews Cancer 17, no. 12 (2017): 709‐724. 29059149 10.1038/nrc.2017.86

[139] D. Sharma , J. D. Farrar , “Adrenergic Regulation of Immune Cell Function and Inflammation,” Seminars in immunopathology 42, no. 6 (2020): 709‐717. 33219396 10.1007/s00281-020-00829-6 PMC7678770

[140] A. Shadboorestan , M. Koual , J. Dairou , X. Coumoul , “The Role of the Kynurenine/AhR Pathway in Diseases Related to Metabolism and Cancer,” Int J Tryptophan Res 16 (2023): 11786469231185102. 37719171 10.1177/11786469231185102 PMC10503295

[141] J. W. Dean , L. Zhou , “Cell‐intrinsic View of the Aryl Hydrocarbon Receptor in Tumor Immunity,” Trends in Immunology 43, no. 3 (2022): 245‐258. 35131180 10.1016/j.it.2022.01.008 PMC8882133

[142] S. Punyawatthananukool , R. Matsuura , T. Wongchang , et al., “Prostaglandin E(2)‐EP2/EP4 Signaling Induces Immunosuppression in human Cancer by Impairing Bioenergetics and Ribosome Biogenesis in Immune Cells,” Nature Communications 15, no. 1 (2024): 9464. 10.1038/s41467-024-53706-3 PMC11530437 39487111

[143] J. P. Böttcher , E. Bonavita , P. Chakravarty , et al., “NK Cells Stimulate Recruitment of cDC1 Into the Tumor Microenvironment Promoting Cancer Immune Control,” Cell 172, no. 5 (2018): 1022‐1037. e14. 29429633 10.1016/j.cell.2018.01.004 PMC5847168

[144] F. Bayerl , P. Meiser , S. Donakonda , et al., “Tumor‐derived Prostaglandin E2 Programs cDC1 Dysfunction to Impair Intratumoral Orchestration of Anti‐cancer T Cell Responses,” Immunity 56, no. 6 (2023): 1341‐1358. e11. 37315536 10.1016/j.immuni.2023.05.011

[145] D. Schadendorf , F. S. Hodi , C. Robert , et al., “Pooled Analysis of Long‐Term Survival Data from Phase II and Phase III Trials of Ipilimumab in Unresectable or Metastatic Melanoma,” Journal of Clinical Oncology : Official Journal of the American Society of Clinical Oncology 33, no. 17 (2015): 1889‐94. 25667295 10.1200/JCO.2014.56.2736 PMC5089162

[146] J. S. Weber , “Current Perspectives on Immunotherapy,” Seminars in Oncology 41, no. Suppl 5 (2014): S14‐S29. 10.1053/j.seminoncol.2014.09.003 25438996

[147] S. L. Topalian , A. H. Sharpe , “Balance and Imbalance in the Immune System: Life on the Edge,” Immunity 41, no. 5 (2014): 682‐4. 25517610 10.1016/j.immuni.2014.11.005 PMC4710093

[148] M. Yi , X. Zheng , M. Niu , S. Zhu , H. Ge , K. Wu , “Combination Strategies With PD‐1/PD‐L1 Blockade: Current Advances and Future Directions,” Molecular cancer 21, no. 1 (2022): 28. 35062949 10.1186/s12943-021-01489-2 PMC8780712

[149] D. S. Thommen , D. S. Peeper , “Rational Combination of Cancer Therapies With PD1 Axis Blockade,” Nature Reviews Cancer (2024). 10.1038/s41568-024-00727-1 39080491

[150] T. Okazaki , I. M. Okazaki , J. Wang , et al., “PD‐1 and LAG‐3 Inhibitory co‐receptors Act Synergistically to Prevent Autoimmunity in Mice,” The Journal of Experimental Medicine 208, no. 2 (2011): 395‐407. 21300912 10.1084/jem.20100466 PMC3039848

[151] S. R. Woo , M. E. Turnis , M. V. Goldberg , et al., “Immune Inhibitory Molecules LAG‐3 and PD‐1 Synergistically Regulate T‐cell Function to Promote Tumoral Immune Escape,” Cancer Research 72, no. 4 (2012): 917‐27. 22186141 10.1158/0008-5472.CAN-11-1620 PMC3288154

[152] T. J. Panella , S. S. Thomas , M. McKean , et al., “A Phase 3 Trial Comparing fianlimab (anti–LAG‐3) plus cemiplimab (anti–PD‐1) to pembrolizumab in Patients With Completely Resected High‐risk Melanoma,” American Society of Clinical Oncology 41, no. 16 (2023).

[153] A. Weiss , “Peeking into the Black Box of T Cell Receptor Signaling,” Annual Review of Immunology 42, no. 1 (2024): 1‐20. 10.1146/annurev-immunol-090222-112028 37788477

[154] D. T. McManus , R. M. Valanparambil , C. B. Medina , et al., “An Early Precursor CD8(+) T Cell That Adapts to Acute or Chronic Viral Infection,” Nature 640, no. 8059 (2025): 772‐781. 39778710 10.1038/s41586-024-08562-y PMC13375091

[155] S. Kurtulus , A. Madi , G. Escobar , et al., “Checkpoint Blockade Immunotherapy Induces Dynamic Changes in PD‐1(‐)CD8(+) Tumor‐Infiltrating T Cells,” Immunity 50, no. 1 (2019): 181‐194. e6. 30635236 10.1016/j.immuni.2018.11.014 PMC6336113

[156] P. L. Chen , W. Roh , A. Reuben , et al., “Analysis of Immune Signatures in Longitudinal Tumor Samples Yields Insight Into Biomarkers of Response and Mechanisms of Resistance to Immune Checkpoint Blockade,” Cancer Discovery 6, no. 8 (2016): 827‐37. 27301722 10.1158/2159-8290.CD-15-1545 PMC5082984

[157] Y. Xie , F. Liu , Y. Wu , et al., “Inflammation in Cancer: Therapeutic Opportunities From New Insights,” Molecular cancer 24, no. 1 (2025): 51. 39994787 10.1186/s12943-025-02243-8 PMC11849313

[158] N. Shifrin , D. H. Raulet , M. Ardolino , “NK Cell Self Tolerance, Responsiveness and Missing Self Recognition,” Seminars in Immunology 26, no. 2 (2014): 138‐44. 24629893 10.1016/j.smim.2014.02.007 PMC3984600

[159] D. C. P. Wong , J. L. Ding , “The Mechanobiology of NK Cells‐ ‘Forcing NK to Sense’ target Cells,” Biochimica Et Biophysica Acta Reviews on Cancer 1878, no. 2 (2023): 188860. 36791921 10.1016/j.bbcan.2023.188860

[160] T. J. Laskowski , A. Biederstädt , K. Rezvani , “Natural Killer Cells in Antitumour Adoptive Cell Immunotherapy,” Nature Reviews Cancer 22, no. 10 (2022): 557‐575. 35879429 10.1038/s41568-022-00491-0 PMC9309992

[161] L. Peng , G. Sferruzza , L. Yang , L. Zhou , S. Chen , “CAR‐T and CAR‐NK as Cellular Cancer Immunotherapy for Solid Tumors,” Cellular & Molecular Immunology 21, no. 10 (2024): 1089‐1108. 39134804 10.1038/s41423-024-01207-0 PMC11442786

[162] S. Guedan , M. Ruella , C. H. June , “Emerging Cellular Therapies for Cancer,” Annual Review of Immunology 37 (2019): 145‐171. 10.1146/annurev-immunol-042718-041407 PMC7399614 30526160

[163] W. Wang , Y. Liu , Z. He , et al., “Breakthrough of Solid Tumor Treatment: CAR‐NK Immunotherapy,” Cell Death Discovery 10, no. 1 (2024): 40. 38245520 10.1038/s41420-024-01815-9 PMC10799930

[164] A. S. Bear , J. A. Fraietta , V. K. Narayan , M. O'Hara , N. B. Haas , “Adoptive Cellular Therapy for Solid Tumors,” American Society of Clinical Oncology Educational Book American Society of Clinical Oncology Annual Meeting 41 (2021): 57‐65. 34010040 10.1200/EDBK_321115

[165] M. Morotti , A. Albukhari , A. Alsaadi , et al., “Promises and Challenges of Adoptive T‐cell Therapies for Solid Tumours,” British Journal of Cancer 124, no. 11 (2021): 1759‐1776. 33782566 10.1038/s41416-021-01353-6 PMC8144577

[166] S. Secondino , C. Canino , D. Alaimo , et al., “Clinical Trials of Cellular Therapies in Solid Tumors,” Cancers 15, no. 14 (2023): 3667. 37509328 10.3390/cancers15143667 PMC10377409

[167] C. E. Brown , D. Alizadeh , R. Starr , et al., “Regression of Glioblastoma After Chimeric Antigen Receptor T‐Cell Therapy,” New England Journal of Medicine 375, no. 26 (2016): 2561‐2569. 28029927 10.1056/NEJMoa1610497 PMC5390684

[168] C. E. Brown , D. Alizadeh , R. Starr , et al., “Regression of Glioblastoma After Chimeric Antigen Receptor T‐Cell Therapy,” The New England Journal of Medicine 375, no. 26 (2016): 2561‐9. 28029927 10.1056/NEJMoa1610497 PMC5390684

[169] C. U. Louis , B. Savoldo , G. Dotti , et al., “Antitumor Activity and Long‐term Fate of Chimeric Antigen Receptor–positive T Cells in Patients With Neuroblastoma,” Blood 118, no. 23 (2011): 6050‐6056. 21984804 10.1182/blood-2011-05-354449 PMC3234664

[170] K. M. Cappell , J. N. Kochenderfer , “Long‐term Outcomes Following CAR T Cell Therapy: What We Know so Far,” Nature Reviews Clinical Oncology 20, no. 6 (2023): 359‐371. 10.1038/s41571-023-00754-1 PMC10100620 37055515

[171] G. Schett , A. Mackensen , D. Mougiakakos , “CAR T‐cell Therapy in Autoimmune Diseases,” Lancet (London, England) 402, no. 10416 (2023): 2034‐2044. 37748491 10.1016/S0140-6736(23)01126-1

[172] J. Zhang , J. Li , Y. Hou , et al., “Osr2 functions as a Biomechanical Checkpoint to Aggravate CD8+ T Cell Exhaustion in Tumor,” Cell 187, no. 13 (2024): 3409‐3426. e24. 38744281 10.1016/j.cell.2024.04.023

[173] M. A. Postow , R. Sidlow , M. D. Hellmann , “Immune‐Related Adverse Events Associated With Immune Checkpoint Blockade,” The New England Journal of Medicine 378, no. 2 (2018): 158‐168. 29320654 10.1056/NEJMra1703481

[174] Y. Jiang , Y. Li , B. Zhu , “T‐cell Exhaustion in the Tumor Microenvironment,” Cell Death & Disease 6, no. 6 (2015): e1792. 26086965 10.1038/cddis.2015.162 PMC4669840

[175] C. C. Zebley , D. Zehn , S. Gottschalk , H. Chi , “T Cell Dysfunction and Therapeutic Intervention in Cancer,” Nature Immunology 25, no. 8 (2024): 1344‐1354. 39025962 10.1038/s41590-024-01896-9 PMC11616736

[176] K. A. Connolly , M. Kuchroo , A. Venkat , et al., “A Reservoir of Stem‐Like CD8(+) T Cells in the Tumor‐draining Lymph Node Preserves the Ongoing Antitumor Immune Response,” Science Immunology 6, no. 64 (2021): eabg7836. 34597124 10.1126/sciimmunol.abg7836 PMC8593910

[177] T. Gebhardt , S. L. Park , I. A. Parish , “Stem‐Like Exhausted and Memory CD8(+) T Cells in Cancer,” Nature Reviews Cancer 23, no. 11 (2023): 780‐798. 37821656 10.1038/s41568-023-00615-0

[178] G. Oliveira , C. J. Wu , “Dynamics and Specificities of T Cells in Cancer Immunotherapy,” Nature Reviews Cancer 23, no. 5 (2023): 295‐316. 37046001 10.1038/s41568-023-00560-y PMC10773171

[179] Z. Liu , Y. Zhang , N. Ma , et al., “Progenitor‐Like Exhausted SPRY1(+)CD8(+) T Cells Potentiate Responsiveness to Neoadjuvant PD‐1 Blockade in Esophageal Squamous Cell Carcinoma,” Cancer Cell 41, no. 11 (2023): 1852‐1870. e9. 37832554 10.1016/j.ccell.2023.09.011

[180] I. Mellman , D. S. Chen , T. Powles , S. J. Turley , “The Cancer‐immunity Cycle: Indication, Genotype, and Immunotype,” Immunity 56, no. 10 (2023): 2188‐2205. 37820582 10.1016/j.immuni.2023.09.011

[181] A. M. Starzer , M. Preusser , A. S. Berghoff , “Immune Escape Mechanisms and Therapeutic Approaches in Cancer: The Cancer‐immunity Cycle,” Therapeutic Advances in Medical Oncology 14 (2022): 17588359221096219. 35510032 10.1177/17588359221096219 PMC9058458

[182] M. Cabeza‐Cabrerizo , A. Cardoso , C. M. Minutti , M. Pereira da Costa , C. Reis e Sousa , “Dendritic Cells Revisited,” Annual Review of Immunology 39 (2021): 131‐166. 10.1146/annurev-immunol-061020-053707 33481643

[183] A. Katsnelson , “Kicking off Adaptive Immunity: The Discovery of Dendritic Cells,” The Journal of Experimental Medicine 203, no. 7 (2006): 1622. 16886239 10.1084/jem.2037fta PMC2118351

[184] T. L. Murphy , G. E. Grajales‐Reyes , X. Wu , et al., “Transcriptional Control of Dendritic Cell Development,” Annual Review of Immunology 34 (2016): 93‐119. 10.1146/annurev-immunol-032713-120204 PMC5135011 26735697

[185] T. L. Murphy , K. M. Murphy , “Dendritic Cells in Cancer Immunology,” Cellular & Molecular Immunology 19, no. 1 (2022): 3‐13. 34480145 10.1038/s41423-021-00741-5 PMC8752832

[186] F. M. Cruz , J. D. Colbert , E. Merino , B. A. Kriegsman , K. L. Rock , “The Biology and Underlying Mechanisms of Cross‐Presentation of Exogenous Antigens on MHC‐I Molecules,” Annual Review of Immunology 35 (2017): 149‐176. 10.1146/annurev-immunol-041015-055254 PMC5508990 28125356

[187] J. M. Blander , “Regulation of the Cell Biology of Antigen Cross‐Presentation,” Annual Review of Immunology 36 (2018): 717‐753. 10.1146/annurev-immunol-041015-055523 PMC6430635 29490164

[188] N. Pishesha , T. J. Harmand , H. L. Ploegh , “A Guide to Antigen Processing and Presentation,” Nature Reviews Immunology 22, no. 12 (2022): 751‐764. 10.1038/s41577-022-00707-2 35418563

[189] J. S. Blum , P. A. Wearsch , P. Cresswell , “Pathways of Antigen Processing,” Annual Review of Immunology 31 (2013): 443‐73. 10.1146/annurev-immunol-032712-095910 PMC4026165 23298205

[190] C. Moussion , L. Delamarre , “Antigen Cross‐presentation by Dendritic Cells: A Critical Axis in Cancer Immunotherapy,” Seminars in Immunology 71 (2024): 101848. 38035643 10.1016/j.smim.2023.101848

[191] T. Zhang , A. Aipire , Y. Li , C. Guo , J. Li , “Antigen Cross‐presentation in Dendric Cells: From Bench to Bedside,” Biomedicine & Pharmacotherapy = Biomedecine & Pharmacotherapie 168 (2023): 115758. 37866002 10.1016/j.biopha.2023.115758

[192] P. Nair‐Gupta , A. Baccarini , N. Tung , et al., “TLR Signals Induce Phagosomal MHC‐I Delivery From the Endosomal Recycling Compartment to Allow Cross‐presentation,” Cell 158, no. 3 (2014): 506‐521. 25083866 10.1016/j.cell.2014.04.054 PMC4212008

[193] I. Dingjan , D. R. Verboogen , L. M. Paardekooper , et al., “Lipid Peroxidation Causes Endosomal Antigen Release for Cross‐presentation,” Scientific Reports 6 (2016): 22064. 26907999 10.1038/srep22064 PMC4764948

[194] E. Childs , C. M. Henry , J. Canton , C. Reis e Sousa , “Maintenance and Loss of Endocytic Organelle Integrity: Mechanisms and Implications for Antigen Cross‐presentation,” Open Biology 11, no. 11 (2021): 210194. 34753318 10.1098/rsob.210194 PMC8580422

[195] M. Gros , E. Segura , D. C. Rookhuizen , et al., “Endocytic Membrane Repair by ESCRT‐III Controls Antigen Export to the Cytosol During Antigen Cross‐presentation,” Cell Reports 40, no. 7 (2022): 111205. 35977488 10.1016/j.celrep.2022.111205 PMC9396532

[196] E. Alspach , D. M. Lussier , A. P. Miceli , et al., “MHC‐II Neoantigens Shape Tumour Immunity and Response to Immunotherapy,” Nature 574, no. 7780 (2019): 696‐701. 31645760 10.1038/s41586-019-1671-8 PMC6858572

[197] S. E. Brightman , A. Becker , R. R. Thota , et al., “Neoantigen‐specific Stem Cell Memory‐Like CD4+ T Cells Mediate CD8+ T Cell‐dependent Immunotherapy of MHC Class II‐negative Solid Tumors,” Nature Immunology 24, no. 8 (2023): 1345‐1357. 37400675 10.1038/s41590-023-01543-9 PMC10382322

[198] A. L. Huff , G. Longway , J. T. Mitchell , et al., “CD4 T Cell–activating Neoantigens Enhance Personalized Cancer Vaccine Efficacy,” JCI Insight 8, no. 23 (2023): e174027. 38063199 10.1172/jci.insight.174027 PMC10795827

[199] R. Zander , D. Schauder , G. Xin , et al., “CD4+ T Cell Help Is Required for the Formation of a Cytolytic CD8+ T Cell Subset That Protects Against Chronic Infection and Cancer,” Immunity 51, no. 6 (2019): 1028‐1042. e4. 31810883 10.1016/j.immuni.2019.10.009 PMC6929322

[200] A. Śledzińska , M. Vila de Mucha , K. Bergerhoff , et al., “Regulatory T Cells Restrain Interleukin‐2‐ and Blimp‐1‐Dependent Acquisition of Cytotoxic Function by CD4(+) T Cells,” Immunity 52, no. 1 (2020): 151‐166. e6. 31924474 10.1016/j.immuni.2019.12.007 PMC7369640

[201] J. A. Juno , D. van Bockel , S. J. Kent , A. D. Kelleher , J. J. Zaunders , C. M. Munier , “Cytotoxic CD4 T Cells‐Friend or Foe During Viral Infection?,” Frontiers in Immunology 8 (2017): 19. 28167943 10.3389/fimmu.2017.00019 PMC5253382

[202] S. Kitano , T. Tsuji , C. Liu , et al., “Enhancement of Tumor‐reactive Cytotoxic CD4+ T Cell Responses After ipilimumab Treatment in Four Advanced Melanoma Patients,” Cancer Immunology Research 1, no. 4 (2013): 235‐44. 24396833 10.1158/2326-6066.CIR-13-0068 PMC3880021

[203] E. Laroche , S. L'Espérance , “Cancer Incidence and Mortality Among Firefighters: An Overview of Epidemiologic Systematic Reviews,” International Journal of Environmental Research and Public Health 18, no. 5 (2021): 2519. 33802629 10.3390/ijerph18052519 PMC7967542

[204] G. Espinosa‐Carrasco , E. Chiu , A. Scrivo , et al., “Intratumoral Immune Triads Are Required for Immunotherapy‐mediated Elimination of Solid Tumors,” Cancer Cell 42, no. 7 (2024): 1202‐1216. e8. 38906155 10.1016/j.ccell.2024.05.025 PMC11413804

[205] F. P. Legoux , J. B. Lim , A. W. Cauley , et al., “CD4+ T Cell Tolerance to Tissue‐Restricted Self Antigens Is Mediated by Antigen‐Specific Regulatory T Cells Rather than Deletion,” Immunity 43, no. 5 (2015): 896‐908. 26572061 10.1016/j.immuni.2015.10.011 PMC4654997

[206] M. A. Morse , W. R. Gwin 3rd , D. A. Mitchell , “Vaccine Therapies for Cancer: Then and Now,” Targeted Oncology 16, no. 2 (2021): 121‐152. 33512679 10.1007/s11523-020-00788-w PMC7845582

[207] B. Q. Tay , Q. Wright , R. Ladwa , et al., “Evolution of Cancer Vaccines‐Challenges, Achievements, and Future Directions,” Vaccines 9, no. 5 (2021): 535. 34065557 10.3390/vaccines9050535 PMC8160852

[208] O. J. Finn , “The Dawn of Vaccines for Cancer Prevention,” Nature Reviews Immunology 18, no. 3 (2018): 183‐194. 10.1038/nri.2017.140 29279613

[209] J. L. Gulley , P. M. Arlen , R. A. Madan , et al., “Immunologic and Prognostic Factors Associated With Overall Survival Employing a Poxviral‐based PSA Vaccine in Metastatic Castrate‐resistant Prostate Cancer,” Cancer Immunology, Immunotherapy : CII 59, no. 5 (2010): 663‐74. 19890632 10.1007/s00262-009-0782-8 PMC2832083

[210] S. Kreiter , M. Vormehr , N. van de Roemer , et al., “Mutant MHC Class II Epitopes Drive Therapeutic Immune Responses to Cancer,” Nature 520, no. 7549 (2015): 692‐6. 25901682 10.1038/nature14426 PMC4838069

[211] D. Sexauer , E. Gray , P. Zaenker , “Tumour‐ associated Autoantibodies as Prognostic Cancer Biomarkers‐ a Review,” Autoimmunity Reviews 21, no. 4 (2022): 103041. 35032685 10.1016/j.autrev.2022.103041

[212] D. Baumjohann , P. Brossart , “T Follicular Helper Cells: Linking Cancer Immunotherapy and Immune‐related Adverse Events,” Journal for Immunotherapy of Cancer 9, no. 6 (2021): e002588. 34112740 10.1136/jitc-2021-002588 PMC8194326

[213] T. N. Schumacher , D. S. Thommen , “Tertiary Lymphoid Structures in Cancer,” Science (New York, NY) 375, no. 6576 (2022): eabf9419. 10.1126/science.abf9419 34990248

[214] R. D. Mazor , N. Nathan , A. Gilboa , et al., “Tumor‐reactive Antibodies Evolve From Non‐binding and Autoreactive Precursors,” Cell 185, no. 7 (2022): 1208‐1222. e21. 35305314 10.1016/j.cell.2022.02.012

[215] Q. Zhang , S. Wu , “Tertiary Lymphoid Structures Are Critical for Cancer Prognosis and Therapeutic Response,” Frontiers in Immunology 13 (2022): 1063711. 36713409 10.3389/fimmu.2022.1063711 PMC9875059

[216] A. M. Johnson , B. L. Bullock , A. J. Neuwelt , et al., “Cancer Cell‐Intrinsic Expression of MHC Class II Regulates the Immune Microenvironment and Response to Anti‐PD‐1 Therapy in Lung Adenocarcinoma,” Journal of Immunology 204, no. 8 (2020): 2295‐2307. 10.4049/jimmunol.1900778 PMC7472648 32179637

[217] A. Kallingal , M. Olszewski , N. Maciejewska , W. Brankiewicz , M. Baginski , “Cancer Immune Escape: The Role of Antigen Presentation Machinery,” Journal of Cancer Research and Clinical Oncology 149, no. 10 (2023): 8131‐8141. 37031434 10.1007/s00432-023-04737-8 PMC10374767

[218] A. B. Rodriguez , V. H. Engelhard , “Insights Into Tumor‐Associated Tertiary Lymphoid Structures: Novel Targets for Antitumor Immunity and Cancer Immunotherapy,” Cancer Immunology Research 8, no. 11 (2020): 1338‐1345. 33139300 10.1158/2326-6066.CIR-20-0432 PMC7643396

[219] P. S. Ohashi , A. L. DeFranco , “Making and Breaking Tolerance,” Current Opinion in Immunology 14, no. 6 (2002): 744‐59. 12413525 10.1016/s0952-7915(02)00406-5

[220] A. Timón‐Gómez , E. Nývltová , L. A. Abriata , A. J. Vila , J. Hosler , A. Barrientos , “Mitochondrial Cytochrome c Oxidase Biogenesis: Recent Developments,” Seminars in cell & developmental biology 76 (2018): 163‐178. 28870773 10.1016/j.semcdb.2017.08.055 PMC5842095

[221] E. Kvedaraite , F. Ginhoux , “Human Dendritic Cells in Cancer,” Science Immunology 7, no. 70 (2022): eabm9409. 35363544 10.1126/sciimmunol.abm9409

[222] A. C. Villani , R. Satija , G. Reynolds , et al., “Single‐cell RNA‐seq Reveals New Types of human Blood Dendritic Cells, Monocytes, and Progenitors,” Science (New York, NY) 356, no. 6335 (2017). 10.1126/science.aah4573 PMC5775029 28428369

[223] K. Shortman , P. Sathe , D. Vremec , S. Naik , M. O'Keeffe , “Plasmacytoid Dendritic Cell Development,” Advances in Immunology 120 (2013): 105‐26. 24070382 10.1016/B978-0-12-417028-5.00004-1

[224] P. F. Rodrigues , L. Alberti‐Servera , A. Eremin , G. E. Grajales‐Reyes , R. Ivanek , R. Tussiwand , “Distinct Progenitor Lineages Contribute to the Heterogeneity of Plasmacytoid Dendritic Cells,” Nature Immunology 19, no. 7 (2018): 711‐722. 29925996 10.1038/s41590-018-0136-9 PMC7614340

[225] P. F. Rodrigues , R. Tussiwand , “Novel Concepts in Plasmacytoid Dendritic Cell (pDC) Development and Differentiation,” Molecular Immunology 126 (2020): 25‐30. 32739721 10.1016/j.molimm.2020.07.006

[226] D. P. Simmons , P. A. Wearsch , D. H. Canaday , et al., “Type I IFN Drives a Distinctive Dendritic Cell Maturation Phenotype That Allows Continued Class II MHC Synthesis and Antigen Processing,” Journal of Immunology 188, no. 7 (2012): 3116‐26. 10.4049/jimmunol.1101313 PMC3311734 22371391

[227] S. Zhang , C. Audiger , M. Chopin , S. L. Nutt , “Transcriptional Regulation of Dendritic Cell Development and Function,” Frontiers in Immunology 14 (2023): 1182553. 37520521 10.3389/fimmu.2023.1182553 PMC10382230

[228] K. Shortman , W. R. Heath , “The CD8+ Dendritic Cell Subset,” Immunological Reviews 234, no. 1 (2010): 18‐31. 20193009 10.1111/j.0105-2896.2009.00870.x

[229] M. Guilliams , C. A. Dutertre , C. L. Scott , et al., “Unsupervised High‐Dimensional Analysis Aligns Dendritic Cells Across Tissues and Species,” Immunity 45, no. 3 (2016): 669‐684. 27637149 10.1016/j.immuni.2016.08.015 PMC5040826

[230] E. Segura , J. Valladeau‐Guilemond , M. H. Donnadieu , X. Sastre‐Garau , V. Soumelis , S. Amigorena , “Characterization of Resident and Migratory Dendritic Cells in human Lymph Nodes,” The Journal of Experimental Medicine 209, no. 4 (2012): 653‐60. 22430490 10.1084/jem.20111457 PMC3328358

[231] I. Sasaki , K. Hoshino , T. Sugiyama , et al., “Spi‐B Is Critical for Plasmacytoid Dendritic Cell Function and Development,” Blood 120, no. 24 (2012): 4733‐43. 23065153 10.1182/blood-2012-06-436527

[232] M. Nagasawa , H. Schmidlin , M. G. Hazekamp , R. Schotte , B. Blom , “Development of human Plasmacytoid Dendritic Cells Depends on the Combined Action of the Basic Helix‐loop‐helix Factor E2‐2 and the Ets Factor Spi‐B,” European Journal of Immunology 38, no. 9 (2008): 2389‐400. 18792017 10.1002/eji.200838470

[233] N. Onai , K. Kurabayashi , M. Hosoi‐Amaike , et al., “A Clonogenic Progenitor With Prominent Plasmacytoid Dendritic Cell Developmental Potential,” Immunity 38, no. 5 (2013): 943‐57. 23623382 10.1016/j.immuni.2013.04.006

[234] B. Reizis , A. Bunin , H. S. Ghosh , K. L. Lewis , V. Sisirak , “Plasmacytoid Dendritic Cells: Recent Progress and Open Questions,” Annual Review of Immunology 29 (2011): 163‐83. 10.1146/annurev-immunol-031210-101345 PMC4160806 21219184

[235] E. Segura , “Review of Mouse and Human Dendritic Cell Subsets,” Methods in Molecular Biology 1423 (2016): 3‐15. 27142005 10.1007/978-1-4939-3606-9_1

[236] J. P. Böttcher , C. Reis e Sousa , “The Role of Type 1 Conventional Dendritic Cells in Cancer Immunity,” Trends in cancer 4, no. 11 (2018): 784‐792. 30352680 10.1016/j.trecan.2018.09.001 PMC6207145

[237] S. T. Ferris , V. Durai , R. Wu , et al., “cDC1 prime and Are Licensed by CD4(+) T Cells to Induce Anti‐tumour Immunity,” Nature 584, no. 7822 (2020): 624‐629. 32788723 10.1038/s41586-020-2611-3 PMC7469755

[238] M. Binnewies , A. M. Mujal , J. L. Pollack , et al., “Unleashing Type‐2 Dendritic Cells to Drive Protective Antitumor CD4(+) T Cell Immunity,” Cell 177, no. 3 (2019): 556‐571. e16. 30955881 10.1016/j.cell.2019.02.005 PMC6954108

[239] E. Segura , M. Touzot , A. Bohineust , et al., “Human Inflammatory Dendritic Cells Induce Th17 Cell Differentiation,” Immunity 38, no. 2 (2013): 336‐48. 23352235 10.1016/j.immuni.2012.10.018

[240] B. Reizis , “Plasmacytoid Dendritic Cells: Development, Regulation, and Function,” Immunity 50, no. 1 (2019): 37‐50. 30650380 10.1016/j.immuni.2018.12.027 PMC6342491

[241] A. Thiel , R. Pries , S. Jeske , T. Trenkle , B. Wollenberg , “Effect of Head and Neck Cancer Supernatant and CpG‐oligonucleotides on Migration and IFN‐alpha Production of Plasmacytoid Dendritic Cells,” Anticancer Research 29, no. 8 (2009): 3019‐25. 19661310

[242] S. I. Labidi‐Galy , V. Sisirak , P. Meeus , et al., “Quantitative and Functional Alterations of Plasmacytoid Dendritic Cells Contribute to Immune Tolerance in Ovarian Cancer,” Cancer Research 71, no. 16 (2011): 5423‐34. 21697280 10.1158/0008-5472.CAN-11-0367

[243] S. Demoulin , M. Herfs , P. Delvenne , P. Hubert , “Tumor Microenvironment Converts Plasmacytoid Dendritic Cells Into Immunosuppressive/Tolerogenic Cells: Insight Into the Molecular Mechanisms,” Journal of Leukocyte Biology 93, no. 3 (2013): 343‐52. 23136258 10.1189/jlb.0812397

[244] T. Ito , M. Yang , Y. H. Wang , et al., “Plasmacytoid Dendritic Cells Prime IL‐10‐producing T Regulatory Cells by Inducible Costimulator Ligand,” The Journal of Experimental Medicine 204, no. 1 (2007): 105‐15. 17200410 10.1084/jem.20061660 PMC2118437

[245] J. Canton , H. Blees , C. M. Henry , et al., “The Receptor DNGR‐1 Signals for Phagosomal Rupture to Promote Cross‐presentation of Dead‐cell‐associated Antigens,” Nature Immunology 22, no. 2 (2021): 140‐153. 33349708 10.1038/s41590-020-00824-x PMC7116638

[246] C. Huysamen , J. A. Willment , K. M. Dennehy , G. D. Brown , “CLEC9A is a Novel Activation C‐type Lectin‐Like Receptor Expressed on BDCA3+ Dendritic Cells and a Subset of Monocytes,” The Journal of Biological Chemistry 283, no. 24 (2008): 16693‐701. 18408006 10.1074/jbc.M709923200 PMC2562446

[247] D. Sancho , O. P. Joffre , A. M. Keller , et al., “Identification of a Dendritic Cell Receptor That Couples Sensing of Necrosis to Immunity,” Nature 458, no. 7240 (2009): 899‐903. 19219027 10.1038/nature07750 PMC2671489

[248] T. B. Geijtenbeek , “Actin' as a Death Signal,” Immunity 36, no. 4 (2012): 557‐9. 22520851 10.1016/j.immuni.2012.04.004

[249] J. G. Zhang , P. E. Czabotar , A. N. Policheni , et al., “The Dendritic Cell Receptor Clec9A Binds Damaged Cells via Exposed Actin filaments,” Immunity 36, no. 4 (2012): 646‐57. 22483802 10.1016/j.immuni.2012.03.009

[250] P. Hanč , T. Fujii , S. Iborra , et al., “Structure of the Complex of F‐Actin and DNGR‐1, a C‐Type Lectin Receptor Involved in Dendritic Cell Cross‐Presentation of Dead Cell‐Associated Antigens,” Immunity 42, no. 5 (2015): 839‐849. 25979418 10.1016/j.immuni.2015.04.009 PMC5066845

[251] R. Noubade , S. Majri‐Morrison , K. V. Tarbell , “Beyond cDC1: Emerging Roles of DC Crosstalk in Cancer Immunity,” Frontiers in Immunology 10 (2019): 1014. 31143179 10.3389/fimmu.2019.01014 PMC6521804

[252] S. T. Ferris , R. A. Ohara , F. Ou , et al., “cDC1 Vaccines Drive Tumor Rejection by Direct Presentation Independently of Host cDC1,” Cancer Immunology Research 10, no. 8 (2022): 920‐931. 35648641 10.1158/2326-6066.CIR-21-0865 PMC9357132

[253] C. Lhuillier , N. P. Rudqvist , T. Yamazaki , et al., “Radiotherapy‐exposed CD8+ and CD4+ Neoantigens Enhance Tumor Control,” The Journal of Clinical Investigation 131, no. 5 (2021): e138740. 33476307 10.1172/JCI138740 PMC7919731

[254] L. Wang , C. Lynch , S. P. Pitroda , et al., “Radiotherapy and Immunology,” The Journal of Experimental Medicine 221, no. 7 (2024): e20232101. 38771260 10.1084/jem.20232101 PMC11110906

[255] R. Wu , K. M. Murphy , “DCs at the Center of Help: Origins and Evolution of the Three‐cell‐type Hypothesis,” The Journal of Experimental Medicine 219, no. 7 (2022): e20211519. 35543702 10.1084/jem.20211519 PMC9098650

[256] C. M. Henry , C. A. Castellanos , C. Reis e Sousa , “DNGR‐1‐mediated Cross‐presentation of Dead Cell‐associated Antigens,” Seminars in Immunology 66 (2023): 101726. 36758378 10.1016/j.smim.2023.101726

[257] S. Jo , R. A. Ohara , D. J. Theisen , et al., “Shared Pathway of WDFY4‐dependent Cross‐presentation of Immune Complexes by cDC1 and cDC2,” The Journal of Experimental Medicine 222, no. 4 (2025): e20240955. 39918736 10.1084/jem.20240955 PMC11804880

[258] D. J. Theisen , D. JTt , C. G. Briseño , et al., “WDFY4 is Required for Cross‐presentation in Response to Viral and Tumor Antigens,” Science (New York, NY) 362, no. 6415 (2018): 694‐699. 10.1126/science.aat5030 PMC6655551 30409884

[259] J. H. Chen , L. T. Nieman , M. Spurrell , et al., “Human Lung Cancer Harbors Spatially Organized Stem‐immunity Hubs Associated With Response to Immunotherapy,” Nature Immunology 25, no. 4 (2024): 644‐658. 38503922 10.1038/s41590-024-01792-2 PMC12096941

[260] P. Meiser , M. A. Knolle , A. Hirschberger , et al., “A Distinct Stimulatory cDC1 Subpopulation Amplifies CD8(+) T Cell Responses in Tumors for Protective Anti‐cancer Immunity,” Cancer Cell 41, no. 8 (2023): 1498‐1515. e10. 37451271 10.1016/j.ccell.2023.06.008

[261] X. Lei , I. Khatri , T. de Wit , et al., “CD4(+) helper T Cells Endow cDC1 With Cancer‐impeding Functions in the human Tumor Micro‐environment,” Nature Communications 14, no. 1 (2023): 217. 10.1038/s41467-022-35615-5 PMC9839676 36639382

[262] X. Lei , D. C. de Groot , M. J. P. Welters , et al., “CD4(+) T Cells Produce IFN‐I to License cDC1s for Induction of Cytotoxic T‐cell Activity in human Tumors,” Cellular & Molecular Immunology 21, no. 4 (2024): 374‐392. 38383773 10.1038/s41423-024-01133-1 PMC10978876

[263] B. Maier , A. M. Leader , S. T. Chen , et al., “A Conserved Dendritic‐cell Regulatory Program Limits Antitumour Immunity,” Nature 580, no. 7802 (2020): 257‐262. 32269339 10.1038/s41586-020-2134-y PMC7787191

[264] R. Zilionis , C. Engblom , C. Pfirschke , et al., “Single‐Cell Transcriptomics of Human and Mouse Lung Cancers Reveals Conserved Myeloid Populations Across Individuals and Species,” Immunity 50, no. 5 (2019): 1317‐1334. e10. 30979687 10.1016/j.immuni.2019.03.009 PMC6620049

[265] R. Wu , R. A. Ohara , S. Jo , et al., “Mechanisms of CD40‐dependent cDC1 Licensing Beyond Costimulation,” Nature Immunology 23, no. 11 (2022): 1536‐1550. 36271147 10.1038/s41590-022-01324-w PMC9896965

[266] T. Warger , P. Osterloh , G. Rechtsteiner , et al., “Synergistic Activation of Dendritic Cells by Combined Toll‐Like Receptor Ligation Induces Superior CTL Responses in Vivo,” Blood 108, no. 2 (2006): 544‐50. 16537810 10.1182/blood-2005-10-4015

[267] M. Hubo , B. Trinschek , F. Kryczanowsky , A. Tuettenberg , K. Steinbrink , H. Jonuleit , “Costimulatory Molecules on Immunogenic versus Tolerogenic human Dendritic Cells,” Frontiers in Immunology 4 (2013): 82. 23565116 10.3389/fimmu.2013.00082 PMC3615188

[268] S. Gou , S. Wang , W. Liu , et al., “Adjuvant‐free Peptide Vaccine Targeting Clec9a on Dendritic Cells Can Induce Robust Antitumor Immune Response Through Syk/IL‐21 Axis,” Theranostics 11, no. 15 (2021): 7308‐7321. 34158852 10.7150/thno.56406 PMC8210616

[269] B. Zeng , A. P. Middelberg , A. Gemiarto , et al., “Self‐adjuvanting Nanoemulsion Targeting Dendritic Cell Receptor Clec9A Enables Antigen‐specific Immunotherapy,” The Journal of Clinical Investigation 128, no. 5 (2018): 1971‐1984. 29485973 10.1172/JCI96791 PMC5919883

[270] D. Sancho , D. Mourão‐Sá , O. P. Joffre , et al., “Tumor Therapy in Mice via Antigen Targeting to a Novel, DC‐restricted C‐type Lectin,” The Journal of Clinical Investigation 118, no. 6 (2008): 2098‐2110. 18497879 10.1172/JCI34584 PMC2391066

[271] I. Caminschi , A. I. Proietto , F. Ahmet , et al., “The Dendritic Cell Subtype‐restricted C‐type Lectin Clec9A Is a Target for Vaccine Enhancement,” Blood 112, no. 8 (2008): 3264‐73. 18669894 10.1182/blood-2008-05-155176 PMC2569177

[272] J. Bourque , D. Hawiger , “Activation, Amplification, and Ablation as Dynamic Mechanisms of Dendritic Cell Maturation,” Biology 12, no. 5 (2023): 716. 37237529 10.3390/biology12050716 PMC10215461

[273] S. Kim , J. Chen , S. Jo , et al., “IL‐6 Selectively Suppresses cDC1 Specification via C/EBPβ,” The Journal of Experimental Medicine 220, no. 10 (2023): e20221757. 37432392 10.1084/jem.20221757 PMC10336151

[274] H. Tang , Z. Wei , B. Zheng , et al., “Rescuing Dendritic Cell Interstitial Motility Sustains Antitumour Immunity,” Nature (2025): 1‐10. 10.1038/s41586-025-09202-9 40562926

[275] N. A. Ivica , C. M. Young , “Tracking the CAR‐T Revolution: Analysis of Clinical Trials of CAR‐T and TCR‐T Therapies for the Treatment of Cancer (1997‐2020),” Healthcare (Basel, Switzerland) 9, no. 8 (2021): 1062. 34442199 10.3390/healthcare9081062 PMC8392279

[276] H. de Jonge , L. Iamele , M. Maggi , G. Pessino , C. Scotti , “Anti‐Cancer Auto‐Antibodies: Roles, Applications and Open Issues,” Cancers 13, no. 4 (2021): 813. 33672007 10.3390/cancers13040813 PMC7919283

[277] O. J. Finn , “Cancer Vaccines: Between the Idea and the Reality,” Nature Reviews Immunology 3, no. 8 (2003): 630‐41. 10.1038/nri1150 12974478

[278] M. Xiao , L. Xie , G. Cao , et al., “CD4(+) T‐cell Epitope‐based Heterologous Prime‐boost Vaccination Potentiates Anti‐tumor Immunity and PD‐1/PD‐L1 Immunotherapy,” Journal for Immunotherapy of Cancer 10, no. 5 (2022): e004022. 35580929 10.1136/jitc-2021-004022 PMC9114852

[279] S. Eschweiler , J. Clarke , C. Ramírez‐Suástegui , et al., “Intratumoral Follicular Regulatory T Cells Curtail Anti‐PD‐1 Treatment Efficacy,” Nature Immunology 22, no. 8 (2021): 1052‐1063. 34168370 10.1038/s41590-021-00958-6 PMC8434898

[280] H. Lam , L. K. McNeil , H. Starobinets , et al., “An Empirical Antigen Selection Method Identifies Neoantigens That either Elicit Broad Antitumor T‐cell Responses or Drive Tumor Growth,” Cancer Discovery 11, no. 3 (2021): 696‐713. 33504579 10.1158/2159-8290.CD-20-0377

[281] H. Sultan , Y. Takeuchi , J. P. Ward , et al., “Neoantigen‐specific Cytotoxic Tr1 CD4 T Cells Suppress Cancer Immunotherapy,” Nature 632, no. 8023 (2024): 182‐191. 39048822 10.1038/s41586-024-07752-y PMC11291290

[282] Y. Li , M. Wang , X. Peng , et al., “mRNA Vaccine in Cancer Therapy: Current Advance and Future Outlook,” Clinical and Translational Medicine 13, no. 8 (2023): e1384. 37612832 10.1002/ctm2.1384 PMC10447885

[283] H. O'Brien , M. Salm , L. T. Morton , et al., “Breaking the Performance Ceiling for Neoantigen Immunogenicity Prediction,” Nature Cancer 4, no. 12 (2023): 1618‐1621. 38102360 10.1038/s43018-023-00675-z

[284] L. A. Rojas , Z. Sethna , K. C. Soares , et al., “Personalized RNA Neoantigen Vaccines Stimulate T Cells in Pancreatic Cancer,” Nature 618, no. 7963 (2023): 144‐150. 37165196 10.1038/s41586-023-06063-y PMC10171177

[285] S. J. Szymura , L. Wang , T. Zhang , et al., “Personalized Neoantigen Vaccines as Early Intervention in Untreated Patients With Lymphoplasmacytic Lymphoma: A Non‐randomized Phase 1 Trial,” Nature Communications 15, no. 1 (2024): 6874. 10.1038/s41467-024-50880-2 PMC11317512 39128904

[286] M. Yarchoan , E. J. Gane , T. U. Marron , et al., “Personalized Neoantigen Vaccine and Pembrolizumab in Advanced Hepatocellular Carcinoma: A Phase 1/2 Trial,” Nature Medicine 30, no. 4 (2024): 1044‐1053. 10.1038/s41591-024-02894-y PMC11031401 38584166

[287] E. J. Sayour , D. Boczkowski , D. A. Mitchell , S. K. Nair , “Cancer mRNA Vaccines: Clinical Advances and Future Opportunities,” Nature Reviews Clinical Oncology 21, no. 7 (2024): 489‐500. 10.1038/s41571-024-00902-1 38760500

[288] V. Anagnostou , K. N. Smith , P. M. Forde , et al., “Evolution of Neoantigen Landscape During Immune Checkpoint Blockade in Non‐Small Cell Lung Cancer,” Cancer Discovery 7, no. 3 (2017): 264‐276. 28031159 10.1158/2159-8290.CD-16-0828 PMC5733805

[289] T. Sugawara , F. Miya , T. Ishikawa , et al., “Immune Subtypes and Neoantigen‐related Immune Evasion in Advanced Colorectal Cancer,” Iscience 25, no. 2 (2022): 103740. 35128352 10.1016/j.isci.2022.103740 PMC8800070

[290] R. Rosenthal , E. L. Cadieux , R. Salgado , et al., “Neoantigen‐directed Immune Escape in Lung Cancer Evolution,” Nature 567, no. 7749 (2019): 479‐485. 30894752 10.1038/s41586-019-1032-7 PMC6954100

[291] T. Nejo , H. Matsushita , T. Karasaki , et al., “Reduced Neoantigen Expression Revealed by Longitudinal Multiomics as a Possible Immune Evasion Mechanism in Glioma,” Cancer Immunology Research 7, no. 7 (2019): 1148‐1161. 31088845 10.1158/2326-6066.CIR-18-0599

[292] A. H. Pearlman , M. S. Hwang , M. F. Konig , et al., “Targeting Public Neoantigens for Cancer Immunotherapy,” Nature Cancer 2, no. 5 (2021): 487‐497. 34676374 10.1038/s43018-021-00210-y PMC8525885

[293] T. Martinov , P. D. Greenberg , “Targeting Driver Oncogenes and Other Public Neoantigens Using T Cell Receptor‐Based Cellular Therapy,” Annual Review of Cancer Biology 7, no. 1 (2023): 331‐351. 10.1146/annurev-cancerbio-061521-082114 PMC10470615 37655310

[294] M. Shen , S. Chen , X. Han , et al., “Identification of an HLA‐A*11:01‐restricted Neoepitope of Mutant PIK3CA and Its Specific T Cell Receptors for Cancer Immunotherapy Targeting Hotspot Driver Mutations,” Cancer Immunology, Immunotherapy : CII 73, no. 8 (2024): 150. 38832948 10.1007/s00262-024-03729-y PMC11150344

[295] N. Levin , S. P. Kim , C. A. Marquardt , et al., “Neoantigen‐specific Stimulation of Tumor‐infiltrating Lymphocytes Enables Effective TCR Isolation and Expansion While Preserving Stem‐Like Memory Phenotypes,” Journal for Immunotherapy of Cancer 12, no. 5 (2024): e008645. 38816232 10.1136/jitc-2023-008645 PMC11141192

[296] J. Choi , S. P. Goulding , B. P. Conn , et al., “Systematic Discovery and Validation of T Cell Targets Directed Against Oncogenic KRAS Mutations,” Cell Reports Methods 1, no. 5 (2021): 100084. 35474673 10.1016/j.crmeth.2021.100084 PMC9017224

[297] D. C. Deniger , A. Pasetto , P. F. Robbins , et al., “T‐cell Responses to TP53 “Hotspot” Mutations and Unique Neoantigens Expressed by Human Ovarian Cancers,” Clinical Cancer Research: An Official Journal of the American Association for Cancer Research 24, no. 22 (2018): 5562‐5573. 29853601 10.1158/1078-0432.CCR-18-0573 PMC6239943

[298] P. Malekzadeh , A. Pasetto , P. F. Robbins , et al., “Neoantigen Screening Identifies Broad TP53 Mutant Immunogenicity in Patients With Epithelial Cancers,” The Journal of Clinical Investigation 129, no. 3 (2019): 1109‐1114. 30714987 10.1172/JCI123791 PMC6391139

[299] R. Foà , S. Chiaretti , “Philadelphia Chromosome‐Positive Acute Lymphoblastic Leukemia,” The New England Journal of Medicine 386, no. 25 (2022): 2399‐2411. 35731654 10.1056/NEJMra2113347

[300] A. S. Bear , T. Blanchard , J. Cesare , et al., “Biochemical and Functional Characterization of Mutant KRAS Epitopes Validates this Oncoprotein for Immunological Targeting,” Nature Communications 12, no. 1 (2021): 4365. 10.1038/s41467-021-24562-2 PMC8285372 34272369

[301] S. L. Meijer , A. Dols , W. J. Urba , et al., “Adoptive Cellular Therapy With Tumor Vaccine Draining Lymph Node Lymphocytes After Vaccination With HLA‐B7/beta2‐microglobulin Gene‐modified Autologous Tumor Cells,” Journal of Immunotherapy (Hagerstown, Md : 1997) 25, no. 4 (2002): 359‐72. 12142559 10.1097/00002371-200207000-00008

[302] S. Pant , Z. A. Wainberg , C. D. Weekes , et al., “Lymph‐node‐targeted, mKRAS‐specific Amphiphile Vaccine in Pancreatic and Colorectal Cancer: The Phase 1 AMPLIFY‐201 Trial,” Nature Medicine 30, no. 2 (2024): 531‐542. 10.1038/s41591-023-02760-3 PMC10878978 38195752

[303] X. Wang , W. Wang , S. Zou , et al., “Combination Therapy of KRAS G12V mRNA Vaccine and Pembrolizumab: Clinical Benefit in Patients With Advanced Solid Tumors,” Cell Research 34, no. 9 (2024): 661‐664. 38914844 10.1038/s41422-024-00990-9 PMC11369195

[304] A. R. Rappaport , C. Kyi , M. Lane , et al., “A Shared Neoantigen Vaccine Combined With Immune Checkpoint Blockade for Advanced Metastatic Solid Tumors: Phase 1 Trial Interim Results,” Nature Medicine 30, no. 4 (2024): 1013‐1022. 10.1038/s41591-024-02851-9 38538867

[305] N. I. Ho , L. G. M. Huis In 't Veld , T. K. Raaijmakers , G. J. Adema , “Adjuvants Enhancing Cross‐Presentation by Dendritic Cells: The Key to More Effective Vaccines?,” Frontiers in Immunology 9 (2018): 2874. 30619259 10.3389/fimmu.2018.02874 PMC6300500

[306] J. Chen , Z. Li , H. Huang , et al., “Improved Antigen Cross‐presentation by Polyethyleneimine‐based Nanoparticles,” International Journal of Nanomedicine 6 (2011): 77‐84. 21289984 10.2147/IJN.S15457 PMC3025594

[307] L. M. P. Vermeulen , S. C. De Smedt , K. Remaut , K. Braeckmans , “The Proton Sponge Hypothesis: Fable or Fact?,” European Journal of Pharmaceutics and Biopharmaceutics : Official Journal of Arbeitsgemeinschaft Fur Pharmazeutische Verfahrenstechnik eV 129 (2018): 184‐190. 10.1016/j.ejpb.2018.05.034 29859281

[308] J. T. Wilson , S. Keller , M. J. Manganiello , et al., “pH‐Responsive Nanoparticle Vaccines for Dual‐delivery of Antigens and Immunostimulatory Oligonucleotides,” ACS Nano 7, no. 5 (2013): 3912‐25. 23590591 10.1021/nn305466z PMC4042837

[309] F. C. Knight , P. Gilchuk , A. Kumar , et al., “Mucosal Immunization With a pH‐Responsive Nanoparticle Vaccine Induces Protective CD8(+) Lung‐Resident Memory T Cells,” ACS Nano 13, no. 10 (2019): 10939‐10960. 31553872 10.1021/acsnano.9b00326 PMC6832804

[310] J. P. Bost , M. Ojansivu , M. J. Munson , et al., “Novel Endosomolytic Compounds Enable Highly Potent Delivery of Antisense Oligonucleotides,” Communications Biology 5, no. 1 (2022): 185. 35233031 10.1038/s42003-022-03132-2 PMC8888659

[311] C. Li , Y. Hou , M. He , et al., “Laponite Lights Calcium Flickers by Reprogramming Lysosomes to Steer DC Migration for an Effective Antiviral CD8(+) T‐Cell Response,” Advanced Science (Weinheim, Baden‐Wurttemberg, Germany) 10, no. 30 (2023): e2303006. 37638719 10.1002/advs.202303006 PMC10602536

[312] D. Jeon , E. Hill , D. G. McNeel , “Toll‐Like Receptor Agonists as Cancer Vaccine Adjuvants,” Human Vaccines & Immunotherapeutics 20, no. 1 (2024): 2297453. 38155525 10.1080/21645515.2023.2297453 PMC10760790

[313] J. M. den Haan , S. M. Lehar , M. J. Bevan , “CD8(+) but Not CD8(‐) Dendritic Cells Cross‐prime Cytotoxic T Cells in Vivo,” The Journal of Experimental Medicine 192, no. 12 (2000): 1685‐96. 11120766 10.1084/jem.192.12.1685 PMC2213493

[314] A. Bachem , S. Güttler , E. Hartung , et al., “Superior Antigen Cross‐presentation and XCR1 Expression Define human CD11c+CD141+ Cells as Homologues of Mouse CD8+ Dendritic Cells,” The Journal of Experimental Medicine 207, no. 6 (2010): 1273‐81. 20479115 10.1084/jem.20100348 PMC2882837

[315] Y. Ding , Z. Guo , Y. Liu , et al., “The Lectin Siglec‐G Inhibits Dendritic Cell Cross‐presentation by Impairing MHC Class I‐peptide Complex Formation,” Nature Immunology 17, no. 10 (2016): 1167‐75. 27548433 10.1038/ni.3535

[316] J. Munkley , “Aberrant Sialylation in Cancer: Therapeutic Opportunities,” Cancers 14, no. 17 (2022): 4248. 36077781 10.3390/cancers14174248 PMC9454432

[317] C. Dobie , D. Skropeta , “Insights Into the Role of Sialylation in Cancer Progression and Metastasis,” British Journal of Cancer 124, no. 1 (2021): 76‐90. 33144696 10.1038/s41416-020-01126-7 PMC7782833

[318] N. Y. Z. Cheang , K. S. Tan , P. S. Tan , et al., “Single‐shot Dendritic Cell Targeting SARS‐CoV‐2 Vaccine Candidate Induces Broad, Durable and Protective Systemic and Mucosal Immunity in Mice,” Molecular Therapy: the Journal of the American Society of Gene Therapy 32, no. 7 (2024): 2299‐2315. 38715364 10.1016/j.ymthe.2024.05.003 PMC11286822

[319] E. S. Clark , A. P. Benaduce , W. N. Khan , O. Martinez , E. Gilboa , “Vaccination Against Neoantigens Induced in Cross‐priming cDC1 in Vivo,” Cancer Immunology, Immunotherapy : CII 73, no. 1 (2024): 9. 38231450 10.1007/s00262-023-03597-y PMC10794404

[320] R. Kavishna , T. Y. Kang , M. Vacca , et al., “A Single‐shot Vaccine Approach for the Universal Influenza A Vaccine Candidate M2e,” Proceedings of the National Academy of Sciences of the United States of America 119, no. 13 (2022): e2025607119. 35320040 10.1073/pnas.2025607119 PMC9060463

[321] K. A. Masterman , O. L. Haigh , K. M. Tullett , et al., “Human CLEC9A Antibodies Deliver NY‐ESO‐1 Antigen to CD141(+) Dendritic Cells to Activate Naïve and Memory NY‐ESO‐1‐specific CD8(+) T Cells,” Journal for Immunotherapy of Cancer 8, no. 2 (2020): e000691. 32737142 10.1136/jitc-2020-000691 PMC7394304

[322] Y. Zhang , J. Chen , L. Shi , F. Ma , “Polymeric Nanoparticle‐based Nanovaccines for Cancer Immunotherapy,” Mater Horiz 10, no. 2 (2023): 361‐392. 36541078 10.1039/d2mh01358d

[323] S. Morales‐Hernández , N. Ugidos‐Damboriena , J. López‐Sagaseta , “Self‐Assembling Protein Nanoparticles in the Design of Vaccines: 2022 Update,” Vaccines (Basel) 10, no. 9 (2022): 1447. 36146525 10.3390/vaccines10091447 PMC9505534

[324] F. T. Hsu , C. L. Tsai , I. T. Chiang , et al., “Synergistic Effect of Abraxane That Combines human IL15 Fused With an Albumin‐binding Domain on Murine Models of Pancreatic Ductal Adenocarcinoma,” Journal of Cellular and Molecular Medicine 26, no. 7 (2022): 1955‐1968. 35174623 10.1111/jcmm.17220 PMC8980892

[325] S. Gou , W. Liu , S. Wang , et al., “Engineered Nanovaccine Targeting Clec9a(+) Dendritic Cells Remarkably Enhances the Cancer Immunotherapy Effects of STING Agonist,” Nano Letters 21, no. 23 (2021): 9939‐9950. 34779631 10.1021/acs.nanolett.1c03243

[326] Q. Zhou , Y. Zhou , T. Li , Z. Ge , “Nanoparticle‐Mediated STING Agonist Delivery for Enhanced Cancer Immunotherapy,” Macromolecular Bioscience 21, no. 8 (2021): e2100133. 34117839 10.1002/mabi.202100133

[327] P. Liu , L. Zhao , G. Kroemer , O. Kepp , “Conventional Type 1 Dendritic Cells (cDC1) in Cancer Immunity,” Biology Direct 18, no. 1 (2023): 71. 37907944 10.1186/s13062-023-00430-5 PMC10619282

[328] M. Saxena , N. Bhardwaj , “Turbocharging Vaccines: Emerging Adjuvants for Dendritic Cell Based Therapeutic Cancer Vaccines,” Current Opinion in Immunology 47 (2017): 35‐43. 28732279 10.1016/j.coi.2017.06.003 PMC5626599

[329] J. Liu , X. Zhang , Y. Cheng , X. Cao , “Dendritic Cell Migration in Inflammation and Immunity,” Cellular & Molecular Immunology 18, no. 11 (2021): 2461‐2471. 34302064 10.1038/s41423-021-00726-4 PMC8298985

[330] F. Y. Huang , J. Y. Wang , S. Z. Dai , et al., “A Recombinant Oncolytic Newcastle Virus Expressing MIP‐3α Promotes Systemic Antitumor Immunity,” Journal for Immunotherapy of Cancer 8, no. 2 (2020): e000330. 32759233 10.1136/jitc-2019-000330 PMC7410001

[331] E. Ascic , F. Åkerström , M. Sreekumar Nair , “In Vivo Dendritic Cell Reprogramming for Cancer Immunotherapy,” Science (New York, NY) 386, no. 6719 (2024): eadn9083. 10.1126/science.adn9083 PMC7616765 39236156

[332] T. Gao , S. Yuan , S. Liang , et al., “In Situ Hydrogel Modulates cDC1‐Based Antigen Presentation and Cancer Stemness to Enhance Cancer Vaccine Efficiency,” Advanced Science (Weinheim, Baden‐Wurttemberg, Germany) 11, no. 20 (2024): e2305832. 38564766 10.1002/advs.202305832 PMC11132059

[333] M. Ashayeripanah , J. Vega‐Ramos , D. Fernandez‐Ruiz , et al., “Systemic Inflammatory Response Syndrome Triggered by Blood‐borne Pathogens Induces Prolonged Dendritic Cell Paralysis and Immunosuppression,” Cell Reports 43, no. 2 (2024): 113754. 38354086 10.1016/j.celrep.2024.113754

[334] A. Ghasemi , A. Martinez‐Usatorre , L. Li , et al., “Cytokine‐armed Dendritic Cell Progenitors for Antigen‐agnostic Cancer Immunotherapy,” Nature Cancer 5, no. 2 (2024): 240‐261. 37996514 10.1038/s43018-023-00668-y PMC10899110

